# Synthesis of divalent ligands of β-thio- and β-*N*-galactopyranosides and related lactosides and their evaluation as substrates and inhibitors of *Trypanosoma cruzi* trans-sialidase

**DOI:** 10.3762/bjoc.10.324

**Published:** 2014-12-19

**Authors:** María Emilia Cano, Rosalía Agusti, Alejandro J Cagnoni, María Florencia Tesoriero, José Kovensky, María Laura Uhrig, Rosa M de Lederkremer

**Affiliations:** 1 CIHIDECAR-CONICET, Departamento de Química Orgánica, Facultad de Ciencias Exactas y Naturales, Universidad de Buenos Aires, Pabellón 2, Ciudad Universitaria, 1428 Buenos Aires, Argentina, Fax: (+) 541145763346; 2 Laboratoire de Glycochimie, des Antimicrobiens et des Agroressources (LG2A)-CNRS FRE 3517, Université de Picardie Jules Verne, 33 rue Saint Leu, 80039 Amiens Cedex, France

**Keywords:** β-galactopyranosides, multivalent ligands, sialic acid, sugar scaffolds, *T. cruzi* trans-sialidase

## Abstract

In this work we describe the synthesis of mono- and divalent β-*N*- and β-*S*-galactopyranosides and related lactosides built on sugar scaffolds and their evaluation as substrates and inhibitors of the *Trypanosoma cruzi* trans-sialidase (TcTS). This enzyme catalyzes the transfer of sialic acid from an oligosaccharidic donor in the host, to parasite βGal*p* terminal units and it has been demonstrated that it plays an important role in the infection. Herein, the enzyme was also tested as a tool for the chemoenzymatic synthesis of sialic acid containing glycoclusters. The transfer reaction of sialic acid was performed using a recombinant TcTS and 3’-sialyllactose as sialic acid donor, in the presence of the acceptor having βGal*p* non reducing ends. The products were analyzed by high performance anion exchange chromatography with pulse amperometric detection (HPAEC-PAD). The ability of the different S-linked and N-linked glycosides to inhibit the sialic acid transfer reaction from 3’-sialyllactose to the natural substrate *N*-acetyllactosamine, was also studied. Most of the substrates behaved as good acceptors and moderate competitive inhibitors. A di-*N*-lactoside showed to be the strongest competitive inhibitor among the compounds tested (70% inhibition at equimolar concentration). The usefulness of the enzymatic trans-sialylation for the preparation of sialylated ligands was assessed by performing a preparative sialylation of a divalent substrate, which afforded the monosialylated compound as main product, together with the disialylated glycocluster.

## Introduction

*Trypanosoma cruzi*, the agent of American trypanosomiasis, affects millions of people in Latin America [1–2] and is transmitted to animals, including humans, by triatomine insects. The parasite is passed from the mother to the fetus during pregnancy, and also by blood transfusions and organ transplants [3]. North American and European countries are now at risk as a consequence of globalization and immigration, as Chagas’ disease is usually not tested in blood banks [4–6].

Terminal β-galactopyranosides (βGal*p*) present in *T. cruzi* mucins play an important role in the interaction between the parasite and the host since they are the acceptors for sialic acid transferred by the unique trans-sialidase (TcTS) [7–9] from host cells instead of using a sialyltransferase and the donor nucleotide CMP-sialic acid [10]. Although TcTS can be considered as “promiscuous” with respect to the sialyl donor and the β-galactopyranoside acceptor, it should be noted that the reaction is in fact specific in vivo. Only sialic acid-linked α(2→3) to β-galactopyranosides in glycoconjugates is transferred to terminal β-galactopyranoside units in the acceptor substrate, to construct the same type of linkage [11–12]. TcTS also transfers, efficiently, α(2→3)-linked *N*-glycolylneuraminic acid to terminal βGal*p* groups [13–14].

The search of efficient inhibitors for TcTS is an attractive field of research not only for their potential use for chemotherapy, since there is no equivalent enzymatic activity in the human host, but also because it could provide a tool for probing the biological functions of the enzyme. Given the 3D structure of TcTS [15–18], inhibitors may be directed to the sialic acid binding site or to the galactose acceptor site. Inhibitors of TcTS binding to the βGal*p* acceptor site would be highly selective, as other sialidases lack this interaction. In this direction, a group of octyl β-galactopyranosides and octyl *N*-acetyllactosaminides were described as substrates as well as inhibitors of the enzyme [19]. Also, the synthesis of the mucin oligosaccharides allowed the study of their acceptor and inhibitory properties [20–21]. Lactose derivatives were shown to be good inhibitors of the transfer of sialic acid to the natural acceptor, *N*-acetyllactosamine (LN) [22]. In particular, lactitol efficiently controlled the apoptosis triggered by TcTS [23]. The synthesis of multivalent glycoclusters designed to be high affinity ligands for specific proteins has been an active area of research during the last years [24–28]. Among them, tetravalent glycoclusters bearing β-lactosyl residues showed to have trypanocidal activity [29]. On the other hand, a recent paper described the synthesis of 1,6-linked cyclic pseudo-galacto oligosaccharides and their in vitro sialylation by recombinant TcTS [30]. Conjugation of lactose analogs with multiarm poly(ethylene glycol) increases the bioavailability in vivo [31]. Triazole-substituted β-galactopyranosides and triazole-sialyl mimetics have been synthesized by click chemistry and their inhibitory activity on the hydrolysis of 2’-(4-methylumbelliferyl)-β-D-*N*-acetylneuraminic acid by TcTS and trypanocidal activity were evaluated [32–33].

In the present work, we selected thioglycosidic and *N*-glycosidic bonds to link the acceptor sugars to a platform by click chemistry, taking into consideration that they are highly resistant to enzymatic hydrolysis [34–35]. We have previously described the synthesis of multivalent β-thiogalactopyranosides and their inhibitory activity against the β-galactosidase from *E. coli* [36–37]. The study of β-galactopyranosides as acceptor substrates for sialic acid is in general concomitant with the study of their inhibitory properties, as they usually behave as competitive acceptors. Thus, both aspects have been explored, even though our main goal was the use of the TcTS enzyme as a tool for the synthesis of sialylated biantennary β-*N*- and β-*S*-galactopyranosides and related lactosides. On the other hand, we considered imperative the purification and characterization of the sialylated products, an aspect that has not been often exploited in previous reports. A preparative method based on anion exchange chromatography using AG1X2 resin, followed by analytical HPAE-PAD chromatography was optimized. The inhibitory behavior of the substrates for the transfer of sialic acid to the natural acceptor *N*-acetyllactosamine was also evaluated.

## Results and Discussion

As part of our project on the synthesis and biological evaluation of multivalent ligands, two families of mono- and divalent structures were synthesized in order to study their ability as acceptors or inhibitors of the reaction catalyzed by the *T. cruzi* trans-sialidase. Taking into consideration that mainly ester but also glycosidic linkages are labile in biological fluids, we choose amide and thioglycosidic bonds to attach the sugar residues to the platforms.

**Scheme 1:**
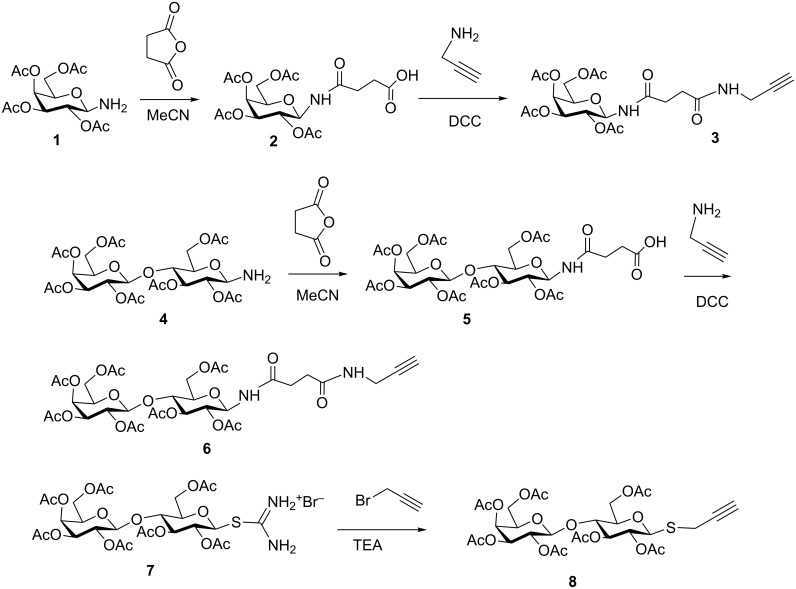
Synthesis of the alkynyl precursors **3**, **6** and **8**.

2,3,4,6-Tetra-*O*-acetyl-β-D-galactosylamine (**1**) was obtained by catalytic hydrogenation of the azide precursor and then treated with succinic anhydride to afford **2** in excellent yield, using a methodology similar to that previously described [38]. Reaction with propargylamine in the presence of DCC yielded precursor **3**. A similar procedure applied to the lactosylamine derivative **4**, led to compound **6** (Scheme 1). On the other hand, thiolactose derivative **8** was prepared by reaction of the thiouronium salt **7** [37] and propargyl bromide in the presence of triethylamine.

**Scheme 2:**
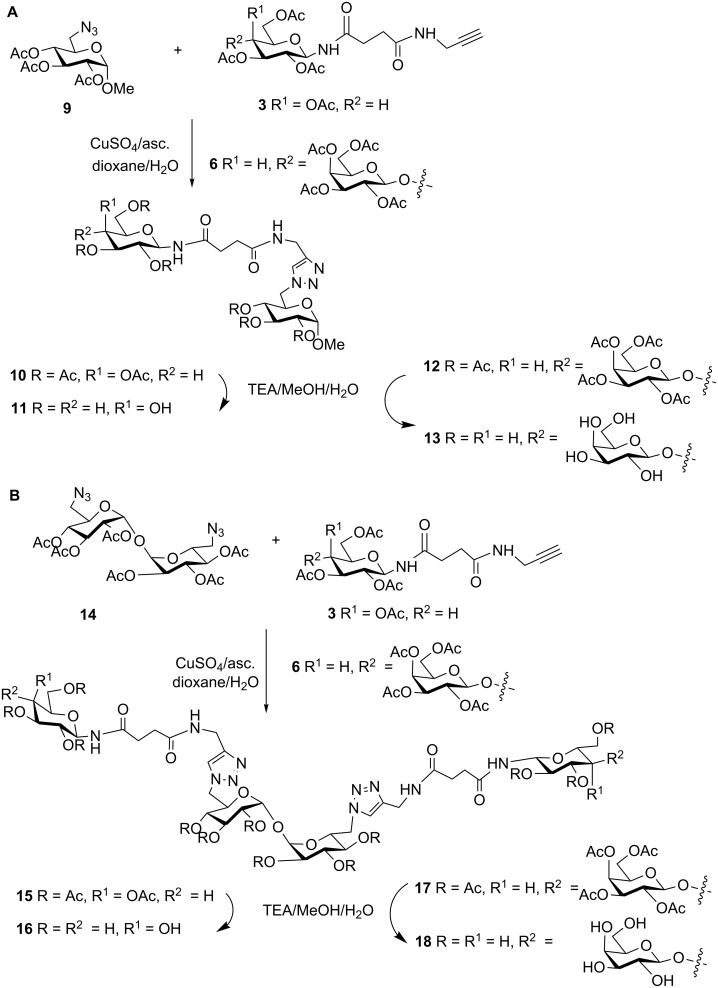
Synthesis of the mono-(A)- and di-(B)-*N*-galactopyranosides and lactosides.

Compounds **3**, **6** and **8**, functionalized with terminal alkynyl residues, were convenient precursors for the synthesis of mono- and divalent ligands based on the azide scaffolds **9** and **14**, readily available in our laboratory [36–37]. Thus, by cycloaddition reaction of monoazide **9** and *N*-galactopyranoside **3** or *N*-lactoside **6**, two monovalent derivatives (**10** and **12**, respectively) were obtained after purification by column chromatography (Scheme 2A). The ^1^H NMR spectra showed the diagnostic signals corresponding to the aromatic protons of the triazole ring (≈7.65 ppm), as well as the anomeric signals. For compound **10**, the H-1 of the β*N*Gal appeared at 5.22 ppm (*J* ≈ 9.1 Hz) and the H-1 of the αGlc at 4.92 ppm (*J* = 3.6 Hz). In the case of **12**, an additional anomeric signal corresponding to the terminal βGal residue was observed at 4.46 ppm (*J* = 7.9 Hz). Triazole carbon signals appeared at ca. 145.0 and 124.0 ppm in the ^13^C spectrum and anomeric carbons of the αGlc (96.8 ppm) scaffold, and the N-linked residue (78.5 ppm for the β*N*Gal of **10** and 78.2 for the β*N*Glc moiety of **12**) were clearly distinguishable. When precursors **3** and **6** reacted with α,α-trehalose diazide derivative **14**, the two divalent acetylated products **15** and **17** were respectively obtained (Scheme 2B). As a consequence of the symmetry of these trehalose-based divalent products, the NMR spectra of **15** showed to be similar to those of **10**, with the exception of the signal of the anomeric CH_3_O group in the case of **10**. The same observation applied for NMR spectra of compound **17**, with respect to those of **12**.

**Scheme 3:**
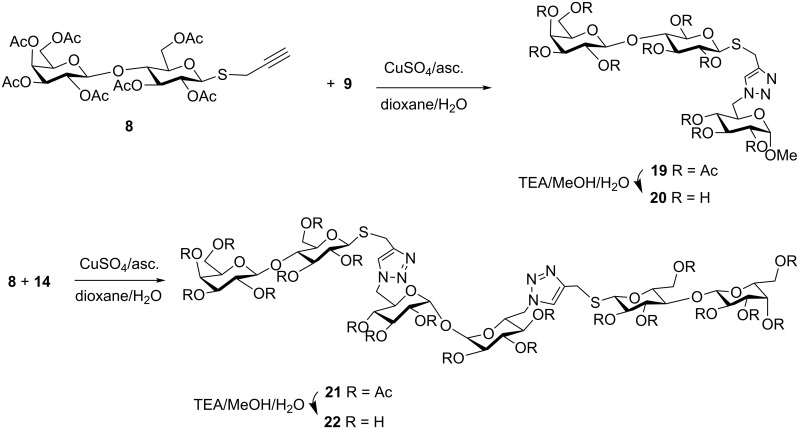
Synthesis of the mono- and di-*S*-lactosides.

On the other hand, β-thiolactosides **19** and **21** were prepared (Scheme 3). Again, they showed very similar NMR spectra. For example, in the ^13^C spectra the anomeric signals appeared at ca. 101 ppm, 91–97 ppm and 82 ppm, corresponding respectively to the terminal βGal, αGlc from the scaffold and the βSGlc residues.

*N*-linked precursors **10**, **12**, **15** and **17** and S-glycosides **19** and **21** were treated with base in mild conditions (TEA/MeOH/H_2_O), to give the final deacetylated products. The solutions were desalted using a mixed bed exchange resin and purified by passing through a reversed phase mini-column. The NMR spectra of the monovalent ligands **11**, **13** and **20**, as well as those corresponding to the divalent **16**, **18** and **22** confirmed their identity and purity.

Thiolactosides **20** and **22**, together with the thiogalactosides **23** and **24** previously reported (Table 1 and Table 2) [36], constituted thio-linked analogs to the above mentioned *N*-linked derivatives, and thus, a set of 8 structurally related mono- and divalent acceptors was available to study the trans-sialylation reaction.

### Mono- and divalent β-*N*- and β-*S*-galactopyranosides and related lactosides as sialic acid acceptors and inhibitors in the trans-sialidase reaction

**Figure 1:**
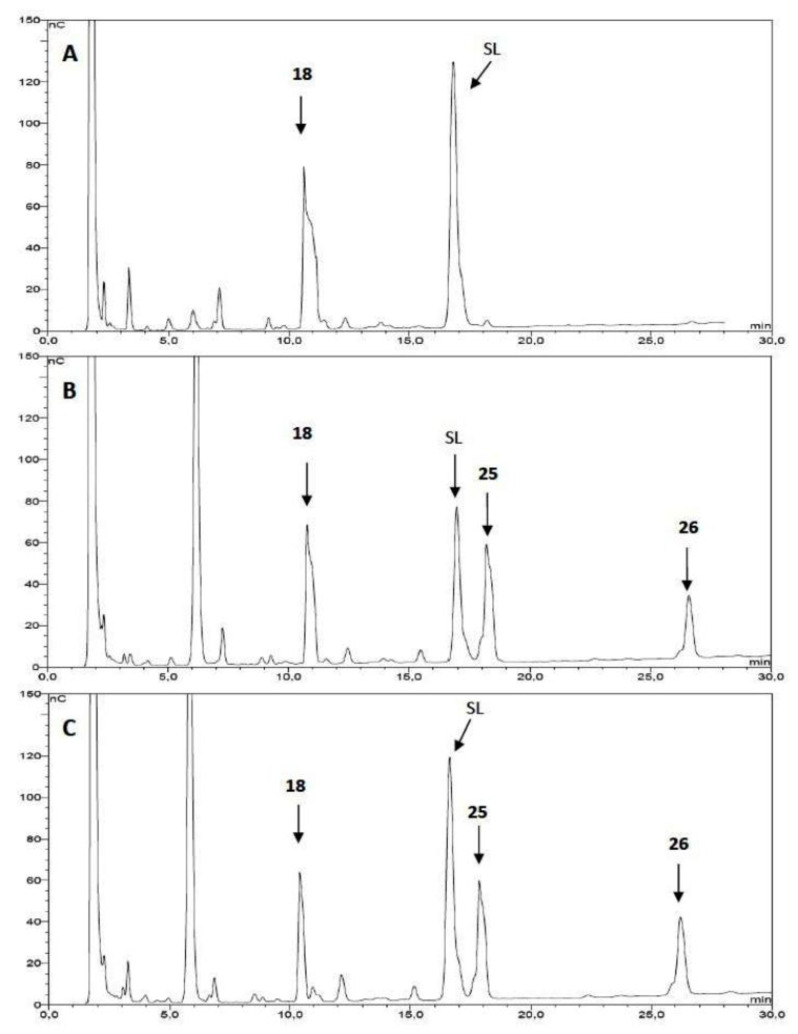
Analysis of **18** as acceptor substrate of TcTS*.* A: **18** (1 mM) and 3’-sialyllactose (SL, 1 mM), without enzyme; B: **18** (1 mM) was incubated with SL (1 mM) and TcTS for 15 min at 25 °C; C: the same as B but using 2 equivalents of SL (2 mM). The incubation mixtures were analyzed by HPAEC using a CarboPac PA-10 ion exchange analytical column eluted with a linear gradient over 30 min from 20 to 200 mM NaAcO in 100 mM NaOH at a flow rate of 0.9 mL/min. Structures for compounds **18**, **25** and **26** are shown in Scheme 4.

**Scheme 4:**
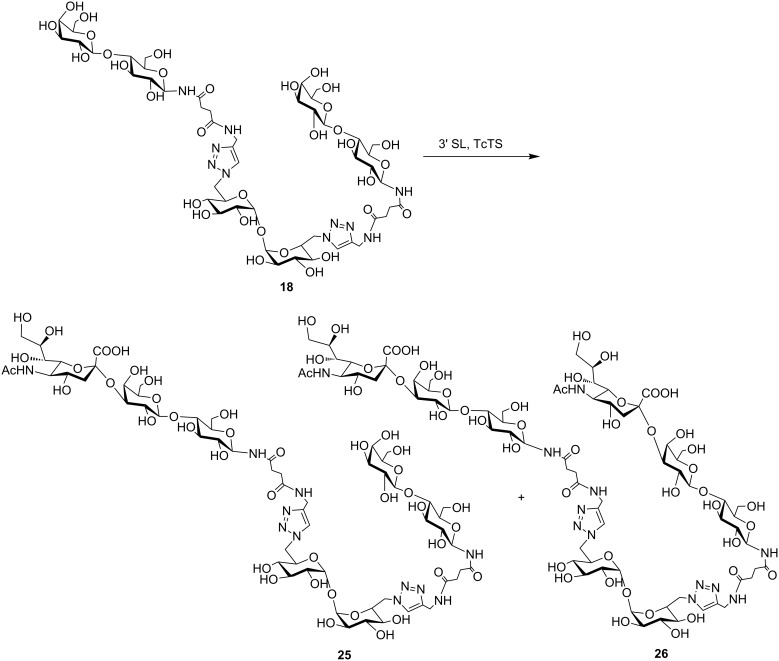
Sialylation of **18**. SL: sialyllactose.

The *N*-galactopyranoside **11**, *N*-lactoside **13**, *S*-galactopyranoside **23** [36], *S*-lactoside **20** (Table 1) and the divalent analogous **16**, **18**, **24** [36] and **22** (Table 2) were first analyzed as acceptor substrates for TcTS. The reaction is depicted for substrate **18** (Scheme 4). Conditions for incubations were as previously described [22] using 1 mM of 3’-sialyllactose (SL) as donor and 1 mM of the substrate if not otherwise indicated. The reaction was analyzed by high pH anion exchange chromatography with pulse amperometric detection (HPAEC-PAD) as shown in Figure 1 for the sialylation of **18**. In all cases, the reaction was fast and reached the equilibrium in about 15 min. All the compounds were good acceptors of sialic acid (Table 1 and Table 2). As expected, the new sialylated compounds were more retained in the anion exchange column than the original substrates. In the case of the divalent compounds **16**, **18**, **24** and **22** the disialylated compounds were also observed as minor products with the highest retention times. The extent of total sialylation reached about 60% for most of the divalent substrates (Table 2). To evaluate the disialylated species obtained from divalent substrates, experiments using 2 equivalents of SL were performed (Table 2 and Figure 1C). Although the disialylated compound was always the minor product an increase in the ratio between di- and monosialylated derivatives was evident. No significant changes were observed by prolonging the incubation times. It should be noted that, remarkably, in the case of compound **18**, almost one third of the molecules incorporated two sialyl residues. In a pioneering work using TcTS to sialylate radiolabeled alditols obtained from parasite mucins, analysis by paper electrophoresis showed that, after incorporation of the first sialyl residue, the incorporation of sialic acid on a second galactosyl unit in the same molecule was highly reduced [39]. It was then inferred that sialylation of one residue in *T. cruzi* glycans modulates the susceptibility of nearby sites, and thus, polysialylated complex multiantennary glycans would not be reachable by using the enzyme. Our results suggest that the amount of disialylated glycoclusters obtained (mainly in the case of **18**, and also for **16**, although at a lesser extent) is related to the structure of the acceptor and the experimental conditions. When both arms of the divalent precursors are sufficiently distant one from the other, they may be independently available for the enzyme. Since determinations were performed under equilibrium conditions, we cannot rule out the possibility that the percentages obtained also depend on the stability of the sialylated products that could act as donor substrates. However, incubations performed at different times between 15 and 120 min gave very similar results (not shown). A steric effect operating on the sialylation of multiple Gal*p* residues has been previously suggested for lactosyl lipids attached to membrane microdomains [40]. The dependence of the amount of disialylation of the divalent glycans on the concentration of 3’-sialyllactose in the incubation mixture was shown using equimolar or stoichiometric ratios of SL to acceptor (Table 2).

**Table 1:** Evaluation of monovalent substrates in the TcTS reaction.

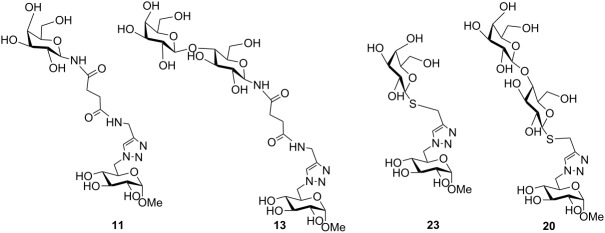			

Entry	Compound	Transfer (%)^a^	Inhibition (%)

1	**11**	47	26
2	**13**	41	32
3	**23**	52	35
4	**20**	55	41

^a^Calculated by integration of the peaks of all sialylated compounds observed in the HPAEC.

**Table 2:** Evaluation of the divalent substrates in the TcTS reaction.

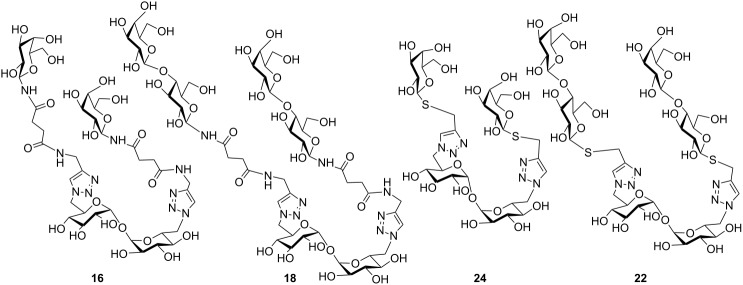										

**X**		SL:**X** = 1:1				SL:**X** = 2:1				SL:**X =** 1:1
					
		% X^a^	% S-X^a^	% S_2_-X^a^		% X^a^	% S-X^a^	% S_2_-X^a^		Inhibition (%)

**16**		35	57	8		29	49	22		16
**18**		40	40	20		33	39	28		70
**24**		53	41	12		41	47	12		48
**22**		46	46	8		29	58	13		53

^a^Related to the total amount of X (X + SX + S_2_X) by integration of the peaks observed in the HPAEC. X, compound tested, S-X, monosialylated compound X, S_2_-X, disialylated compound X, SL, sialyllactose, SL:X indicates de molar ratio of SL with respect to X.

**Figure 2:**
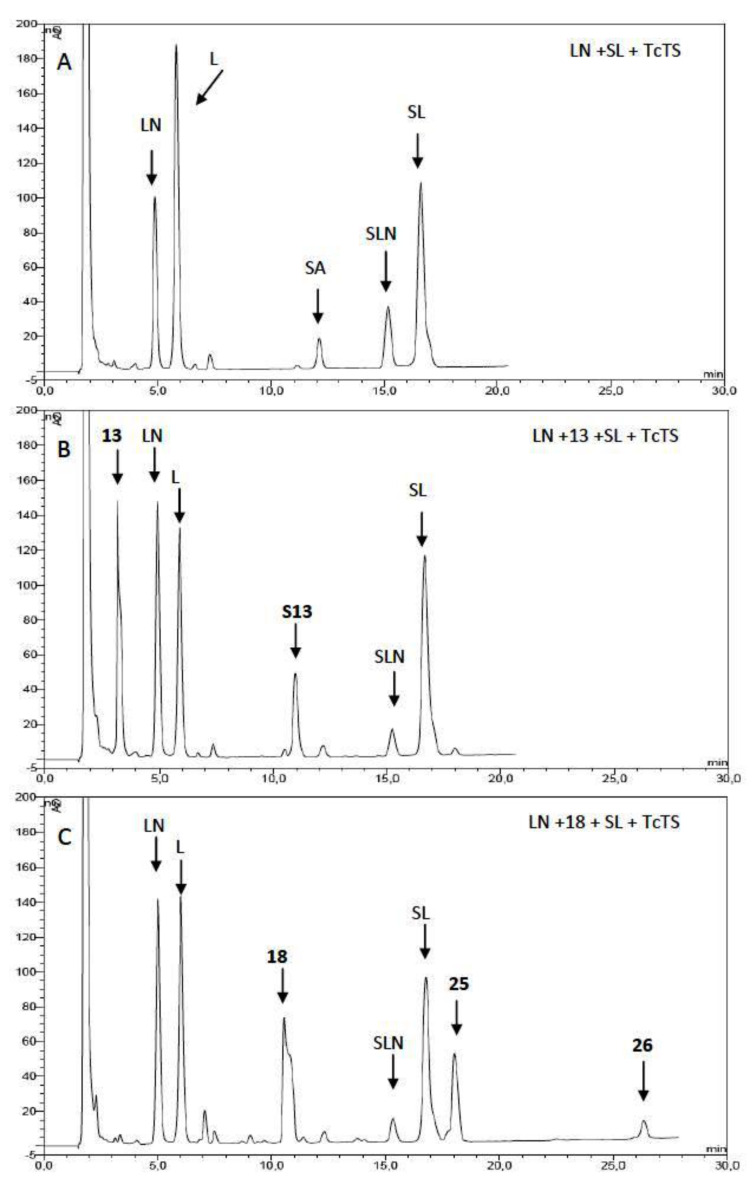
Inhibition of sialylation of LN by compounds **13** and **18**. A: *N*-acetyllactosamine (LN, 1 mM), 3’-sialyllactose (SL, 1 mM) and TcTS were incubated for 15 min at 25 °C. B: The same as A, in the presence of **13** (1 mM) as inhibitor. C: The same as A, in the presence of **18** (1 mM) as inhibitor. The incubation mixtures were analyzed by HPAEC using a CarboPac PA-10 ion exchange analytical column eluted with a linear gradient over 30 min from 20 to 200 mM NaAcO in 100 mM NaOH at a flow rate of 0.9 mL/min. L: lactose; SA: sialic acid; SLN: sialyl *N*-acetyllactosamine; **S13**: monosialyl compound **13**.

The inhibition of the sialylation of the natural substrate, *N*-acetyllactosamine (LN), by the synthetic derivatives was also studied (Table 1 and Table 2). Equimolar amounts of SL, the natural acceptor LN, and the potential inhibitors were incubated with TcTS and the reaction mixtures analyzed by HPAEC and compared with the sialylation of LN in absence of the inhibitor. An example is shown in Figure 2 for compounds **13** and **18**. The best competitive inhibitor was compound **18** which reached 70% of inhibition of transfer to LN (Table 2). In fact, **18** is a divalent compound, and so, the concentration of lactosyl groups can be considered as twice as that of **13**, which showed a 32% inhibition (Table 1). Therefore, there is no multivalent effect in the inhibition of the sialylation of LN. On the other hand, when comparing monovalent **20** (41% inhibition) and divalent **22** (53% inhibition), the latter is actually less effective per lactose residue than the monovalent **20**, showing that not even a statistical effect on the inhibition is operative. This result may be a consequence of the linker structure, a fact that can be also playing a role in the proportion of sialylated species listed above (Table 1 and Table 2). On the other hand, the multivalent effect for the inhibition of certain glycosidases was recently described [41–42]. To our knowledge, there is only one previous report on the inhibition properties of multivalent ligands on the TcTS [31].

It should be noted that by using SL as donor substrate and quantifying the new sialylated compounds we assess that only the trans-sialidase activity is measured, and not an alternative sialidase activity. Only traces of free sialic acid have been detected (Figure 2A).

**Figure 3:**
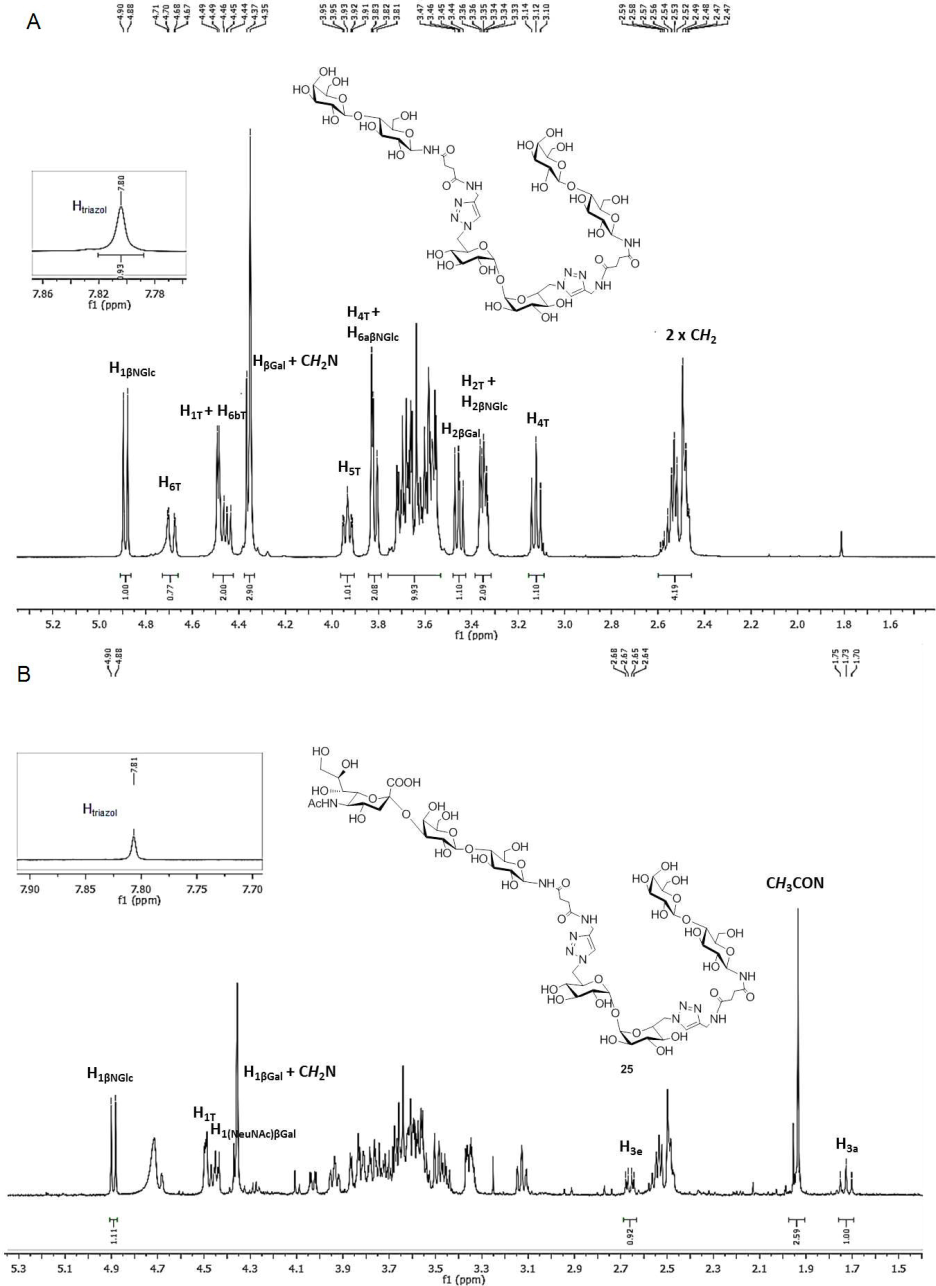
Comparison of the ^1^H NMR spectra of **18** (A) and the sialylated derivative **25** (B).

In order to prove the usefulness of the trans-sialidase reaction for the synthesis of sialylated derivatives, a preparative reaction was performed with the divalent *N*-lactoside **18**, as it was shown to be the most sensitive, among the compounds tested, to the concentration of the SL used as donor (Scheme 4). The reaction mixture, containing unreacted **18** and sialyl derivatives **25** and **26**, was purified using an AG1X2 (acetate form) resin column. After elution of neutral compounds with water, acidic derivatives were eluted with different concentrations of pyridinium acetate buffer. The eluted fractions were monitored by HPAEC. The fractions containing the monosialylated product, which appeared as a single peak at 18 min, were pooled, and **25** was characterized on the basis of the ^1^H NMR and two-dimensional HSQC spectra, by comparison with the spectrum of **18** (Figure 3A and Supporting Information File 1). The ^1^H NMR spectrum of **25** was complex, but diagnostic signals were detected (Figure 3B and Supporting Information File 1). In the anomeric region, a doublet corresponding to both anomeric protons of the two indistinguishable β-*N*-linked Glc residues appeared at 4.89 ppm (*J* = 9.3 Hz), which correlated to a ^13^C anomeric signal at 79.3 ppm in the HSQC spectrum. The two unresolved signals of both anomeric protons of αGlc residues of trehalose (T) were observed at 4.49 ppm (*J* = 3.9 Hz) and the corresponding signal at 93.4 ppm in the ^13^C spectrum was detected. Finally, two spots were observed at 4.44 and 4.36 ppm which correlated with signals at 102.6 and 103.1 ppm, respectively. These signals can be ascribed to both βGal residues, one of which is sialylated [(Neu*N*Ac)βGal and terminal βGal (Supporting Information File 1)]. The appearance of signals at 2.66 ppm (H-3eq) and 1.72 ppm (H-3ax), which correlated with a signal at 34.4 ppm (C-3, Neu*N*Ac) in the ^13^C spectrum, was also diagnostic of the sialic acid residue. Also, a singlet at 1.95 ppm, corresponding to the C*H*_3_CON group was observed. The structure of **25** was also confirmed by HRMS (ESI) with the presence of a peak at *m*/*z* 842.7806, corresponding to the [M + 2Na]^2+^ cation.

Further elution of the anion exchange column with 500 mM AcOPy gave disialylated compound **26**, which appeared as a single peak at 26 min in the HPAEC. Although only 2 mg of compound **26** were obtained, analysis by ESIMS was possible, and a peak at *m*/*z* 988.3291, corresponding to the [M + 2Na]^2+^ cation, was observed consistent with the proposed structure.

## Conclusion

Mono- and bivalent β-*N* and β-*S*-galactopyranosides and lactosides supported on sugar scaffolds were synthesized by a convergent approach using the CuAAC reaction. Monovalent as well as divalent compounds were shown to be good acceptors of sialic acid residues. Divalent substrates could also be disialylated, which means that both arms are accessible to the enzyme. By increasing the proportion of SL used as donor, a higher yield of disialylated products could be obtained and thus, this approach can be envisaged as a chemoenzymatic methodology for the synthesis of sialylated biantennary sugar derivatives. All the compounds tested were shown to be competitive inhibitors for the sialylation of the natural acceptor *N*-acetyllactosamine by TcTS. The best result was obtained for compound **18** which showed a 70% inhibition when equimolar amounts of substrates and inhibitor were used. The divalent *N*- and *S*-β-galactopyranosides and their sialylated products are potentially useful as inhibitors of other clinically relevant receptors of β-galactoside or sialic acid binding proteins. On the other hand, due to the ability of the compounds synthesized to inhibit the sialic acid transfer reaction from 3’-sialyllactose to the natural substrate *N*-acetyllactosamine, they are potential candidates for chemotherapy of Chagas’ disease, since TcTS is a fundamental enzyme in the infection process.

## Experimental

The synthetic general methods are described in the Supporting Information File 2.

**Synthesis of compounds 10, 12, 15, 17, 20 and 22. General procedure for the click reaction** [43–44]. The corresponding azido-saccharides **9** or **14** [34] (0.20 mmol) and N-linked glycosides **3** or **6**, or S-linked lactoside **8** (0.20 mmol per mol of reacting azide) were dissolved in 2.5 mL of a dioxane/H_2_O mixture (8:2). Copper sulfate (0.05 mmol per mol of reacting azide) and sodium ascorbate (0.10 mmol per mol of azide reacting group) were added, and the mixture was stirred at 70 °C under microwave irradiation during 50 min. The mixture was then poured into a 1:1 H_2_O/NH_4_Cl solution (20 mL) and extracted with EtOAc (4 × 15 mL). The organic layer was dried (Na_2_SO_4_), filtered, and the solvent was removed under reduced pressure. The residue was purified by flash chromatography, using the solvent system indicated in each case.

**Compound 17:** Compound **17** was obtained by reaction of alkyne **6** and diazide **14**. Yield: 193 mg, 44%; mp 151–152 °C; [α]_D_^20^ +12.5 (*c* 1.0, CHCl_3_); *R*_f_ 0.18 (EtOAc/MeOH 9:1); ^1^H NMR (500 MHz, CDCl_3_) δ 7.61 (H-triazole), 7.19 (d, *J*_1,NH_ = 9.3 Hz, 1H, N*H*), 6.69 (t, *J*_CH2,NH_ = 5.4 Hz, 1H, N*H*), 5.41 (t, *J*_3T,4T_ = *J*_2T,3T_ = 9.7 Hz, 1H, H-3T), 5.34 (dd, *J*_4´,5´_ = 0.7, *J*_3´,4´_ = 3.4 Hz, 1H, H-4´), 5.27 (t, *J*_2,3_ = *J*_3,4_ = 9.2 Hz, 1H, H-3), 5.24 (t, *J*_1,2_ = *J*_1,NH_ = 9.3 Hz, 1H, H-1), 5.09 (dd, *J*_1´,2´_ = 7.9, *J*_2´,3´_ = 10.4 Hz, 1H, H-2´), 4.96 (dd, *J*_1T,2T_ = 3.8, *J*_2T,3T_ = 9.9 Hz, 1H, H-2T), 4.94 (dd, *J*_3´,4´_ = 3.5, *J*_2´,3´_ = 10.4 Hz, 1H, H-3´), 4.92 (t, *J*_3T,4T_ = *J*_4T,5T_ = 9.7 Hz, 1H, H-4T), 4.87 (t, *J*_1,2_ = *J*_2,3_ = 9.5 Hz, 1H, H-2), 4.74 (d, *J*_1T,2T_ = 3.8 Hz, 1H, H-1T), 4.59 (dd, *J*_CH2,NH_ = 5.0, *J*_gem_ = 15.0 Hz, 1H, C*H*_2_N), 4.54 (dd, *J*_5T,6aT_ = 0.9, *J*_6aT,6bT_ = 13.9 Hz, 1H, H-6aT), 4.46 (d, *J*_1´,2´_ = 7.9 Hz, 1H, H-1´), 4.40 (dd, *J*_5,6a_ = 1.4, *J*_6a,6b_ = 12.1 Hz, 1H, H-6a), 4.33 (dd, *J*_CH2,NH_ = 4.5, *J*_gem_ = 15.0 Hz, 1H, C*H*_2_N), 4.26 (dd, *J*_5T,6bT_ = 9.4, *J*_6aT,6bT_ = 14.5 Hz, 1H, H-6bT), 4.14 (dd, *J*_5,6a´_ = 6.2, *J*_6a´,6b´_ = 11.1 Hz, 1H, H-6a´), 4.09–4.02 (m, 3H, H-5T, H-6b, H-6b´), 3.86 (ddd, *J*_4´,5´_ = 0.7, *J*_5´,6a´_ = 6.7, *J*_5´,6b´_ = 7.2 Hz, 1H, H-5´), 3.78 (t, *J*_3,4_ = 8.8, *J*_4,5_ = 9.9 Hz, 1H, H-4), 3.74 (ddd, *J*_5,6a_ = 1.6, *J*_5,6b_ = 4.1, *J*_4,5_ = 10.1, 1H, H-5), 2.59–2.45 (m, 4H, C*H*_2_-C*H*_2_), 2.15, 2.12, 2.06, 2.04 (3×), 2.03, 2.01 (2×), 1.96 (10 s, 30H, C*H*_3_CO); ^13^C NMR (125 MHz, CDCl_3_) δ 172.7, 171.8, 171.1, 170.5 (2×), 170.3 (2×), 170.2, 170.0, 169.9, 169.6, 169.1 (*C*OCH_3_), 145.2 (C-4 triazole), 124.1 (C-5 triazole), 101.1 (C-1´), 91.7 (C-1T), 78.1 (C-1), 76.0 (C-4), 74.5 (C-5), 72.9 (C-3), 71.1 (C-3´), 71.0 (C-2), 70.8 (C-5´), 69.9 (C-4T) 69.5 (C-5T), 69.4 (C-3T), 69.1 (C-2´), 69.0 (C-2T), 66.7 (C-4´), 62.0 (C-6), 60.9 (C-6´), 50.8 (C-6T), 35.2 (*C*H_2_NH), 31.4, 30.8 (*C*H_2_-*C*H_2_), 21.0 (2×), 20.8 (6×), 20.7 (2×) (*C*H_3_CO-); anal. calcd for C_90_H_120_N_10_O_53_·2H_2_O: C, 48.56; H, 5.61; N, 6.29; found: C, 48.20; H, 5.59; N, 6.01. HRMS–ESI (*m/z*): [M + H]^+^ calcd for C_90_H_121_N_10_O_53_, 2189.7075; found, 2189.7081.

### General procedure for O-deacetylation

Compounds **10**, **12**, **15**, **17**, **19** and **21** (0.10 mmol) were deacetylated by treatment with a solution of Et_3_N/MeOH/H_2_O 1:4:5 as previously described [45]. Further purification by a mixed bed ion-exchange resin and an octadecyl (C18) mini column was accomplished. Purity was checked by TLC (*n*-BuOH/EtOH/H_2_O, 2.5:1:1 or 1:1:1) and the corresponding R*_f_* are indicated in each case.

**Compound 18:** Yield: 126 mg, 93%; [α]_D_^20^ +40.6 (*c* 0.6, H_2_O); *R*_f_ 0.34 (BuOH/EtOH/H_2_O 1:1:1); ^1^H NMR (500 MHz, CDCl_3_) δ 7.80 (s, 1H, H-triazole), 4.89 (d, *J*_1,2_ = 9.2 Hz, 1H, H-1), 4.71 (dd, *J*_5T,6aT_ = 2.0, *J*_6aT,6bT_ = 14.5 Hz, 1H, H-6aT), 4.49 (d, *J*_1T,2T_ = 3.9 Hz, 1H, H-1T), 4.45 (dd, *J*_5T,6aT_ = 8.0, *J*_6aT,6bT_ = 14.5 Hz, 1H, H-6bT), 4.36 (d, *J*_1´,2´_ ≈ 7.8 Hz, 1H, H-1´), 4.35 (s, 2H, NH-C*H*_2_), 3.93 (ddd, *J*_5T,6aT_ = 2.2, *J*_5T,6bT_ = 7.9, *J*_4T,5T_ = 10.2 Hz, 1H, H-5T), 3.72–3.55 (m, 9H, H-3´, H-3, H-3T, H-4, H-5´, H-5, H-6a´, H-6b´, H-6b), 3.45 (dd, *J*_1´,2´_ = 7.8, *J*_2´,3´_ = 9.8 Hz, 1H, H-2´), 3.36 (dd, *J*_1T,2T_ = 3.9, *J*_2T,3T_ = 9.9 Hz, 1H, H-2T), 3.34 (t, *J*_1,2_ = *J*_2,3_ = 8.8 Hz, 1H, H-2), 3.12 (t, *J*_3T,4T_ = *J*_4T,5T_ = 9.5 Hz, 1H, H-4T), 2.59–2.47 (m, 4H, C*H*_2_-C*H*_2_); ^13^C NMR (125 MHz, D_2_O) δ 175.9, 174.6 (*C*O), 144.7 (C-4 triazole), 124.7 (C-5 triazole), 102.9 (C-1´), 93.3 (C-1T), 79.1 (C-1), 77.8, 76.3, 75.3, 75.0, 72.6, 72.5, 71.5, 70.9, 70.8 (C-2´, C-2, C-2T, C-3´, C-3, C-3T, C-4, C-5´, C-5), 70.7 (C-4T), 70.4 (C-5T), 68.5 (C-4´), 61.0 (C-6´), 59.9 (C-6), 50.8 (C-6T), 34.4 (NH-*C*H_2_), 30.8, 30.4 (*C*H_2_-*C*H_2_); Anal. calcd for C_50_H_80_N_10_O_33_·2H_2_O: C, 43.35; H, 6.11; N, 10.11; found: C, 43.04; H, 5.95; N, 9.80; HRMS–ESI (*m/z*): [M + Na]^+^ calcd for C_50_H_80_N_10_O_33_Na, 1371.4781; found, 1371.4797.

### Enzyme catalysis

Compounds **11**, **13**, **16**, **18***,* **20**, and **22–24** were incubated with TcTS in 20 mM Tris–HCl, pH 7 buffer, 30 mM NaCl, containing 1 mM 3’-sialyllactose as donor, in a similar manner as described before [46]. Analysis of the reaction mixture was performed by HPAEC-PAD. For comparison of their capacity to act as acceptors, 1 mM of each, SL and mono- or divalent substrates were used. In the case of divalent substrates, an experiment using a 2-fold excess of SL (2 mM) was also carried out. The percentage of sialylation was calculated by integration of all the sialylated species present (in the case of monovalent compounds) or by integration of mono-, di- and non-sialylated remaining species (in the case of divalent compounds).

### Inhibition of sialylation of *N*-acetyllactosamine

The inhibition experiments were performed as described before [22]. Briefly, monovalent compounds **11**, **13**, **20**, and **23**, or divalent **16**, **18**, **22**, and **24**, (1 mM) were incubated in 20 mM Tris–HCl, pH 7 buffer (20 µL), 30 mM NaCl, containing 1 mM 3’-sialyllactose as donor, 1 mM *N*-acetyllactosamine, and recombinant TcTS (300 ng) for 15 min at room temperature. After dilution with deionized water, analysis by HPAEC-PAD was performed. Inhibition was calculated considering the amount of 3’-sialyl-*N*-acetyllactosamine with respect to the total amount of sialylated compounds, obtained with or without inhibitor.

### Preparative sialylation of compound **18**

Compound **18** (10 mg, 6.5 μmol) and SL (9.0 mg, 14 μmol) were incubated with 13 μg of recombinant TcTS in 0.2 mL of 20 mM Tris buffer pH 7.6 containing 30 mM NaCl for 14 h at 25 °C. The reaction mixture was analyzed by HPAEC. The sialylated products were purified by passing through an anion exchange resin (AG1X2, acetate form, BioRad, 1.2 × 15 cm). Neutral compounds, namely **18** and lactose, were eluted with H_2_O and sialylated compounds with a stepped gradient from 50 mM to 500 mM pyridinium acetate buffer pH 5.4. Fractions (1.5 mL) were collected and analyzed by HPAEC. Compound **25**, the product of sialylation of **18**, was eluted with 100 mM pyridinium acetate while the remaining sialyllactose was eluted with 200 mM pyridinium acetate buffer. Further elution with 500 mM buffer afforded the disialylated compound **26** (2 mg). The pooled fractions were concentrated by lyophilization. Compound **25** was further purified by passing through a SepPack C8 cartridge (Alltech) eluting with H_2_O to obtain 5 mg of a colourless syrup: ^1^H NMR (500 MHz, D_2_O), partial assignments assisted by the HSQC spectrum: δ 7.81 (s, 2H, H-triazole), 4.89 (d, *J* = 9.3 Hz, 2H, H-1-βNGlc), ≈4.69 (m, under the suppressed signal of HDO, H-6aT), 4.49 (2 d superimposed, *J* = 3.9 Hz, 2H, H-1T), 4.47 (m, *J* ≈ 8.0, 14.3 Hz, 2H, H-6bT), 4.44 (d, *J* = 7.8 Hz, 1H, H-1-(NeuNAc)βGal), 4.37 (d, *J* ≈ 7.9 Hz, 1H, H-1-βGal), 4.35 (s, 4H, C*H*_2_N), 4.03 (dd, *J* = 3.1, 9.8 Hz, 1H, H-6NeuNAc), 3.94 (m, *J* = 2.3, 8.0, 10.4 Hz, 2H, H-5T), 3.87–3.45 (m, 30H), 3.37–3.33 (m, 4H, H-2T, H-2-βNGlc), 3.12 (2 t superimposed, *J* = 9.5 Hz, 2H, H-4T), 2.66 (dd, *J* = 4.6, 12.3 Hz, 1H, H-3eq-NeuNAc), 2.58–2.47 (m, 8H, 4 × C*H*_2_), 1.93 (s, 3H, C*H*_3_CON), 1.72 (t, *J* = 12.3 Hz, 1H, H-3ax-NeuNAc); ^13^C NMR (125 MHz, D_2_O), partial assignments assisted by the HSQC spectrum: δ 124.8 (*C*H-triazole), 103.1 (C-1-(NeuNAc)βGal*), 102.6 (C-1-βGal*), 93.4 (C-1T), 79.3 (C-1-βNGlc), 77.8, 77.7, 76.3, 75.4 (C-6-NeuNAc), 75.1, 72.6, 72.5, 71.6, 71.4 (C-2T*), 70.9, 70.8 (C-4T), 70.6 (C-2-βNGlc*), 70.4, 70.4 (C-5T), 69.3, 68.4, 68.1 (2×), 67.5, 62.6 (2×), 61.0, 59.8 (2×), 51.6, 50.8 (C-6T), 39.4 (C-3-NeuNAc), 34.4 (*C*H_2_N), 30.7, 30.4 (*C*H_2_-*C*H_2_), 22.2 (*C*H_3_CON); ESIMS (*m*/*z*): [M + 2Na]^2+^ calcd for C_61_H_97_N_11_Na_2_O_41_, 842.7814; found, 842.7806.

Disialylated compound **26** (2 mg) was obtained by using the anion exchange column and elution with 500 mM AcOPy: ESIMS (*m*/*z*): [M + 2Na]^2+^ calcd for C_72_H_114_N_12_Na_2_O_49_: 988.3291; found: 988.3291.

## Supporting Information

File 1 Experimental section and data of ^1^H and ^13^C NMR spectra of compounds **2**, **3**, **5**, **6**, **8**, **10–13**, **15–22** and **25**.

File 2 Copies of ^1^H and ^13^C NMR spectra of compounds **2**, **3**, **5**, **6**, **8**, **10**–**13**, **15–22** and **25**.

## References

[1] Brener Z (1973). Annu Rev Microbiol.

[2] Moncayo A (2003). Mem Inst Oswaldo Cruz.

[3] Rassi A, Rassi A, Marin-Neto J A (2010). Lancet.

[4] Montgomery S P, Starr M C, Cantey P T, Edwards M S, Meymandi S K (2014). Am J Trop Med Hyg.

[5] Gascon J, Bern C, Pinazo M-J (2010). Acta Trop.

[6] Schmunis G A, Yadon Z E (2010). Acta Trop.

[7] Buscaglia C A, Campo V A, Frasch A C C, Di Noia J M (2006). Nat Rev Microbiol.

[8] Giorgi M E, de Lederkremer R M (2011). Carbohydr Res.

[9] Meinke S, Thiem J (2012). Top Curr Chem.

[10] Schenkman S, Jiang M-S, Hart G W, Nussenzweig V (1991). Cell.

[11] Vandekerckhove F, Schenkman S, Pontes de Carvalho L, Tomlinson S, Kiso M, Yoshida M, Hasegawa A, Nussenzweig V (1992). Glycobiology.

[12] Ferrero-Garcia M A, Trombetta S E, Sánchez D O, Reglero A, Frasch A C C, Parodi A J (1993). Eur J Biochem.

[13] Agusti R, Giorgi M E, de Lederkremer R M (2007). Carbohydr Res.

[14] Schroven A, Meinke S, Ziegelmüller P, Thiem J (2007). Chem – Eur J.

[15] Buschiazzo A, Amaya M F, Cremona M L, Frasch A C C, Alzari P M (2002). Mol Cell.

[16] Amaya M F, Buschiazzo A, Nguyen T, Alzari P M (2003). J Mol Biol.

[17] Amaya M F, Watts A G, Damager I, Wehenkel A, Nguyen T, Buschiazzo A, Paris G, Frasch A C C, Withers S G, Alzari P M (2004). Structure.

[18] Meinke S, Schroven A, Thiem J (2011). Org Biomol Chem.

[19] Harrison J A, Kartha R K P, Fournier E J L, Lowary T L, Malet C, Nilsson U J, Hindsgaul O, Schenkman S, Naismith J H, Field R A (2011). Org Biomol Chem.

[20] Campo V L, Carvalho I, Allman S, Davis B G, Field R A (2007). Org Biomol Chem.

[21] de Lederkremer R M, Agusti R (2009). Adv Carbohydr Chem Biochem.

[22] Agusti R, Paris G, Ratier L, Frasch A C C, de Lederkremer R M (2004). Glycobiology.

[23] Mucci J, Risso M G, Leguizamón M S, Frasch A C C, Campetella O (2006). Cell Microbiol.

[24] Kitov P I, Sadowska J M, Mulvey G, Armstrong G D, Ling H, Pannu N S, Read R J, Bundle D R (2000). Nature.

[25] Maierhofer C, Rohmer K, Wittmann V (2007). Bioorg Med Chem.

[26] Kiessling L L, Geswicki J E, Strong L E (2006). Angew Chem, Int Ed.

[27] Chabre Y M, Roy R (2010). Adv Carbohydr Chem Biochem.

[28] Gingras M, Chabre Y M, Roy M, Roy R (2013). Chem Soc Rev.

[29] Galante E, Geraci C, Sciuto S, Campo V L, Carvalho I, Sesti-Costa R, Guedes P M M, Silva J S, Hill L, Nepogodiev S A (2011). Tetrahedron.

[30] Campo V L, Carvalho I, Da Silva C H T P, Schenkman S, Hill L, Nepogodiev S A, Field R A (2010). Chem Sci.

[31] Giorgi M E, Ratier L, Agusti R, Frasch A C C, de Lederkremer R M (2012). Glycobiology.

[32] Carvalho I, Andrade P, Campo V L, Guedes P M M, Sesti-Costa R, Silva J S, Schenkman S, Dedola S, Hill L, Rejzek M (2010). Bioorg Med Chem.

[33] Campo V L, Sesti-Costa R, Carneiro Z A, Silva J S, Schenkman S, Carvalho I (2012). Bioorg Med Chem.

[34] Driguez H (2001). ChemBioChem.

[35] Driguez H (1997). Top Curr Chem.

[36] Cagnoni A J, Varela O, Gouin S G, Kovensky J, Uhrig M L (2011). J Org Chem.

[37] Cagnoni A J, Varela O, Uhrig M L, Kovensky J (2013). Eur J Org Chem.

[38] Murphy P V, Bradley H, Tosin M, Pitt N, Fitzpatrick G M, Glass W K (2003). J Org Chem.

[39] Previato J O, Jones C, Xavier M T, Wait R, Travassos L R, Parodi A J, Mendonça-Previato L (1995). J Biol Chem.

[40] Noble G T, Craven F L, Segarra-Maset M D, Reyes Martinez J E, Šardzik R, Flitsch S L, Webb S J (2014). Org Biomol Chem.

[41] Compain P, Bodlenner A (2014). ChemBioChem.

[42] Durka M, Buffet K, Iehl J, Holler M, Nierengarten J-F, Vincent S P (2012). Chem – Eur J.

[43] Rostovtsev V V, Green L G, Fokin V V, Sharpless K B (2002). Angew Chem, Int Ed.

[44] Tornøe C W, Christensen C, Meldal M (2002). J Org Chem.

[45] Cagnoni A J, Varela O, Kovensky J, Uhrig M L (2013). Org Biomol Chem.

[46] Agusti R, Giorgi M E, Mendoza V M, Gallo-Rodriguez C, de Lederkremer R M (2007). Bioorg Med Chem.

