# Supplementary material for: Synthesis of divalent ligands of β-thio- and β-N-galactopyranosides and related lactosides and their evaluation as substrates and inhibitors of Trypanosoma cruzi trans-sialidase
Source: Beilstein J Org Chem. 2014 Dec 19;10:3073–86. doi: 10.3762/bjoc.10.324 (PMC4311708; doi:10.3762/bjoc.10.324)
Supplement: File 1 — Experimental section and data of 1H and 13C NMR spectra of compounds 2, 3, 5, 6, 8, 10–13, 15–22 and 25. [file Beilstein_J_Org_Chem-10-3073-s001.pdf]

Supporting Information File 1

for

Synthesis of divalent ligands of β-thio- and β-*N*-galactopyranosides and related lactosides and their evaluation as substrates and inhibitors of *Trypanosoma cruzi* trans-sialidase

María Emilia Cano[§,1], Rosalía Agusti[§,1], Alejandro J. Cagnoni[1], María Florencia Tesoriero[1], José Kovensky[2], María Laura Uhrig[*,1], Rosa M. de Lederkremer[*,1]

Address: [1]CIHIDECAR-CONICET, Departamento de Química Orgánica, Facultad de Ciencias Exactas y Naturales, Universidad de Buenos Aires, Pabellón 2, Ciudad Universitaria, 1428 Buenos Aires (Argentina), Fax: (+) 541145763346 and [2] Laboratoire de Glycochimie, des Antimicrobiens et des Agroressources (LG2A)-CNRS FRE 3517, Université de Picardie Jules Verne, 33 rue Saint Leu, 80039 Amiens Cedex, France.

E-mail: María Laura Uhrig - mluhrig@qo.fcen.uba.ar; Rosa M. de Lederkremer - lederk@qo.fcen.uba.ar

*Corresponding author

[§]Contributed equally to this work.

Experimental section and data of ^{1}H and ^{13}C NMR spectra of compounds 2, 3, 5, 6, 8, 10–13, 15–22 and 25.

Experimental

General methods

Analytical thin layer chromatography (TLC) was performed on Silica Gel 60 F254 aluminum supported plates (layer thickness 0.2 mm) with solvent systems given in the text. Visualization of the spots was effected by exposure to UV light and charring with a solution of 5% (v/v) sulfuric acid in EtOH, containing 0.5% *p*-anisaldehyde. Column chromatography was carried out with Silica Gel 60 (230-400 mesh). Optical rotations were measured at 20 °C in a 1 dm cell with a Perkin-Elmer 343 polarimeter. Microwave irradiation was carried out in a CEM Discover MW instrument with a System Internal IR probe type, at 70 ºC (power max 300 W). High resolution mass spectra (HRMS) were obtained by Electrospray Ionization (ESI) and Q-TOF detection with a BRUKER microTOF-Q II ESI-Q-TOF spectrometer. ^{1}H and ^{13}C nuclear magnetic resonance (NMR) spectra were recorded at 25 °C at 500 and 125 MHz, respectively, using a Bruker Avance II 500 spectrometer. For ^{1}H, ^{13}C nuclear magnetic resonance (NMR) spectra, chemical shifts are reported in parts per million relative to tetramethylsilane or a residual solvent peak ($CHCl_3$: ^{1}H: δ = 7.26 ppm, ^{13}C: δ = 77.2 ppm). Assignments of ^{1}H and ^{13}C were assisted by 2D ^{1}H–COSY and 2D ^{1}H–^{13}C experiments. In the description of the spectra, the signals corresponding to the glucose or trehalose moieties were labeled as "G" or "T", respectively. *N*-acetyllactosamine was purchased from Sigma Chemical Co. 3'-sialyllactose was obtained from bovine colostrum by an adaptation of a reported method [1]. Azide-sugar scaffolds **9** and **14**, thiouronium salt **7**, thioglycoside **19** and compounds **20**, **21**, **24** and **25** were prepared as previously reported [2,3].

For the sialylation experiments a recombinant TcTS expressed in *Escherichia coli* was kindly provided by the group of O. Campetella from Universidad Nacional General San Martín (Buenos Aires, Argentina)

Analysis by HPAEC-PAD was performed using a Dionex ICS 3000 HPLC system equipped with a pulse amperometric detector. A CarboPac PA-10 ion exchange analytical column (4 × 250 mm) equipped with a guard column PA-10 (4 × 50 mm) was used eluted with a linear gradient over 30 min from 20 to 200 mM NaOAc in 100 mM NaOH at a flow rate of 0.9 ml/min at 25 ºC.

Synthesis of the precursors

***N*-(2,3,4,6-Tetra-*O*-acetyl-β-D-galactopyranosyl) succinamic acid (2)**

To a solution of 2,3,4,6-tetra-O-acetyl-β-D-galactosylamine (**1,** 389 mg, 1.12 mmol) in anhydrous THF (5.2 mL) was added succinic anhydride (1.34 mmol, 134 mg) and triethylamine (1.12 mmol, 155 μL). The reaction proceeded for 3 h until TLC showed complete consumption of the starting material. The solution was evaporated and the products were purified by column chromatography, using Toluene : EtOAc as solvent system to obtain **2** (411 mg, 82% yield). The succinyl derivative **2** showed identical spectroscopic and physical properties as those reported in the literature [4]; R_f = 0.37 ($CHCl_3$: MeOH 10:1); ^{1}H NMR (500 MHz, $CDCl_3$) δ = 6.62 (d, 1 H, $J_{NH,1}$ = 9.0 Hz, N*H*), 5.43 (d, 1 H, $J_{3,4}$ = 2.6 Hz, H-4), 5.24 (t, 1 H, $J_{1,2}$ = $J_{1,NH}$ = 9.0 Hz, H-1), 5.13 (dd, 1 H, $J_{3,4}$ = 3.1 Hz, $J_{2,3}$ = 10.1 Hz, H-3), 5.10 (dd, 1 H, $J_{1,2}$= 8.6, $J_{2,3}$ = 10.1 Hz, H-2), 4.13 – 4.02 (m, 3 H, H-5, H-6a, H-6b), 2.78 – 2.59 (m, 4 H, CH_2-CH_2), 2.14, 2.05, 1.99 (4 s, 3 H each, CH_3CO). ^{13}C NMR (125.7 MHz, $CDCl_3$): δ = 177.3 (*C*OOH), 172.0, 171.6, 170.6, 170.2, 169.9 (5 x *C*O), 78.6 (C-1), 72.4, 70.9, 68.4, 67.3 (C-2, C-3, C-4, C-5), 61.2 (C-6), 30.7, 28.7 (*C*H_2-*C*H_2), 20.8 (2x), 20.7 (2x) (4 x COCH_3).

***N*^{1}-[2,3,4,6-Tetra-*O*-acetyl-β-D-galactopyranosyl] *N*^{4}-propargyl succinamide (3)**

To a solution of **2** (0.85 mmol, 380 mg) in anh. CH_2Cl_2, DCC (1.07 mmol, 220 mg) was added under Argon. After 20 min, propargylamine (1.02 mmol, 56.4 mg) was added and the mixture was stirred at room temperature for 24 h and then filtered. The solution was concentrated and compound **3** was purified by column chromatography using $CHCl_3$: MeOH (50:1 to 35:1) as

eluting solvent. Yield: 350 mg, 85%; mp 76-78ºC; $[\alpha]_D^{20}$ +22.5 (c = 0.6, $CHCl_3$); R_f = 0.46 ($CHCl_3$: MeOH 10:1); ^{1}H NMR (500 MHz, $CDCl_3$) δ = 6.60 (d, 1 H, $J_{1,NH}$ = 9.2 Hz, N*H*), 6.00 (t, 1 H, $J_{CH2,NH}$ = 4.8 Hz, N*H*), 5.44 (dd, 1 H, $J_{4,5}$ = 1.1, $J_{3,4}$ = 2.8 Hz, H-4), 5.26 (t, 1 H, $J_{1,2} \cong J_{1,NH} \cong$ 9.2 Hz, H-1), 5.17 – 5.12 (m, 2 H, H-2, H-3); 4.13 (dd, 1 H, $J_{5,6a}$ = 7.2, $J_{6a,6b}$ = 11.1 Hz, H-6a), 4.09 (dd, 1 H, $J_{5,6b}$ = 6.0, $J_{6a,6b}$ = 11.1 Hz, H-6b), 4.04 (ddd, 1 H, $J_{4,5}$ = 1.1, $J_{5,6a}$ = 7.2, $J_{5,6b}$ = 6.0 Hz, H-5), 4.03 (dd, 2 H, $J_{CH2,C\equiv CH}$ = 2.5, $J_{CH2,NH}$ = 4.8 Hz , CH_2N), 2.60 – 2.45 (m, 4 H, CH_2-CH_2), 2.25 (t, 1 H, $J_{CH2,C\equiv CH}$ = 2.5 Hz, C≡C*H*), 2.16, 2.09, 2.04, 2.00 (4 s, 12 H, CH_3CO). RMN-^{13}C (125.7 MHz, $CDCl_3$): δ = 172.6, 171.4, 171.3, 170.5, 170.2, 170.0 (6 ×*C*O), 79.6 (HC≡*C*), 78.6 (C-1), 72.4 (C-5), 71.8 (H*C*≡C), 71.0 (C-3), 68.3 (C-2), 67.3 (C-4), 61.3 (C-6), 31.3, 29.0 (*C*H_2-*C*H_2), 30.7 (*C*H_2N), 20.9, 20.8, 20.7 (2x) (*C*H_3CO-). Anal. Calcd for $C_{21}H_{28}N_2O_{11}$: C, 52.06; H, 5.83; N, 5.78. Found: C, 52.33; H, 5.72; N, 5.74. HRMS (ESI): *m/z* $[M+H]^+$ calcd for $C_{21}H_{29}N_2O_{11}$: 485.1766, found: 485.1761.

_N_-[(2,3,6-Tri-_O_-acetyl-4-_O_-(2,3,4,6-tetra-_O_-acetyl-β-D-galactopyranosyl)-β-D-glucopyranosyl]succinamic acid (5)

Compound **5** was prepared by reaction of **4** (432 mg, 0.68 mmol) with succinic anhydride (89 mg, 0.89 mmol) in THF (5.4 mL), in the presence of triethylamine (102 μL, 0.74 mmol) as described for **2**. Purification was performed by column chromatography using Toluene : EtOAc (3:1) containing 1% AcOH as eluting solvent. Yield: 459 mg, 92%. Compound **5** showed the same spectroscopical and physical properties described previously [5]. R_f = 0.42 (AcOH 0.5% in EtOAc); ^{1}H NMR (500 MHz, $CDCl_3$) δ = 6.43 (d, 1 H, $J_{1,NH}$ = 9.3 Hz, NH), 5.35 (d, 1 H, $J_{3',4'}$ = 3.2 Hz, H-4´), 5.29 (t, 1 H, $J_{2,3}$ = $J_{3,4}$ = 9.2 Hz, H-3), 5.21 (t, 1 H, $J_{1,2}$ = $J_{1,NH}$ = 9.3 Hz, H-1), 5.10 (dd, 1 H, $J_{1',2'}$ = 8.0, $J_{2',3'}$ = 10.3 Hz, H-2´), 4.95 (dd, 1 H, $J_{3',4'}$ = 3.4, $J_{2',3'}$ = 10.4 Hz, H-3´), 4.84 (t, 1 H, $J_{2,3}$ = $J_{1,2}$ =9.6 Hz, H-2), 4.46 (d, 1 H, $J_{1',2'}$ = 8.0 Hz, H-1´), 4.43 (d, 1 H,$J_{6a,6b}$ = 14.0 Hz , H-6a), 4.15 (dd, 1 H, $J_{5',6a'}$ = 6.6, $J_{6a',6b'}$ = 11.3 Hz, H-6a´), 4.13 (dd, 1 H, $J_{5,6b}$ = 4.5, $J_{6a,6b}$ = 12.5 Hz , H-6b), 4.07 (dd, 1 H, $J_{5',6b'}$ = 7.4, $J_{6a',6b'}$ = 11.1 Hz , H-6b´), 3.87 (t, 1 H, $J_{5',6a'}$ = 6.8, $J_{5',6b'}$ =

6.8 Hz, H-5´), 3.77 (dd, 1 H, $J_{3,4}$ = 8.9, $J_{4,5}$ = 9.8 Hz, H-4), 3.73 (dd, 1 H, $J_{5,6b}$ = 3.0, $J_{4,5}$ = 9.9 Hz, H-5), 2.75, 2.63 (2m, 1 H each, C*H*$_2$), 2.47(m, 2 H, C*H*$_2$), 2.16, 2.12, 2.07, 2.05, 2.04 (2x), 1.96 (7 s, 21H, C*H*$_3$CO); ^{13}C NMR (125 MHz, CDCl$_3$) δ =176.1 (*C*OOH), 172.0, 171.6, 170.6 (2x), 170.3 (2x), 169.5, 169.2 (8x*C*O), 101.5 (C-1´), 78.2 (C-1), 76.1 (C-4), 74.6 (C-5), 72.5 (C-3), 71.1 (C-3´), 71.0 (C-2), 70.8 (C-5´), 69.1 (C-2´), 66.7 (C-4´), 62.1 (C-6), 61.0 (C-6´), 30.9, 28.8 (*C*H$_2$-*C*H$_2$), 21.0, 20.9, 20.8 (3x), 20.7, 20.6 (*C*H$_3$CO). HRMS (ESI): *m/z* [*M*+H]$^+$ calcd for $C_{30}H_{42}NO_{20}$: 736.2279, found: 795.2295.

N^1-[(2,3,6-Tri-*O*-acetyl-4-*O*-(2,3,4,6-tetra-*O*-acetyl-β-D-galactopyranosyl)-β-D-glucopyranosyl] N^4-propargyl succinamide (6)

Compound **6** was prepared by reaction of **5** with propargylamine in freshly distilled anh. CH_2Cl_2, in the presence of DCC, as described for **3**. Purification by column chromatography (Cl_3CH : MeOH, 50:1 to 35:1) afforded 360 mg of **6** (yield 81%); mp 104-105 °C; $[\alpha]_D^{20}$ +5.6 (*c* = 0.8, CHCl$_3$); R_f = 0.53 (CHCl$_3$: MeOH 10:1); ^{1}H NMR (500 MHz, CDCl$_3$) δ = 6.64 (d, 1 H, $J_{1,NH}$ = 9.2 Hz, N*H*), 6.09 (t, 1 H, $J_{CH2,NH}$ = 5.5 Hz, N*H*), 5.35 (dd, 1 H, $J_{4',5'}$ = 0.9, $J_{3',4'}$ = 3.4 Hz, H-4´), 5.27 (t, 1 H, $J_{2,3}$ = $J_{3,4}$ = 9.3 Hz, H-3), 5.19 (t, 1 H, $J_{1,2}$ = $J_{1,NH}$ = 9.3 Hz, H-1), 5.09 (dd, 1 H, $J_{1',2'}$ = 7.9, $J_{2',3'}$ = 10.4 Hz, H-2´), 4.94 (dd, 1 H, $J_{3',4'}$ = 3.5, $J_{2',3'}$ = 10.4 Hz, H-3´), 4.84 (t, 1 H, $J_{2,3}$ = $J_{1,2}$ =9.3 Hz, H-2), 4.46 (d, 1 H, $J_{1',2'}$ = 7.9 Hz, H-1´), 4.43 (dd, 1 H, $J_{5,6a}$ = 1.7, $J_{6a,6b}$ = 12.3 Hz , H-6a), 4.14 (dd, 1 H, $J_{5',6a'}$ = 6.4, $J_{6a',6b'}$ = 11.0 Hz , H-6a´), 4.12 (dd, 1 H, $J_{5,6b}$ = 4.3, $J_{6a,6b}$ = 12.1 Hz, H-6b), 4.07 (dd, 1 H, $J_{5',6b'}$ = 7.4, $J_{6a',6b'}$ = 11.1 Hz, H-6b´), 4.01 (dd, 2 H, $J_{CH2,C\equiv CH}$ = 2.6, $J_{CH2,NH}$ = 5.3 Hz, C*H*$_2$N), 3.86 (ddd, 1 H, $J_{4',5'}$ = 0.8, $J_{5',6a'}$ = 6.5, $J_{5',6b'}$ = 7.4 Hz, H-5´), 3.77 (t, 1 H, $J_{3,4}$ = 8.9, $J_{4,5}$ = 9.9 Hz, H-4), 3.72 (ddd, 1 H, $J_{5,6a}$ = 1.8, $J_{5,6b}$ = 4.3, $J_{4,5}$ = 9.9, H-5), 2.58 – 2.42(m, 4 H, C*H*$_2$-C*H*$_2$), 2.22 (t, 1 H, $J_{CH2,C\equiv CH}$ = 2.5 Hz, C≡C*H*), 2.15, 2.11, 2.06 (2x), 2.04, 2.03, 1.96 (7 s, 21H, C*H*$_3$CO); ^{13}C NMR (125 MHz, CDCl$_3$) δ =172.4, 171.2 (2x), 170.3 (2x), 170.1, 170.0, 169.4, 169.0 (-O*C*OCH$_3$), 100.9 (C-1´), 79.4 (*C*≡CH), 78.0 (C-1), 75.9 (C-4), 74.4 (C-5), 72.4 (C-3), 71.7 (C≡*C*H), 71.0 (C-3´), 70.8 (C-2), 70.7 (C-5´), 69.0 (C-2´),

66.6 (C-4´), 61.9 (C-6), 60.8 (C-6´), 31.2, 30.6 (CH_2-CH_2), 29.2 (CH_2N), 20.9, 20.8, 20.7, 20.6 (2×), 20.5 (CH_3CO). Anal. calcd for $C_{33}H_{44}N_2O_{19}.H_2O$: C, 50.13; H, 5.86; N, 3.54. Found: C, 50.38; H, 5.95; N, 3.79. HRMS (ESI): *m/z* $[M+Na]^+$ calcd for $C_{33}H_{44}N_2NaO_{19}$: 795.2430, found: 795.2406.

Propargyl 2,3,6-tri-*O*-acetyl-4-*O*-(2,3,4,6-tetra-*O*-acetyl-β-D-galactopyranosyl)-1-thio-β-D-glucopyranoside (8)

4-*O*-(2,3,4,6-tetra-O-acetyl-β-D-galactopyranosyl)-2,3,6-tri-O-acetyl-β-D-glucopyranosyl isothiouronium bromide [6] **7** (1.100 g, 1.420 mmol) and propargyl bromide (2.130 mmol) were dissolved in anhydrous acetonitrile (5 mL) and NEt_3 (496 μL, 3.550 mmol) was added dropwise. The resulting mixture was stirred at rt for 3 h, when TLC and MS indicated complete consumption of the starting material. The solvent was evaporated under reduced pressure and the residue was purified by flash chromatography (Hexane : EtOAc 2:3). Yield: 687 mg, 70%; $[\alpha]_D^{20}$ +43.2 (c = 1.0, $CHCl_3$); R_f = 0.46 (Hexane : EtOAc 1:1.5); ^{1}H NMR (500 MHz, $CDCl_3$) δ = 5.36 (dd,1 H, $J_{4',5'}$ = 0.9, $J_{3',4'}$ = 3.4 Hz, H-4´), 5.27 (t, 1 H, $J_{2,3}$ = $J_{3,4}$ = 9.2 Hz, H-3), 5.13 (dd, 1 H, $J_{1',2'}$ = 7.9, $J_{2',3'}$ = 10.4 Hz, H-2´), 5.00 (t, 1 H, $J_{1,2}$ = $J_{2,3}$ = 10.0 Hz, H-2), 4.97 (dd, 1 H, $J_{3',4'}$ = 3.4, $J_{2',3'}$ = 10.5 Hz, H-3´), 4.77 (d, 1 H, $J_{1,2}$ = 10.1 Hz, H-1), 4.53 (dd, 1 H, $J_{5,6a}$ = 2.0, $J_{6a,6b}$ = 12.1 Hz, H-6a), 4.50 (d, 1 H, $J_{1',2'}$ = 7.9 Hz, H-1´), 4.17−4.08 (m, 3 H, H-6b, H-6´a, H-6´b), 3.89 (ddd, 1 H, $J_{4',5'}$ = 0.9, $J_{5',6'a}$ =$J_{5',6'b}$ = 7.3 Hz, H-5´), 3.83 (t, 1 H, $J_{3,4}$ = $J_{4,5}$ = 9.6 Hz, H-4), 3.67 (ddd, 1 H, $J_{5,6a}$ = 2.0, $J_{5,6b}$ = 5.3, $J_{4,5}$ = 9.9 Hz, H-5), 3.55, 3.28 (2 dd, 2 H, J = 2.6, J_{gem} = 12.6 Hz, CH_2S), 2.28 (t, 1 H, J = 2.6 Hz, C≡CH), 2.17, 2.14, 2.08, 2.07, 2.06, 2.05, 1.98 (7 s, 21 H, CH_3CO); ^{13}C NMR (125 MHz, $CDCl_3$) δ =170.3, 170.2, 170.1, 169.8, 169.7, 169.2, 169.1 (*C*OCH_3), 101.1 (C-1´), 81.7 (C-1), 78.7 (*C*≡CH), 76.8 (C-5), 76.1 (C-4), 73.7 (C-3), 71.9 (C≡*C*H), 71.0 (C-3´), 70.7 (C-5´), 70.2 (C-2), 69.1 (C-2´), 66.6 (C-4´), 62.0 (C-6), 60.9 (C-6´), 20.9, 20.8, 20.7, 20.6 (2×), 20.5 (2×) (CH_3CO-), 17.6 (CH_2S). Anal.calcd for $C_{29}H_{38}O_{17}S$: C, 50.43; H, 5.55; S, 4.64. Found: C, 50.19; H, 5.49; S, 4.88. HRMS (ESI): *m/z* $[M+Na]^+$ calcd for $C_{29}H_{38}NaO_{17}S$: 713.1722, found: 713.1720.

General procedure for the click reaction: synthesis of compounds **10**, **12**, **15**, **17**, **20** and **22**. The corresponding azido-saccharides **9** or **14** [2,3] (0.20 mmol) and N-linked glycosides **3** or **6**, or S-linked lactoside **8** (0.20 mmol per mole of reacting azide) were dissolved in 2.5 mL of a dioxane/H_2O mixture (8:2). Copper sulfate (0.05 mmol per mole of reacting azide) and sodium ascorbate (0.10 mmol per mole of azide reacting group) were added, and the mixture was stirred at 70 ºC under microwave irradiation during 50 min. The mixture was then poured into a 1:1 H_2O/NH_4Cl solution (20 mL) and extracted with EtOAc (4 ×15 mL). The organic layer was dried (Na_2SO_4) and filtered, and the solvent was removed under reduced pressure. The residue was purified by flash chromatography, using the solvent system indicated in each case.

Compound 10

Compound **10** was obtained by reaction of alkyne **3** and azide **9**. Yield: 95 mg, 57 %; mp 109-110ºC; $[\alpha]_D^{20}$ +67.9 (c = 0.3, $CHCl_3$); R_f = 0.42 ($CHCl_3$: MeOH 9:1); ^{1}H NMR (500 MHz, $CDCl_3$) δ = 7.67 (br s, 1 H, H-triazole), 6.64 (d, 1 H, $J_{1,NH}$ = 9.2 Hz, N*H*), 6.40 (br s, 1 H, N*H*), 5.47 (dd, 1 H, $J_{3G,4G}$ = 9.4, $J_{2G,3G}$ = 10.1Hz, H-3G), 5.43 (dd, 1 H, $J_{4,5}$ = 0.9, $J_{3,4}$ = 2.9 Hz, H-4), 5.22 (t, 1 H, $J_{1,2}$ = $J_{1,NH}$ = 9.1 Hz, H-1), 5.15 – 5.09 (m, 2 H, H-2, H-3), 4.92 (d, 1 H, $J_{1G,2G}$ = 3.6 Hz, H-1G), 4.82 (dd, 1 H, $J_{1G,2G}$ = 3.6, $J_{2G,3G}$ = 10.3 Hz, H-2G), 4.80 (dd, 1 H, $J_{3G,4G}$ = 9.0, $J_{4G,5G}$ = 10.2 Hz, H-4G), 4.56 – 4.37 (m, 4 H, H-6aG, H-6bG, CH_2N), 4.17 (ddd, 1 H, $J_{5G,6aG}$ = 1.9, $J_{5G,6bG}$ = 8.0, $J_{4G,5G}$ = 9.9 Hz, H-5G), 4.14 – 4.02 (m, 3 H, H-5, H-6a, H-6b), 3.15 (s, 3 H, OCH_3), 2.58 – 2.43 (m, 4 H, CH_2-CH_2), 2.14, 2.10, 2.07 (2x), 2.03, 2.00, 1.99 (7 s, 21 H, CH_3CO). RMN-^{13}C (125.7 MHz, $CDCl_3$): δ = 172.3, 171.4, 171.3, 170.4, 170.2, 170.0, 169.9, 169.8 (2x) (*C*O), 96.6 (C-1G), 78.5 (C-1), 72.2 (C-5), 70.8 (C-2G), 70.7 (C-3), 69.9 (C-4G), 69.7 (C-3G), 68.2 (C-2), 67.7 (C-5G), 67.2 (C-4), 61.1 (C-6), 55.5 (O*C*H_3), 50.7 (C-6G), 35.1 (NH-*C*H_2), 31.2, 30.6 (*C*H_2-*C*H_2), 20.8, 20.7 (3x), 20.6 (2x), 20.5 (*C*H_3CO). HRMS (ESI): *m/z* $[M+H]^+$ calcd for $C_{34}H_{48}N_5O_{19}$: 830.2938, found: 830.2954.

Compound 12

Compound **12** was obtained by reaction of alkyne **6** and azide **9**. Yield: 134 mg, 60%; mp 120 °C; $[\alpha]_D^{20}$ +43.4 (c = 0.9, $CHCl_3$); R_f = 0.31 (EtOAc : MeOH 9:1); ^{1}H NMR (500 MHz, $CDCl_3$) δ = 7.64 (s, 1 H, H-triazole), 6.61 (d, 1 H, $J_{1,NH}$ = 9.2 Hz, N*H*), 6.41 (t, 1 H, $J_{CH2,NH}$ = 5.2 Hz, N*H*), 5.47 (t, 1 H, $J_{3G,4G} = J_{2G,3G}$ = 9.7 Hz, H-3G), 5.35 (dd, 1 H, $J_{4',5'}$ = 0.9, $J_{3',4'}$ = 3.2 Hz, H-4´), 5.28 (t, 1 H, $J_{2,3} = J_{3,4}$ = 9.5 Hz, H-3), 5.19 (t, 1 H, $J_{1,2} = J_{1,NH}$ = 9.3 Hz, H-1), 5.10 (dd, 1 H, $J_{1',2'}$ = 7.9, $J_{2',3'}$ = 10.4 Hz, H-2´), 4.94 (dd, 1 H, $J_{3',4'}$ = 3.5, $J_{2',3'}$ = 10.4 Hz, H-3´), 4.92 (d, 1 H, $J_{1G,2G}$ = 3.6 Hz, H-1G), 4.83 (t, 1 H,$J_{1,2}= J_{2,3}$ =9.5 Hz, H-2), 4.82 (dd, 1 H, $J_{1G,2G}$ = 3.4, $J_{2G,3G}$ = 9.4 Hz, H-2G), 4.79 (t, 1 H, $J_{3G,4G} = J_{4G,5G}$ = 9.5 Hz, H-4G), 4.56 – 4.40 (m, 5 H, CH_2N, H-6aG, H-6bG, H-6a), 4.46 (d, 1 H, $J_{1',2'}$ = 7.9 Hz, H-1´), 4.17– 4.40 (m, 3 H, H-5G, H6b, H6a´), 4.07 (dd, 1 H, $J_{5',6b'}$ = 7.4, $J_{6a',6b'}$ = 11.1 Hz , H-6b´), 3.88 (ddd, 1 H, $J_{4',5'}$ = 0.8, $J_{5',6a'}$ = 6.4, $J_{5',6b'}$ = 7.4 Hz, H-5´), 3.77 (t, 1 H, $J_{3,4}$ = 8.9, $J_{4,5}$ = 9.9 Hz, H-4), 3.75 (ddd, 1 H, $J_{5,6a}$ = 2.0, $J_{5,6b}$ = 4.6, $J_{4,5}$ = 10.1, H-5), 3.15 (s, 3 H, OCH_3), 2.53 – 2.46 (m, 4 H, CH_2-CH_2), 2.15, 2.11, 2.10, 2.06 (2x), 2.05, 2.04 (2x), 2.00, 1.96 (10 s, 30 H, CH_3CO); ^{13}C NMR (125 MHz, $CDCl_3$) δ = 172.4, 171.7, 171.3, 170.5 (2x), 170.3 (2x), 170.2, 170.1, 170.0, 169.5, 169.1 (*C*O), 144.8 (C-4 triazole), 123.8 (C-5 triazole), 101.1 (C-1´), 96.8 (C-1G), 78.2 (C-1), 76.1 (C-4), 74.6 (C-5), 72.6 (C-3), 71.2, 71.0, 70.9, 70.8 (C-2, C-2G, C-3, C-5´), 70.0, 69.9 (C-3G, C-4G), 69.1 (C-2´), 67.8 (C-5G), 66.8 (C-4´), 62.1 (C-6), 61.0 (C-6´), 55.6 (O*C*H_3), 50.8 (C-6G), 35.2 (*C*H_2N), 31.5, 30.8 (*C*H_2-*C*H_2), 21.0, 20.9, 20.8 (6x), 20.8, 20.7, (*C*H_3CO). HRMS (ESI): *m/z* $[M+H]^+$ calcd for $C_{46}H_{64}N_5O_{27}$: 1118.3783, found: 1118.3790.

Compound 15

Compound **15** was obtained by reaction of alkyne **3** and diazide **14**. Yield: 197 mg, 61%; mp 149-151ºC; $[\alpha]_D^{20}$ +28.2 (c = 1.0, $CHCl_3$); R_f = 0.35 ($CHCl_3$: MeOH 10:1); ^{1}H NMR (500 MHz, $CDCl_3$) δ = 7.62 (s, 1 H, H-triazole), 7.13 (d, 1 H, $J_{1,NH}$ = 9.0 Hz, N*H*), 6.70 (dd, 1 H, $J_{CH2,NH}$ = 5.4 Hz, N*H*), 5.41 (dd, 1 H, $J_{3T,4T}$ = 9.4, $J_{2T,3T}$ = 10.3 Hz, H-3T), 5.42 (d, 1 H, $J_{3,4}$ = 3.0 Hz, H-4), 5.29 (t, 1 H, $J_{1,2} = J_{1,NH}$ = 9.0 Hz, H-1), 5.16 (dd, 1 H, $J_{3,4}$ = 3.3, $J_{2,3}$ = 10.3 Hz, H-3), 5.12 (dd, 1

H, $J_{1,2}$ = 9.0, $J_{2,3}$ = 10.3 Hz, H-2), 4.96 (dd, 1 H, $J_{1T,2T}$ = 3.9, $J_{2T,3T}$ = 10.4 Hz, H-2T), 4.94 (dd, 1 H, $J_{3T,4T}$ = 9.5, $J_{4T,5T}$ = 10.0 Hz, H-4T), 4.75 (d, 1 H, $J_{1T,2T}$ = 3.8 Hz, H-1T), 4.61 (dd, 1 H, $J_{CH2,NH}$ = 6.2, J_{gem}= 15.0 Hz, C*H*$_2$N), 4.56 (dd, 1 H, $J_{5T,6aT}$ =1.4, $J_{6aT,6bT}$ = 14.4 Hz, H-6aT), 4.36 (dd, 1 H, $J_{CH2,NH}$ = 4.6, J_{gem}= 15.0 Hz, C*H*$_2$N), 4.25 (dd, 1 H, $J_{5T,6bT}$ = 9.4, $J_{6aT,6bT}$ = 14.4 Hz, H-6bT), 4.06 – 3.98 (m, 4 H, H-5T, H-5, H-6^a, H-6b), 2.60 – 2.50 (m, 4 H, C*H*$_2$-C*H*$_2$), 2.12, 2.11, 2.05, 2.01, 2.00 (×2), 1.98 (7 s, 21 H, C*H*$_3$CO). RMN-^{13}C (125.7 MHz, CDCl$_3$): δ = 172.7, 171.8, 171.2, 170.5, 170.3, 170.2, 170.0 (×3) (*C*O), 145.3 (C-4 triazole); 124.0 (C-5 triazole), 91.7 (C-1T), 78.6 (C-1), 72.3 (C-5), 71.1 (C-3), 69.8 (C-3T), 69.6 (C-4T), 69.4 (C-5T), 69.0 (C-2T), 68.4 (C-2), 67.3 (C-4), 61.2 (C-6), 50.6 (C-6T), 35.2 (NH-*C*H$_2$), 31.3, 29.4 (*C*H$_2$-*C*H$_2$), 20.9 (2x), 20.8 (2x), 20.7 (3x) (*C*H$_3$CO). Anal. calcd for $C_{66}H_{88}N_{10}O_{37}$: C, 49.13; H, 5.50; N, 8.68. Found: C, 49.01; H, 5.63; N, 8.45. HRMS (ESI) m/z [M + Na]$^+$ calcd for $C_{66}H_{88}N_{10}NaO_{37}$ 1635.5204, found 1635.5204.

Compound 17

Compound **17** was obtained by reaction of alkyne **6** and diazide **14**. Yield: 193 mg, 44%; mp 151-152 °C; $[\alpha]_D^{20}$ +12.5 (*c* = 1.0, CHCl$_3$); R_f = 0.18 (EtOAc : MeOH 9:1); ^{1}H NMR (500 MHz, CDCl$_3$) δ = 7.61 (H-triazole), 7.19 (d, 1 H, $J_{1,NH}$ = 9.3 Hz, N*H*), 6.69 (t, 1 H, $J_{CH2,NH}$ = 5.4 Hz, N*H*), 5.41 (t, 1 H, $J_{3T,4T}$ = $J_{2T,3T}$ = 9.7 Hz, H-3T), 5.34 (dd, 1 H, $J_{4',5'}$ = 0.7, $J_{3',4'}$ = 3.4 Hz, H-4´), 5.27 (t, 1 H, $J_{2,3}$ = $J_{3,4}$ = 9.2 Hz, H-3), 5.24 (t, 1 H, $J_{1,2}$ = $J_{1,NH}$ = 9.3 Hz, H-1), 5.09 (dd, 1 H, $J_{1',2'}$ = 7.9, $J_{2',3'}$ = 10.4 Hz, H-2´), 4.96 (dd, 1 H, $J_{1T,2T}$ = 3.8, $J_{2T,3T}$ = 9.9 Hz, H-2T), 4.94 (dd, 1 H, $J_{3',4'}$ = 3.5, $J_{2',3'}$ = 10.4 Hz, H-3´), 4.92 (t, 1 H, $J_{3T,4T}$ = $J_{4T,5T}$ = 9.7 Hz, H-4T), 4.87 (t, 1 H,$J_{1,2}$= $J_{2,3}$ =9.5 Hz, H-2),4.74 (d, 1 H, $J_{1T,2T}$ = 3.8 Hz, H-1T), 4.59 (dd, 1 H, $J_{CH2,NH}$ = 5.0, J_{gem} = 15.0 Hz, C*H*$_2$N), 4.54 (dd, 1 H, $J_{5T,6aT}$ = 0.9, $J_{6aT,6bT}$ = 13.9 Hz, H-6aT), 4.46 (d, 1 H,$J_{1',2'}$ = 7.9 Hz, H-1´), 4.40 (dd, 1 H, $J_{5,6a}$ = 1.4, $J_{6a,6b}$ = 12.1 Hz, H-6a), 4.33 (dd, 1 H, $J_{CH2,NH}$ = 4.5, J_{gem} = 15.0 Hz, C*H*$_2$N), 4.26 (dd, 1 H, $J_{5T,6bT}$ = 9.4,$J_{6aT,6bT}$ = 14.5 Hz, H-6bT), 4.14 (dd, 1 H, $J_{5,6a'}$ = 6.2, $J_{6a',6b'}$ = 11.1 Hz, H-6a´), 4.09 – 4.02 (m, 3 H, H-5T, H-6b, H-6b´), 3.86 (ddd, 1 H, $J_{4',5'}$ = 0.7, $J_{5',6a'}$ =

6.7, $J_{5',6b'}$ = 7.2 Hz, H-5´), 3.78 (t, 1 H, $J_{3,4}$ = 8.8, $J_{4,5}$ = 9.9 Hz, H-4), 3.74 (ddd, 1 H, $J_{5,6a}$ = 1.6, $J_{5,6b}$ = 4.1, $J_{4,5}$ = 10.1, H-5), 2.59 – 2.45 (m, 4 H, CH_2-CH_2), 2.15, 2.12, 2.06, 2.04 (3x), 2.03, 2.01 (2x), 1.96 (10 s, 30 H, CH_3CO); ^{13}C NMR (125 MHz, CDCl$_3$) δ =172.7, 171.8, 171.1, 170.5 (2x), 170.3 (2x), 170.2, 170.0, 169.9, 169.6, 169.1 (*C*OCH$_3$), 145.2 (C-4 triazole), 124.1 (C-5 triazole), 101.1 (C-1´), 91.7 (C-1T), 78.1 (C-1), 76.0 (C-4), 74.5 (C-5), 72.9 (C-3), 71.1 (C-3´), 71.0 (C-2), 70.8 (C-5´), 69.9 (C-4T) 69.5 (C-5T), 69.4 (C-3T), 69.1 (C-2´), 69.0 (C-2T), 66.7 (C-4´), 62.0 (C-6), 60.9 (C-6´), 50.8 (C-6T), 35.2 (*C*H$_2$NH), 31.4, 30.8 (*C*H$_2$-*C*H$_2$), 21.0 (2x), 20.8 (6x), 20.7 (2x) (*C*H$_3$CO-). Anal. calcd for $C_{90}H_{120}N_{10}O_{53}.2H_2O$: C, 48.56; H, 5.61; N, 6.29. Found: C, 48.20; H, 5.59; N, 6.01. HRMS (ESI): *m/z* [*M*+H]$^+$calcd for $C_{90}H_{121}N_{10}O_{53}$: 2189.7075, found: 2189.7081.

Compound 19

Compound **19** was obtained by reaction of alkyne **8** and azide **9**. Yield: 184 mg, 89%; $[\alpha]_D^{20}$ +6.6 (*c* = 1.0, CHCl$_3$); R_f = 0.25 (Hexane : EtOAc 1:3); ^{1}H NMR (500 MHz, CDCl$_3$) δ = 7.62 (H-triazole), 5.48 (dd,1H, $J_{3G,4G}$ = 9.5 = $J_{2G,3G}$ = 10.0 Hz, H-3G), 5.36 (dd, 1 H, $J_{4',5'}$ = 1.0, $J_{3',4'}$ = 3.5 Hz, H-4´), 5.19 (t, 1 H, $J_{2,3}$ = $J_{3,4}$ = 9.2 Hz, H-3), 5.07 (dd, 1 H, $J_{1',2'}$ = 7.9, $J_{2',3'}$ = 10.4 Hz, H-2´), 4.97 (t, 1 H, $J_{1,2}$ = $J_{2,3}$ = 10.0 Hz, H-2), 4.96 (dd, 1 H, $J_{3',4'}$ = 3.5, $J_{2',3'}$ = 10.4 Hz, H-3´), 4.93 (d,1 H, $J_{1G,2G}$ = 3.8 Hz, H-1G), 4.81 (dd, 1 H, $J_{1G,2G}$ = 3.7, $J_{2G,3G}$ = 10.2 Hz, H-2G), 4.76 (dd, 1 H, $J_{3G,4G}$ = 9.5, $J_{4G,5G}$ = 10.2 Hz, H-4G), 4.57 (dd, 1 H, $J_{5G,6aG}$ = 2.4, $J_{6aG,6bG}$ = 14.5 Hz, H-6aG), 4.53 (d, 1 H, $J_{1,2}$ = 10.2 Hz, H-1), 4.52 (dd, 1 H, $J_{5,6a}$ = 1.7, $J_{6a,6b}$ = 12.0 Hz, H-6a), 4.48 (d, 1 H, $J_{1',2'}$ = 7.9 Hz, H-1´), 4.45 (dd, 1 H, $J_{5G,6bG}$ = 7.8, $J_{6aG,6bG}$ = 14.5 Hz, H-6bG), 4.21(ddd, 1 H, $J_{5G,6aG}$ = 2.4, $J_{5G,6bG}$ = 7.9, $J_{4G,5G}$ = 10.1 Hz, H-5G), 4.16−4.06 (m, 4 H, H-6b, H-6´a, H-6´b, C*H*S), 3.88(m, 2 H, H-5´, C*H*S), 3.81 (t, 1 H, $J_{3,4}$ = $J_{4,5}$ = 9.5 Hz, H-4), 3.64 (ddd, 1 H, $J_{5,6a}$ = 1.9, $J_{5,6b}$ = 4.7, $J_{4,5}$ = 9.9 Hz, H-5), 3.19 (s, 3 H, CH_3O), 2.15, 2.13, 2.10, 2.07, 2.06, 2.05, 2.04, 2.02, 2.01, 1.96 (10 s, 30 H, CH_3CO); ^{13}C NMR (125 MHz, CDCl$_3$) δ =170.3, 170.2, 170.1, 169.9, 169.8, 169.7, 169.6, 169.5, 169.4, 169.0 (CH$_3$*C*O), 145.0 (C-4 triazole), 123.6 (C-3 triazole), 101.1 (C-1´), 96.7 (C-1G), 82.4 (C-1), 76.6 (C-5), 76.1 (C-4), 73.6 (C-3), 71.0 (C-3´),

70.8, 70.7 (C-5´, C-2G), 70.2 (C-2), 69.6 (2×) (C-3G, C-4G), 69.0 (C-2´), 67.6 (C-5G), 66.6 (C-4´), 61.9 (C-6), 60.8 (C-6´), 55.6 (CH_3O), 50.7 (C-6G), 24.3 (CH_2S), 20.9, 20.8, 20.7(2×), 20.6 (2×), 20.5 (2×), 20.4, 20.3 (CH_3CO). Anal. calcd for $C_{42}H_{57}N_3O_{25}S$: C, 48.69; H, 5.55; N, 4.06; S, 3.10. Found: C, 48.43; H, 5.59; N, 4.12; S, 2.88. HRMS (ESI):*m/z* $[M+H]^+$ calcd for $C_{42}H_{58}N_3O_{25}S$:1036.3075, found: 1036.3104.

Compound 21

Compound **21** was obtained by reaction of alkyne **8** and diazide **14**. Yield: 251 mg, 62%; $[\alpha]_D^{20}$ +12.5 (c = 0.3, $CHCl_3$); R_f = 0.24 (Hexane : EtOAc 1:6); ^{1}H NMR (500 MHz, $CDCl_3$) δ = 7.52 (H-triazole), 5.43 (t, 1 H, $J_{2T,3T} = J_{3T,4T}$ = 9.5 Hz, H-3T), 5.32 (dd, 1 H, $J_{4´,5´}$ = 1.0, $J_{3´,4´}$ = 3.5 Hz, H-4´), 5.17 (t, 1 H, $J_{2,3} = J_{3,4}$ = 9.2 Hz, H-3), 5.06 (dd, 1 H, $J_{1´,2´}$ = 8.0, $J_{2´,3´}$ = 10.3 Hz, H-2´), 4.98−4.90 (m, 4 H, H-2, H-3´, H-1T, H-2T), 4.82 (dd, 1 H, $J_{3T,4T}$ = 9.7, $J_{4GT,5T}$ = 10.2 Hz, H-4T), 4.57−4.45 (m, 4 H, H-1, H-6a, H-1´,H-6aT), 4.36 (dd, 1 H, $J_{5T,6bT}$ = 7.7, $J_{6aT,6bT}$ = 14.1 Hz, H-6bT), 4.25 (m, 1 H, H-5T), 4.13−4.03 (m, 4 H, H-6b, H-6´a, H-6´b, C*H*S), 3.90−3.78(m, 3 H, H-4, H-5´, C*H*S), 3.63 (ddd, 1 H, $J_{5,6a}$ = 1.9, $J_{5,6b}$ = 4.7, $J_{4,5}$ = 9.9 Hz, H-5), 2.14, 2.13, 2.11, 2.08, 2.06, 2.05, 2.04, 2.02, 2.01, 1.95 (10 s, 30 H, CH_3CO); ^{13}C NMR (125 MHz, $CDCl_3$) δ = 170.3, 170.2, 170.1, 169.9, 169.8, 169.7, 169.6, 169.5, 169.4, 169.0 (CH_3CO), 145.2 (C-4 triazole), 123.4 (C-3 triazole), 101.1 (C-1´), 91.9 (C-1T), 82.1 (C-1), 76.6 (C-5), 76.1 (C-4), 73.7 (C-3), 71.0 (C-3´), 70.7 (C-2T), 70.3 (C-5´), 69.6, 69.5(C-3T, C-4T), 69.0(2×) (C-2, C-2´), 68.8 (C-5T), 66.6 (C-4´), 61.8 (C-6), 60.8 (C-6´), 50.4 (C-6T), 24.1 (CH_2S), 20.9, 20.8, 20.7 (2×), 20.6 (2×), 20.5 (2×), 20.4, 20.3 (CH_3CO). Anal. calcd for $C_{82}H_{108}N_6O_{49}S_2$: C, 48.61; H, 5.37; N, 4.15; S, 3.17. Found: C, 48.43; H, 5.47; N, 4.03; S, 3.02. HRMS (ESI): *m/z* $[M+2Na]^{2+}$ calcd for $C_{82}H_{108}N_6Na_2O_{49}S_2$: 1035.2685, found: 1035.2700.

General procedure for *O*-deacetylation: Compounds **10**, **12**, **15**, **17**, **19** and **21** (0.10 mmol) were deacetylated by treatment with a solution of Et_3N : MeOH : H_2O 1:4:5 as previously described [7]. Further purification by a mixed bed ion-exchange resin and an octadecyl (C18)

mini column was accomplished. Purity was checked by TLC (*n*-BuOH : EtOH : H_2O, 2.5:1:1 or 1:1:1) and the corresponding R_f are indicated in each case.

Compound 11

Yield: 49 mg, 92%; $[\alpha]_D^{20}$ +35.0 (*c* = 0.5, H_2O); R_f = 0.44 (BuOH : EtOH : H_2O 1:1:1); ^1H NMR (500 MHz, D_2O) δ = 7.95 (s, 1 H, H-triazole), 4.90 (t, 1 H, $J_{1,2}$ = 9.0 Hz, H-1), 4.82 (dd, 1 H, $J_{5G,6aG}$ = 2.5, $J_{6aG,6bG}$ = 14.7Hz, H-6aG), 4.73 (d, 1 H, $J_{1G,2G}$ = 3.8 Hz, H-1G), 4.60 (dd, 1 H, $J_{5G,6bG}$ = 8.1, $J_{6aG,6bG}$ = 14.6 Hz, H-6bG), 4.45 (s, 2 H, CH_2N), 3.97 (dd, 1 H, $J_{4,5}$ = 0.5, $J_{3,4}$ = 3.3 Hz, H-4), 3.91 (ddd, 1 H, $J_{5G,6aG}$ = 2.3, $J_{5G,6bG}$ = 8.2, $J_{4G,5G}$ = 10.3 Hz, H-5G), 3.78 – 3.61 (m, 6 H, H-2, H-3, H-3G, H-5, H-6a, H-6b), 3.51 (dd, 1 H, $J_{1G,2G}$ = 3.8, $J_{2G,3G}$ = 9.8 Hz, H-2G), 3.23 (dd, 1 H, $J_{3G,4G}$ = 9.0, $J_{4G,5G}$ = 10.0 Hz, H-4G), 3.11 (s, 3 H, CH_3O), 2.68 – 2.56 (m, 4 H, CH_2-CH_2). RMN-^{13}C (125.7 MHz, D_2O): δ = 175.9, 174.6 (*C*O), 144.8 (C-4 triazole), 124.7 (C-5 triazole), 99.1 (C-1G), 79.7 (C-1), 76.7, 73.3, 73.0, 69.3 (C-2, C-3, C-3G, C-5), 71.3 (C-2G), 70.8 (C-4G), 69.9 (C-5G), 68.6 (C-4), 60.9 (C-6), 54.7 (O*C*H_3), 50.9 (C-6G), 34.4 (*C*H_2N), 30.7, 30.3 (*C*H_2-*C*H_2). Anal. Calcd for $C_{20}H_{33}N_5O_{12}.H_2O$: C, 43.40; H, 6.37; N, 12.65. Found: C, 43.19; H, 6.34; N, 12.21. HRMS (ESI): *m/z* $[M+H]^+$ calcd for $C_{20}H_{34}N_5O_{12}$: 536.2198, found: 536.2210.

Compound 13

Yield: 53mg, 76%; $[\alpha]_D^{20}$ +54.5 (*c* = 0.9, H_2O); R_f = 0.53 (BuOH : EtOH : H_2O 1:1:1); ^1H NMR (500 MHz, D_2O) δ = 7.86 (s, 1 H, H-triazole), 4.88 (d, 1 H, $J_{1,2}$ = 9.2 Hz, H-1), 4.73 (dd, 1 H, $J_{5G,6aG}$ = 2.4, $J_{6aG,6bG}$ = 14.6 Hz, H-6aG), 4.63 (d, 1 H, $J_{1G,2G}$ = 3.8 Hz, H-1G), 4.50 (dd, 1 H, $J_{5G,6aG}$ = 8.2, $J_{6aG,6bG}$ = 14.6 Hz, H-6bG), 4.36 (d, 1 H, $J_{1',2'} \cong$ 6.9 Hz, H-1´), 4.35 (s, 2 H, CH_2N), 3.84 – 3.80 (m, 3 H, H-4´, H-5G, H6a), 3.72 – 3.54 (m, 9 H, H-3´, H-3, H-3G, H-4, H-5´, H-5, H-6a´, H-6b´, H-6b), 3.45 (dd, 1 H, $J_{1',2'}$ = 7.8, $J_{2',3'}$ = 9.9 Hz, H-2´), 3.43 (dd, 1 H, $J_{1G,2G}$ = 3.8, $J_{2G,3G}$ = 10.0 Hz, H-2G), 3.34 (t, 1 H,$J_{1,2}$ = $J_{2,3}$ = 9.2 Hz, H-2), 3.13 (t, 1 H, $J_{3G,4G}$ = $J_{4G,5G}$ = 9.4 Hz, H-4G), 3.01 (s, 3 H, CH_3O), 2.58 – 2.46 (m, 4 H, CH_2-CH_2); ^{13}C NMR (125 MHz, D_2O) δ =175.9, 174.6 (*C*O), 144.8 (C-4 triazole), 124.8 (C-5 triazole), 102.9 (C-1´), 99.1 (C-1G), 79.1

(C-1), 77.8, 76.3, 75.3, 75.0, 73.1, 72.5, 71.5, 71.0, 70.9 (C-2´, C-2, C-2G, C-3´, C-3, C-3G, C-4, C-5´, C-5), 70.8 (C-4G), 69.9 (C-5G), 68.5 (C-4´), 61.0 (C-6´), 59.9 (C-6), 55.7 (CH_3O), 50.9 (C-6G), 34.4 (CH_2N), 30.7, 30.3 (CH_2-CH_2). HRMS (ESI): *m/z* [*M*+H]$^+$ calcd for $C_{26}H_{44}N_5O_{17}$: 698.2727, found: 698.2730.

Compound 16

Yield: 89 mg, 87%; $[\alpha]_D^{20}$ +54.3 (*c* = 1.0, H_2O); R_f = 0.38 (BuOH : EtOH : H_2O 1:1:1); ^{1}H NMR (500 MHz, D_2O) δ = 7.91 (s, 1 H, H-triazole), 4.92 (d, 1 H, $J_{1,2}$ = 9.2 Hz, H-1), 4.82 – 4.79 (H-6aT + HDO), 4.60 (d, 1 H, $J_{1T,2T}$ = 4.0 Hz, H-1T), 4.56 (dd, 1 H, $J_{5T,6bT}$ = 8.0, $J_{6aT,6bT}$ = 14.6 Hz, H-6bT), 4.46 (s, 1 H, CH_2N), 4.05 (ddd, 1 H, $J_{5T,6aT}$ = 2.2, $J_{5T,6bT}$ = 8.0, $J_{4T,5T}$ = 10.2 Hz, H-5T), 4,03 (dd, 1 H, $J_{4,5}$ = 0.9, $J_{3,4}$ = 3.0 Hz, H-4), 3.80 – 3.71 (m, 5 H, H-3, H-3T, H-5, H-6a, H-6b), 3.64 (t, 1 H, $J_{1,2}$ = 9.2, $J_{2,3}$ = 9.2 Hz, H-2), 3,46 (dd, 1 H, $J_{1T,2T}$ = 4.0, $J_{2T,3T}$ = 10.4 Hz, H-2T), 3.24 (dd, 1 H, $J_{3T,4T}$ = 9.2, $J_{4T,5T}$ = 10.2 Hz, H-4T), 2.70 – 2.58 (m, 4 H, CH_2-CH_2). RMN-^{13}C (125.7 MHz, D_2O): δ (ppm) = 176.0, 174.6 (*C*O), 144.7 (C-4 triazole), 124.7 (C-5 triazole), 93.3 (C-1T), 79.7 (C-1), 76.7 (C-5), 73.3 (C-3), 72.6 (C-3T), 70.8 (C-4T), 70.7, 70.4 (C-2T, C-5T), 69.3 (C-2), 68.6 (C-4), 60.9 (C-6), 50.8 (C-6T), 34.4 (CH_2N), 30.7, 30.4 (CH_2-CH_2). Anal. Calcd for $C_{38}H_{60}N_{10}O_{23}$: C, 43.76; H, 5.99; N, 13.43. Found: C, 43.66; H, 6.10; N, 13.29. HRMS (ESI) m/z [M + Na]$^+$ calcd for $C_{38}H_{61}N_{10}O_{23}Na$ 1025.3906, found 1025.3897.

Compound 18

Yield: 126 mg, 93%; $[\alpha]_D^{20}$ +40.6 (*c* = 0.6, H_2O); R_f = 0.34 (BuOH : EtOH : H_2O 1:1:1); ^{1}H NMR (500 MHz, $CDCl_3$) δ = 7.80 (s, 1 H, H-triazole), 4.89 (d, 1 H, $J_{1,2}$ = 9.2 Hz, H-1), 4.71 (dd, 1 H, $J_{5T,6aT}$ = 2.0, $J_{6aT,6bT}$ = 14.5 Hz, H-6aT), 4.49 (d, 1 H, $J_{1T,2T}$ = 3.9 Hz, H-1T), 4.45 (dd, 1 H, $J_{5T,6aT}$ = 8.0, $J_{6aT,6bT}$ = 14.5 Hz, H-6bT), 4.36 (d, 1 H, $J_{1',2'} \cong$ 7.8 Hz, H-1´), 4.35 (s, 2 H, NH-CH_2), 3.93 (ddd, 1 H, $J_{5T,6aT}$ = 2.2, $J_{5T,6bT}$ = 7.9, $J_{4T,5T}$ = 10.2 Hz, H-5T), 3.72 – 3.55 (m, 9 H, H-3´, H-3, H-3T, H-4, H-5´, H-5, H-6a´, H-6b´, H-6b), 3.45 (dd, 1 H, $J_{1',2'}$ = 7.8, $J_{2',3'}$ = 9.8 Hz, H-2´), 3.36 (dd, 1 H, $J_{1T,2T}$ = 3.9, $J_{2T,3T}$ = 9.9 Hz, H-2T), 3.34 (t, 1 H,$J_{1,2}$ = $J_{2,3}$ =8.8 Hz, H-2), 3.12

(t, 1 H, $J_{3T,4T}$ = $J_{4T,5T}$ = 9.5 Hz, H-4T), 2.59 – 2.47 (m, 4 H, C*H*$_2$-C*H*$_2$); ^{13}C NMR (125 MHz, D_2O) δ =175.9, 174.6 (*C*O), 144.7 (C-4 triazole), 124.7 (C-5 triazole), 102.9 (C-1´), 93.3 (C-1T), 79.1 (C-1), 77.8, 76.3, 75.3, 75.0, 72.6, 72.5, 71.5, 70.9, 70.8 (C-2´, C-2, C-2T, C-3´, C-3, C-3T, C-4, C-5´, C-5), 70.7 (C-4T), 70.4 (C-5T), 68.5 (C-4´), 61.0 (C-6´), 59.9 (C-6), 50.8 (C-6T), 34.4 (NH-*C*H$_2$), 30.8, 30.4 (*C*H$_2$-*C*H$_2$). Anal. Calcd for $C_{50}H_{80}N_{10}O_{33}.2H_2O$: C, 43.35; H, 6.11; N, 10.11. Found: C, 43.04; H, 5.95; N, 9.80. HRMS (ESI): *m/z* $[M\text{+Na}]^+$ calcd for $C_{50}H_{80}N_{10}O_{33}Na$: 1371.4781, found: 1371.4797.

Compound 20

Yield: 52 mg, 84%; $[\alpha]_D^{20}$ +29.1 (*c* = 0.4, H_2O); R_f = 0.63 (BuOH : EtOH : H_2O, 2.5:1:1); ^{1}H NMR (500 MHz, D_2O) δ = 7.94 (H-triazole), 4.74 (dd, 1 H, $J_{5G,6aG}$ = 2.5, $J_{6aG,6bG}$ = 14.6 Hz, H-6aG), 4.64 (d, 1 H, $J_{1G,2G}$ = 3.7 Hz, H-1G), 4.52 (dd, 1 H, $J_{5G,6bG}$ = 2.0, $J_{6aG,6bG}$ = 14.6 Hz, H-6bG), 4.35 (d, 1 H, $J_{1,2}$ = 9.4 Hz, H-1), 4.34 (d, 1 H, $J_{1',2'}$ = 8.5 Hz, H-1´), 4.02, 3.91 (2d, 2 H, *J* = 14.7 Hz, C*H*$_2$S), 3.88−3.83 (m, 3 H, H-6a, H-4´, H-5G), 3.72−3.60 (m, 4 H, H-6b, H-5´, H-6´a, H-6´b), 3.60−3.54 (m, 3 H, H-4, H-3´, H-3G), 3.50−3.42 (m, 4 H, H-3, H-5, H-2´, H-2G), 3.31 (t, 1 H, $J_{1,2}$ = $J_{2,3}$ = 9.4 Hz, H-2), 3.15 (t, 1 H, $J_{3G,4G}$ = $J_{4G,5G}$ = 9.5 Hz, H-4G), 3.05 (s, 3 H, OC*H*$_3$); ^{13}C NMR (125 MHz, $CDCl_3$) δ =144.9 (C-4 triazole), 125.3 (C-3 triazole), 102.9 (C-1´), 99.1 (C-1G), 84.4 (C-1), 78.6, 78.1, 75.8, 75.3, 73.0, 72.5, 71.8, 71.0, 70.9, 70.8, (C-2, C-3, C-4, C-5, C-2´, C-3´, C-5´, C-2G, C-3G, C-4G), 69.8 (C-5G), 68.5 (C-4´), 61.0 (C-6´), 60.2 (C-6), 54.9 (O*C*H$_3$), 50.9 (C-6G), 23.3 (*C*H$_2$S). Anal. Calcd for $C_{22}H_{37}N_3O_{15}S$: C, 42.92; H, 6.06; N, 6.83; S, 5.21. Found: C, 42.56; H, 6.18; N, 6.59; S, 5.23. HRMS (ESI):*m/z* $[M\text{+H}]^+$ calcd for $C_{22}H_{38}N_3O_{15}S$: 616.2018, found: 616.2044.

Compound 22

Yield: 101 mg, 85%; $[\alpha]_D^{20}$ +5.3 (*c* = 0.3, H_2O); R_f = 0.34 (BuOH : EtOH : H_2O, 2.5:1:1); ^{1}H NMR (500 MHz, D_2O) δ = 7.91 (H-triazole), 4.71 (dd, 1 H, $J_{5T,6aT}$ = 2.2, $J_{6aT,6bT}$ = 14.6 Hz, H-6aT), 4.49 (d, 1 H, $J_{1T,2T}$ = 4.0 Hz, H-1T), 4.48 (dd, 1 H, $J_{5T,6bT}$ = 8.5, $J_{6aT,6bT}$ = 14.6 Hz, H-6bT), 4.39 (d, 1 H, $J_{1,2}$ = 9.4 Hz, H-1), 4.37 (d, 1 H, $J_{1',2'}$ = 8.5 Hz, H-1´), 4.04, 3.91 (2d, 2 H, *J* = 14.7

Hz, CH_2S), 3.96 (ddd, 1 H, $J_{5T,6aT}$ = 2.2, $J_{5T,6bT}$ = 8.5, $J_{4T,5T}$ = 10.3 Hz, H-5T), 3.84−3.80 (m, 2 H, H-6a, H-4´), 3.74−3.51 (m, 9 H, H-3, H-4, H-5, H-6b, H-3´, H-5´, H-6´a, H-6´b, H-3T), 3.48−3.43 (m, 3 H, H-5, H-2´, H-2T), 3.34 (t, 1 H, $J_{1,2}$ = $J_{2,3}$= 9.3 Hz, H-2), 3.16 (t, 1 H, $J_{3T,4T}$ = $J_{4T,5T}$ = 9.5 Hz, H-4T); ^{13}C NMR (125 MHz, $CDCl_3$) δ =145.0 (C-4 triazole), 125.2 (C-3 triazole), 102.9 (C-1´), 93.1 (C-1T), 84.4 (C-1), 78.6, 78.1, 75.7, 75.3, 72.6, 72.5, 71.8, 71.0, 70.6, 70.5, 70.4, (C-2, C-3, C-4, C-5, C-2´, C-3´, C-5´, C-2T, C-3T, C-4T, C-5T), 68.5 (C-4´), 61.0 (C-6´), 60.2 (C-6), 51.0 (C-6T), 23.2 (CH$_2$S). Anal. calcd for $C_{42}H_{68}N_6O_{29}S_2$: C, 42.56; H, 5.78; N, 7.09; S, 5.41. Found: C, 42.31; H, 5.56; N, 6.72; S, 5.33. HRMS (ESI): *m/z* [*M*+Na]$^+$ calcd for $C_{42}H_{68}N_6NaO_{29}S_2$: 1207.3364, found: 1207.3389.

References

1. Veh, R. W.; Michalski, J.; Corfield, A. P.; Sander-Wewer, M.; Gies, D.; Schauer, R. J. *Chromatogr.*, **1981**, *212*, 313-322.

2. Cagnoni, A. J.; Varela, O.; Gouin, S. G.; Kovensky, J.; Uhrig, M. L. *J. Org. Chem.* **2011**, *76*, 3064-3077.

3. Cagnoni, A. J.; Varela, O.; Uhrig, M. L.; Kovensky, J. *Eur. J. Org. Chem.* **2013**, 972-983.

4. Murphy, P. V.; Bradley, H.; Tosin, M.; Pitt, N.; Fitzpatrick, G. M.; Glass, W. K. *J. Org. Chem.* **2003**, *68*, 5692-5704.

5. Cros, C. D.; Toth, I.; Blanchfield, J. T. *Bioorg. Med. Chem. Lett.* **2014**, *24*, 1373-1375.

6. Fujihira, T.; Chida, M.; Kamijo, H.; Takido, T.; Seno, M. J. *J. Carbohydr. Chem.* **2002**, *21*, 287-292.

7. Cagnoni, A. J.; Varela, O.; Gouin, S. G.; Kovensky, J.; Uhrig, M. L. *Org. Biomol. Chem.* **2013**, *11*, 5500-5511.
