# Supplementary material for: Synthesis of divalent ligands of β-thio- and β-N-galactopyranosides and related lactosides and their evaluation as substrates and inhibitors of Trypanosoma cruzi trans-sialidase
Source: Beilstein J Org Chem. 2014 Dec 19;10:3073–86. doi: 10.3762/bjoc.10.324 (PMC4311708; doi:10.3762/bjoc.10.324)
Supplement: File 2 — Copies of 1H and 13C NMR spectra of compounds 2, 3, 5, 6, 8, 10–13, 15–22 and 25. [file Beilstein_J_Org_Chem-10-3073-s002.pdf]

Supporting Information File 2

for

Synthesis of divalent ligands of β-thio- and β-*N*-galactopyranosides and related lactosides and their evaluation as substrates and inhibitors of *Trypanosoma cruzi* trans-sialidase

María Emilia Cano[§,1], Rosalía Agusti[§,1], Alejandro J. Cagnoni[1], María Florencia Tesoriero[1], José Kovensky[2], María Laura Uhrig[*,1], Rosa M. de Lederkremer[*,1]

Address: [1]CIHIDECAR-CONICET, Departamento de Química Orgánica, Facultad de Ciencias Exactas y Naturales, Universidad de Buenos Aires, Pabellón 2, Ciudad Universitaria, 1428 Buenos Aires (Argentina), Fax: (+) 541145763346 and [2] Laboratoire de Glycochimie, des Antimicrobiens et des Agroressources (LG2A)-CNRS FRE 3517, Université de Picardie Jules Verne, 33 rue Saint Leu, 80039 Amiens Cedex, France.

E-mail: María Laura Uhrig - mluhrig@qo.fcen.uba.ar; Rosa M. de Lederkremer - lederk@qo.fcen.uba.ar

*Corresponding author

[§]Contributed equally to this work.

Copies of ^{1}H and ^{13}C NMR spectra of compounds 2, 3, 5, 6, 8, 10–13, 15–22 and 25

Compound **2**

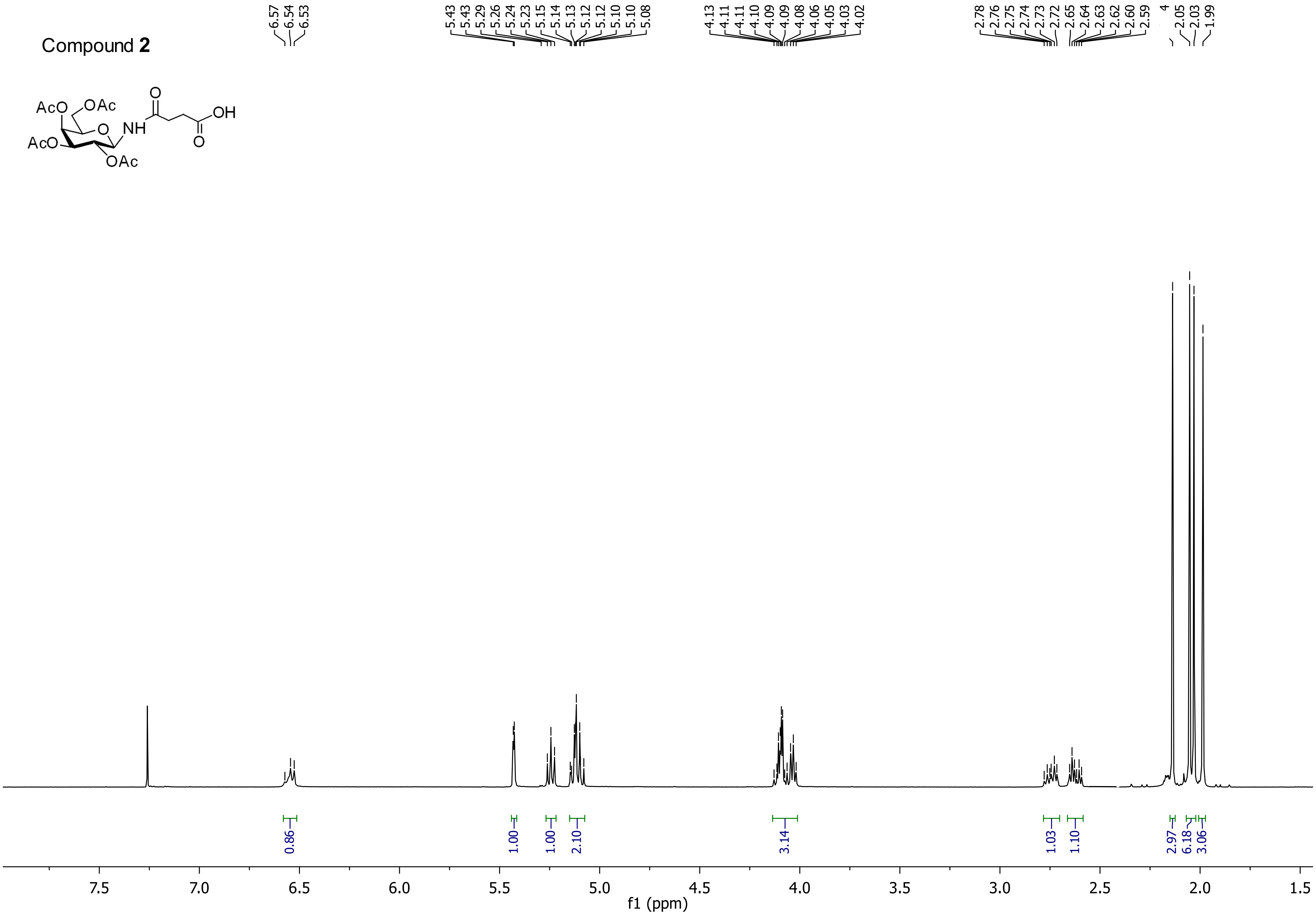

AcO
OAc
O
NH
O
OH
O
AcO
OAc
6.57
6.54
6.53
5.43
5.43
5.29
5.26
5.24
5.23
5.15
5.14
5.13
5.12
5.12
5.10
5.10
5.08
4.13
4.11
4.11
4.10
4.09
4.09
4.08
4.06
4.05
4.03
4.02
2.78
2.76
2.75
2.74
2.73
2.72
2.65
2.64
2.63
2.62
2.60
2.59
2.05
2.03
1.99
0.86
1.00
1.00
2.10
3.14
1.03
1.10
2.97
6.18
3.06
7.5
7.0
6.5
6.0
5.5
5.0
4.5
4.0
3.5
3.0
2.5
2.0
1.5
f1 (ppm)

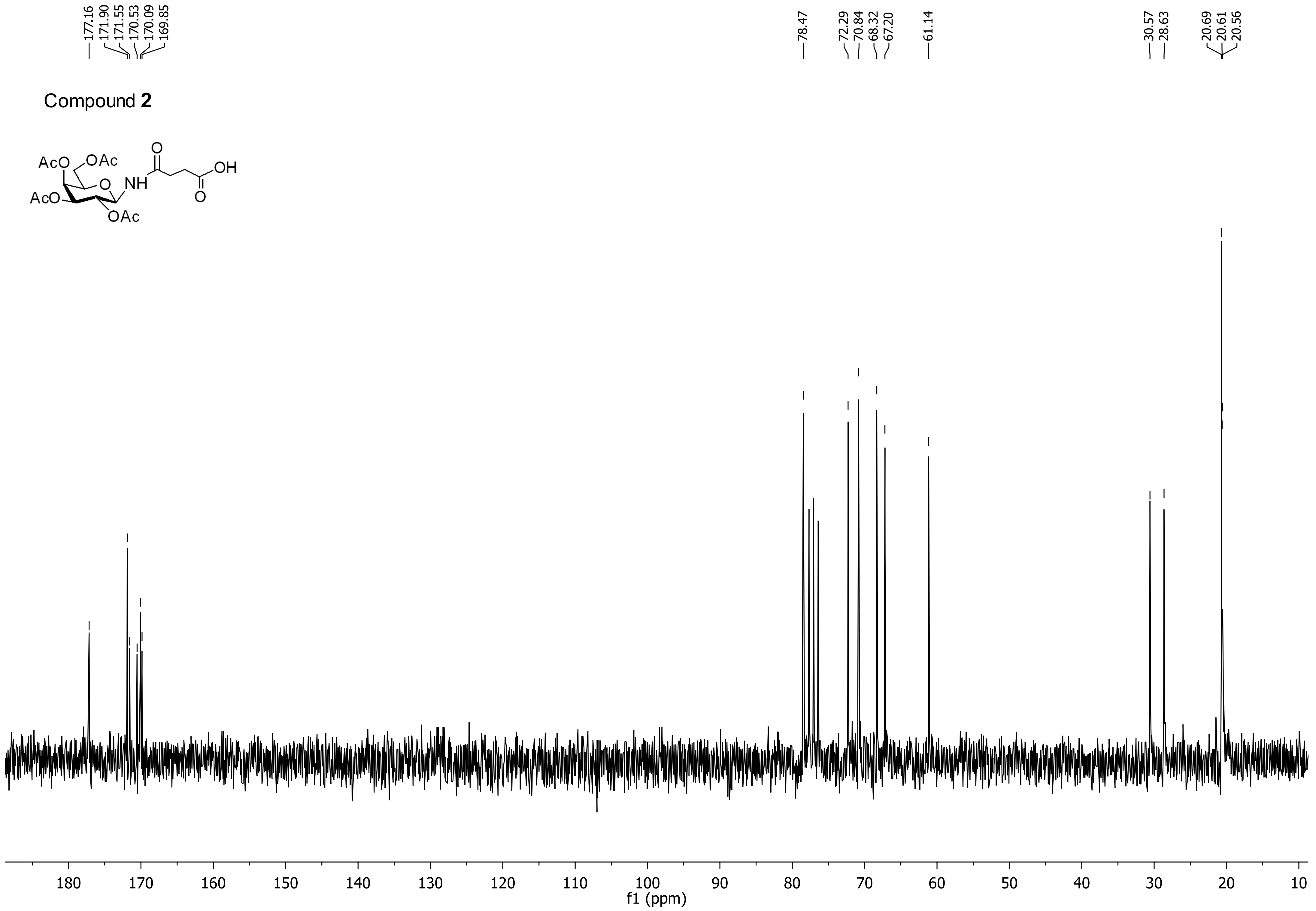

177.16
171.90
171.55
170.53
170.09
169.85
78.47
72.29
70.84
68.32
67.20
61.14
30.57
28.63
20.69
20.61
20.56
Compound **2**
AcO
OAc
O
NH
O
OH
AcO
O
OAc
180
170
160
150
140
130
120
110
100
90
80
70
60
50
40
30
20
10
f1 (ppm)

Compound **3**

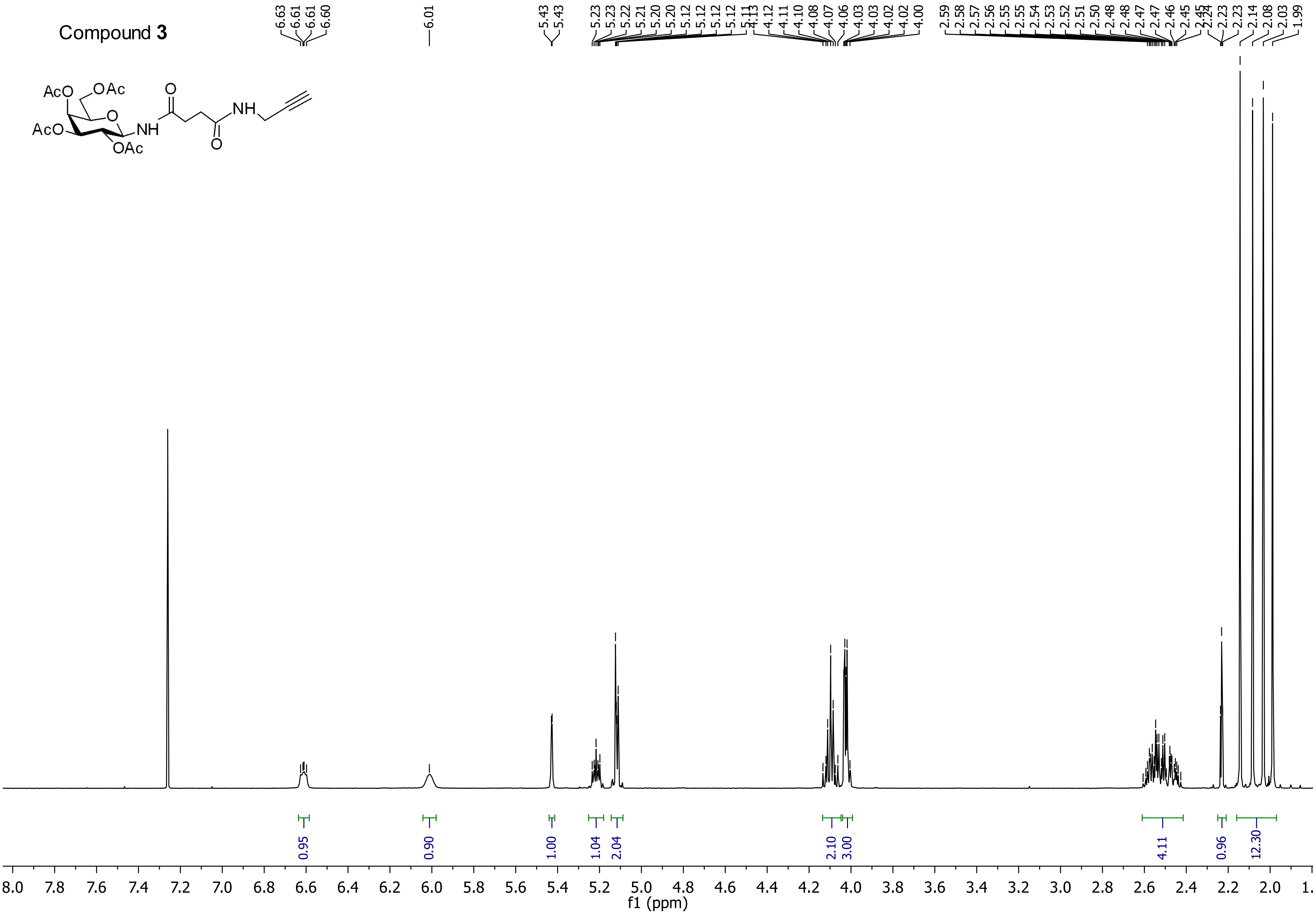

AcO
OAc
O
O
NH
NH
AcO
OAc
O
6.63
6.61
6.61
6.60
6.01
5.43
5.43
5.23
5.23
5.22
5.21
5.20
5.20
5.12
5.12
5.12
5.11
4.13
4.12
4.11
4.10
4.08
4.07
4.06
4.03
4.03
4.02
4.02
4.00
2.59
2.58
2.57
2.56
2.55
2.55
2.54
2.53
2.52
2.51
2.50
2.48
2.48
2.47
2.47
2.46
2.45
2.23
2.23
2.14
2.08
2.03
1.99
0.95
0.90
1.00
1.04
2.04
2.10
3.00
4.11
0.96
12.30
8.0 7.8 7.6 7.4 7.2 7.0 6.8 6.6 6.4 6.2 6.0 5.8 5.6 5.4 5.2 5.0 4.8 4.6 4.4 4.2 4.0 3.8 3.6 3.4 3.2 3.0 2.8 2.6 2.4 2.2 2.0 1.
f1 (ppm)

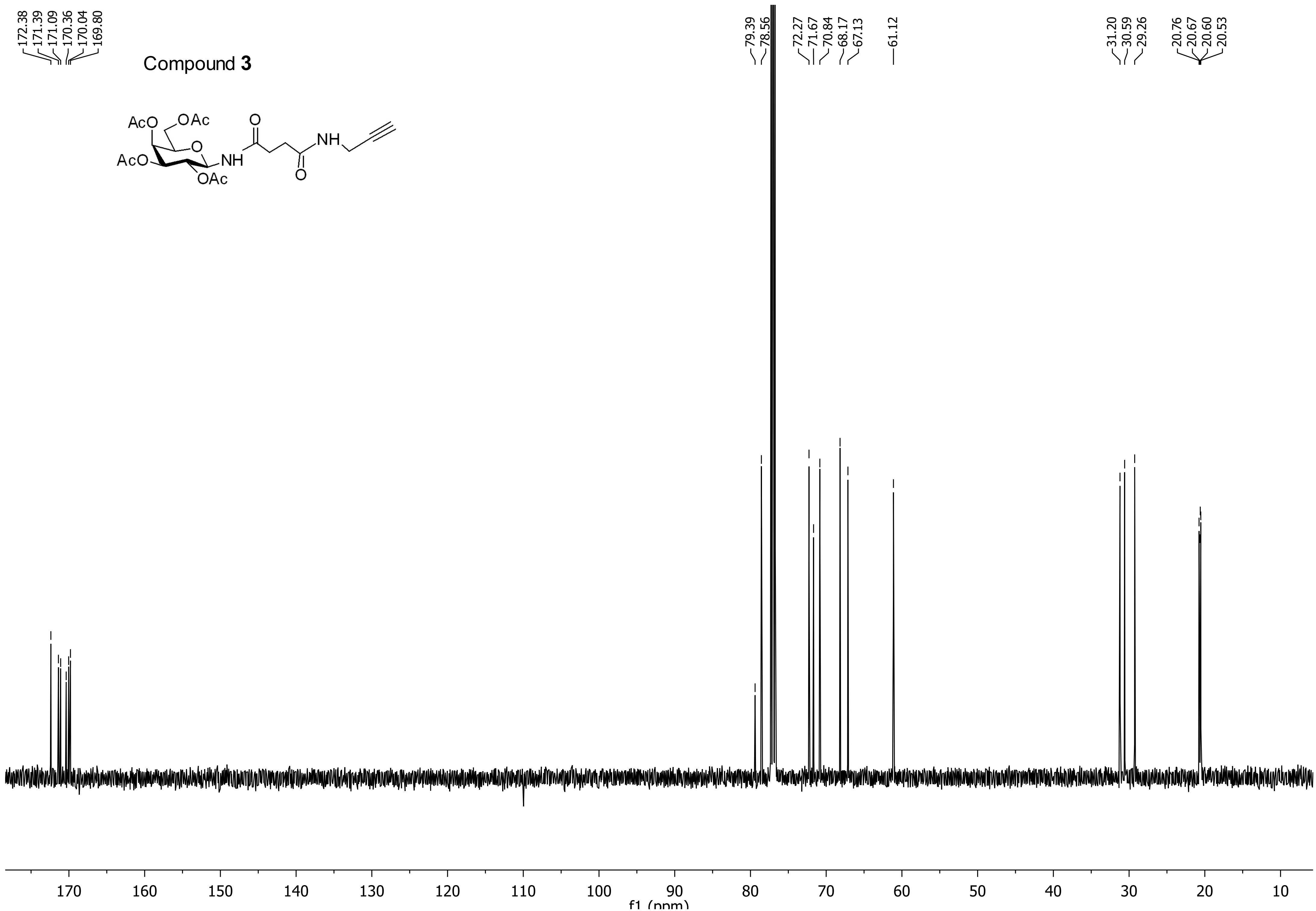
172.38
171.39
171.09
170.36
170.04
169.80
AcO
OAc
O
NH
OAc
AcO
O
NH
O
79.39
78.56
72.27
71.67
70.84
68.17
67.13
61.12
31.20
30.59
29.26
20.76
20.67
20.60
20.53
170
160
150
140
130
120
110
100
90
80
70
60
50
40
30
20
10
f1 (ppm)

Compound **3**

Compound **5**

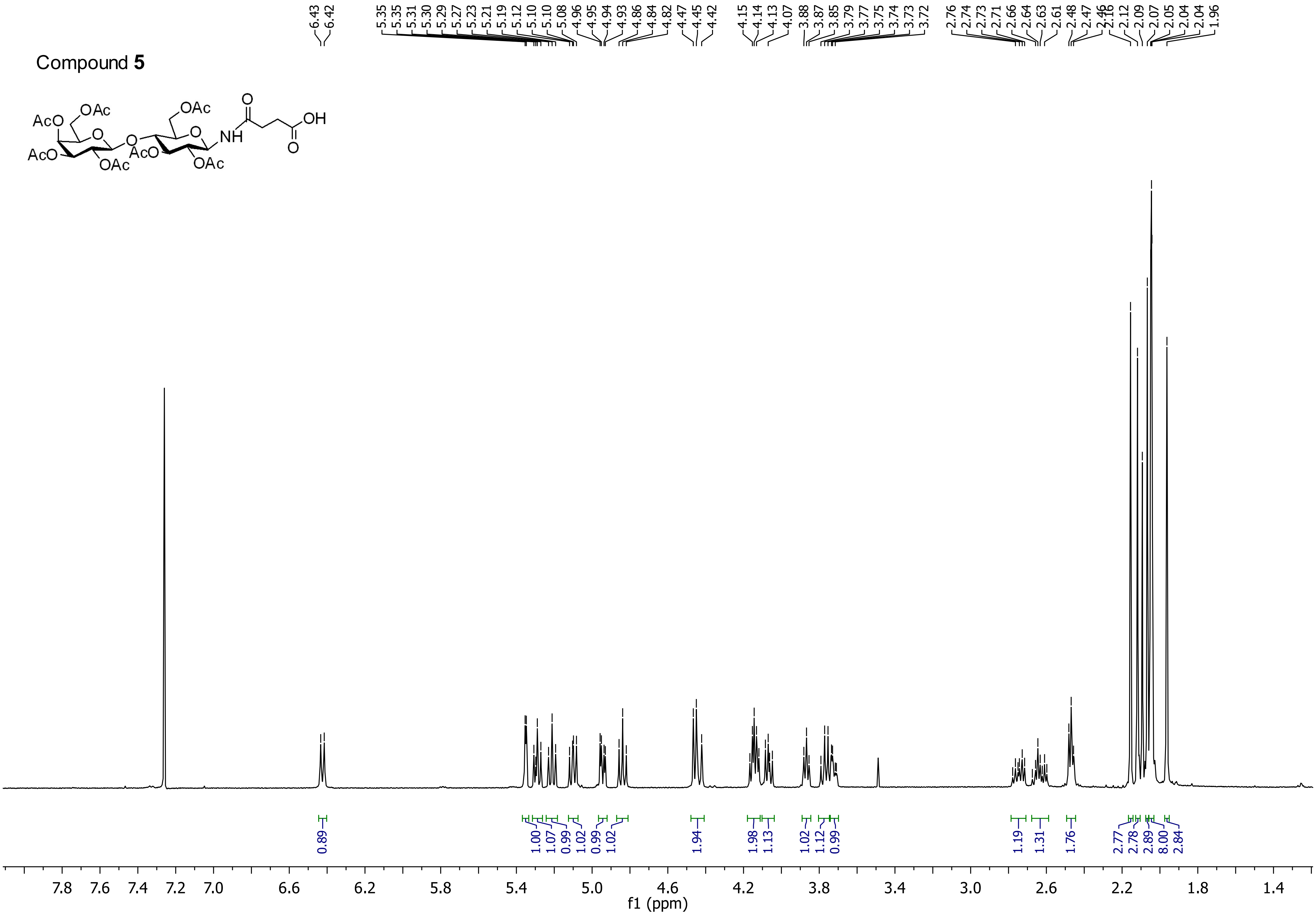

AcO
OAc
OAc
AcO
OAc
AcO
OAc
OAc
O
NH
OH
O
6.43
6.42
5.35
5.35
5.31
5.30
5.29
5.27
5.23
5.21
5.19
5.12
5.10
5.10
5.08
4.96
4.95
4.94
4.93
4.86
4.84
4.82
4.47
4.45
4.42
4.15
4.14
4.13
4.07
3.88
3.87
3.85
3.79
3.77
3.75
3.74
3.73
3.72
2.76
2.74
2.73
2.71
2.66
2.64
2.63
2.61
2.48
2.47
2.46
2.16
2.12
2.09
2.07
2.05
2.04
2.04
1.96
0.89
1.00
1.07
0.99
1.02
0.99
1.02
1.94
1.98
1.13
1.02
1.12
0.99
1.19
1.31
1.76
2.77
2.78
2.89
8.00
2.84
7.8
7.6
7.4
7.2
7.0
6.6
6.2
5.8
5.4
5.0
4.6
4.2
3.8
3.4
3.0
2.6
2.2
1.8
1.4
f1 (ppm)

Compound **5**

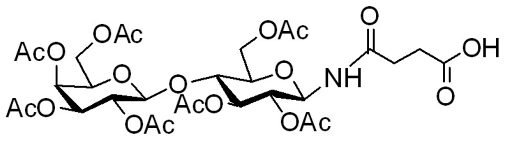

AcO
OAc
AcO
OAc
OAc
AcO
OAc
O
NH
O
OH
O

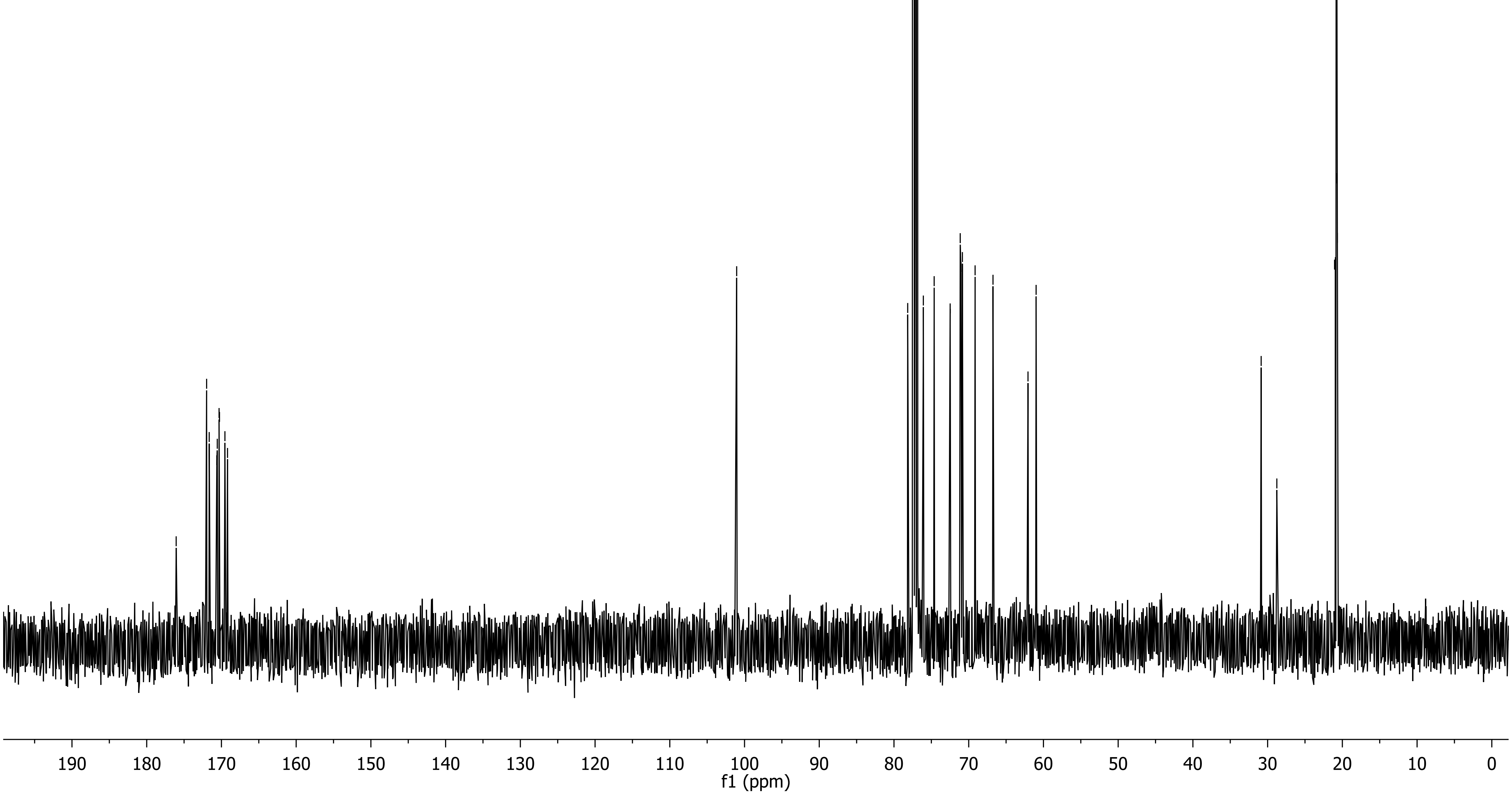

176.06
171.98
171.63
170.62
170.56
170.32
170.26
169.53
169.19
101.05
78.16
76.07
74.62
72.48
71.14
71.03
70.85
69.15
66.75
62.07
60.98
30.86
28.77
21.04
20.93
20.81
20.78
20.67
190
180
170
160
150
140
130
120
110
100
90
80
70
60
50
40
30
20
10
0
f1 (ppm)

Compound **6**

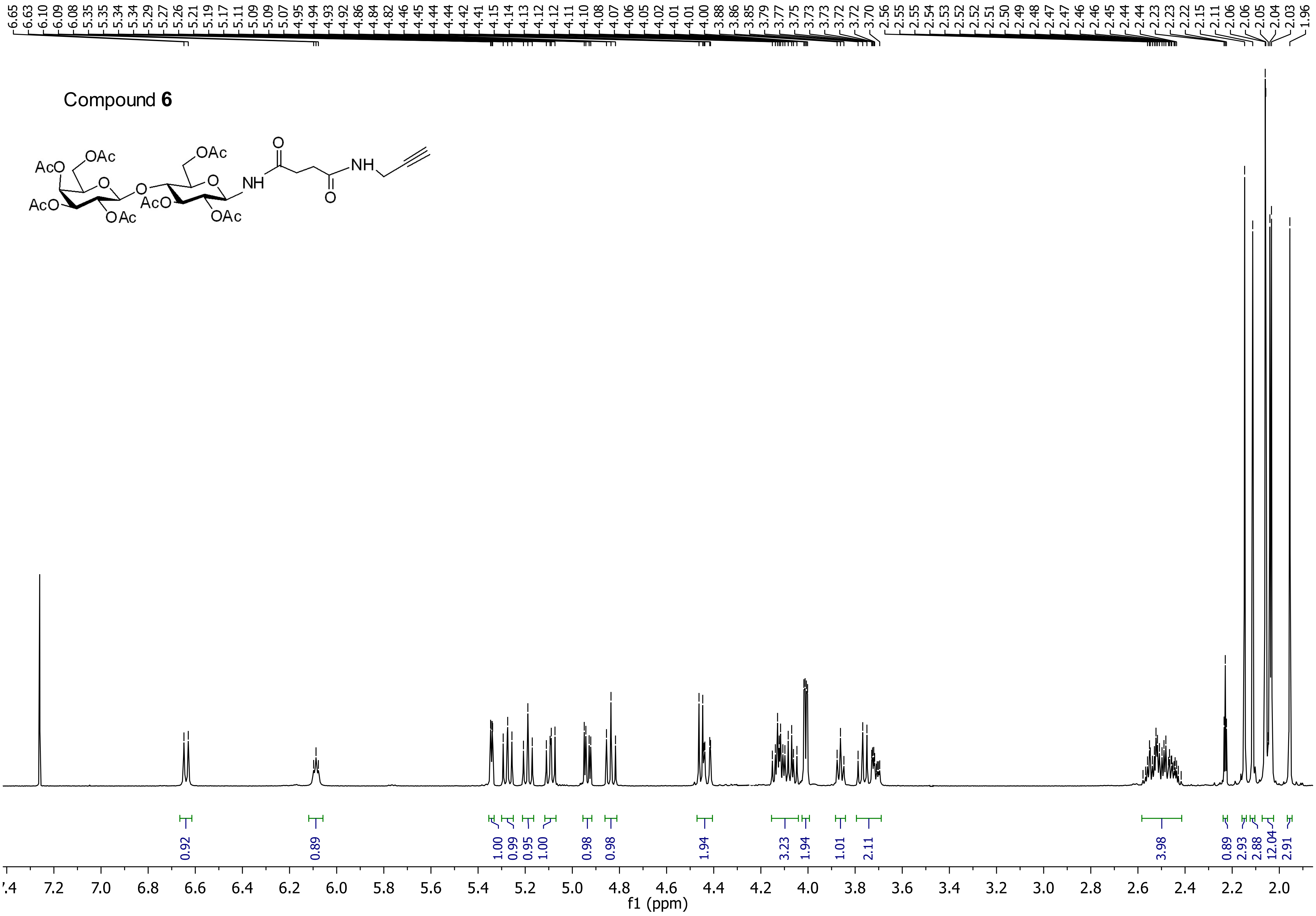

Compound **6**

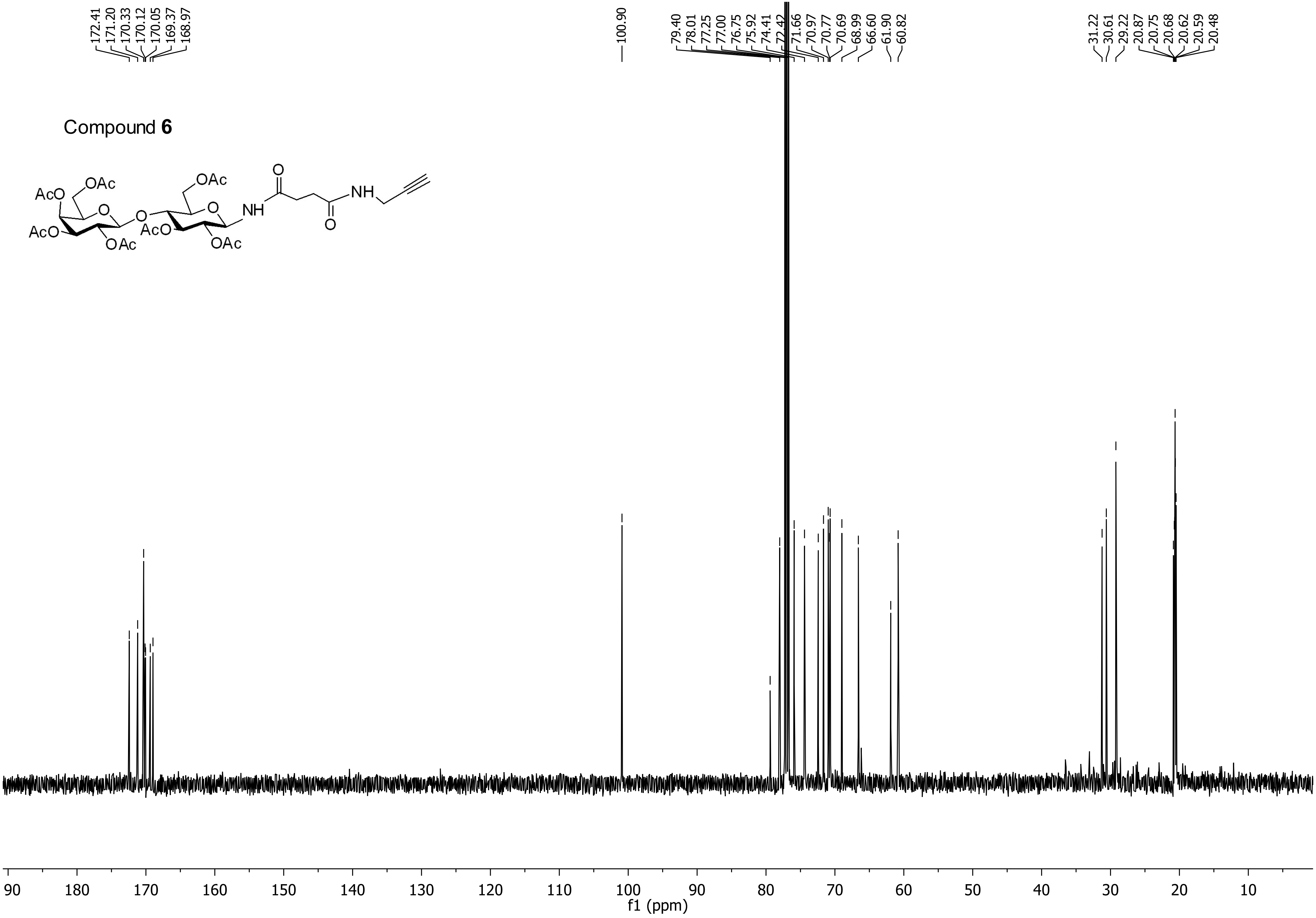

172.41
171.20
170.33
170.12
170.05
169.37
168.97
100.90
79.40
78.01
77.25
77.00
76.75
75.92
74.41
72.42
71.66
70.97
70.77
70.69
68.99
66.60
61.90
60.82
31.22
30.61
29.22
20.87
20.75
20.68
20.62
20.59
20.48
AcO
OAc
NH
O
90 180 170 160 150 140 130 120 110 100 90 80 70 60 50 40 30 20 10
f1 (ppm)

Compound **8**

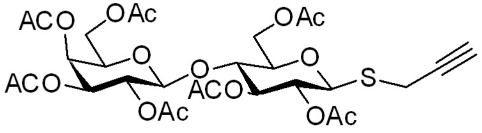
ACO
OAc
O
ACO
OAc
O
ACO
OAc
O
ACO
OAc
S

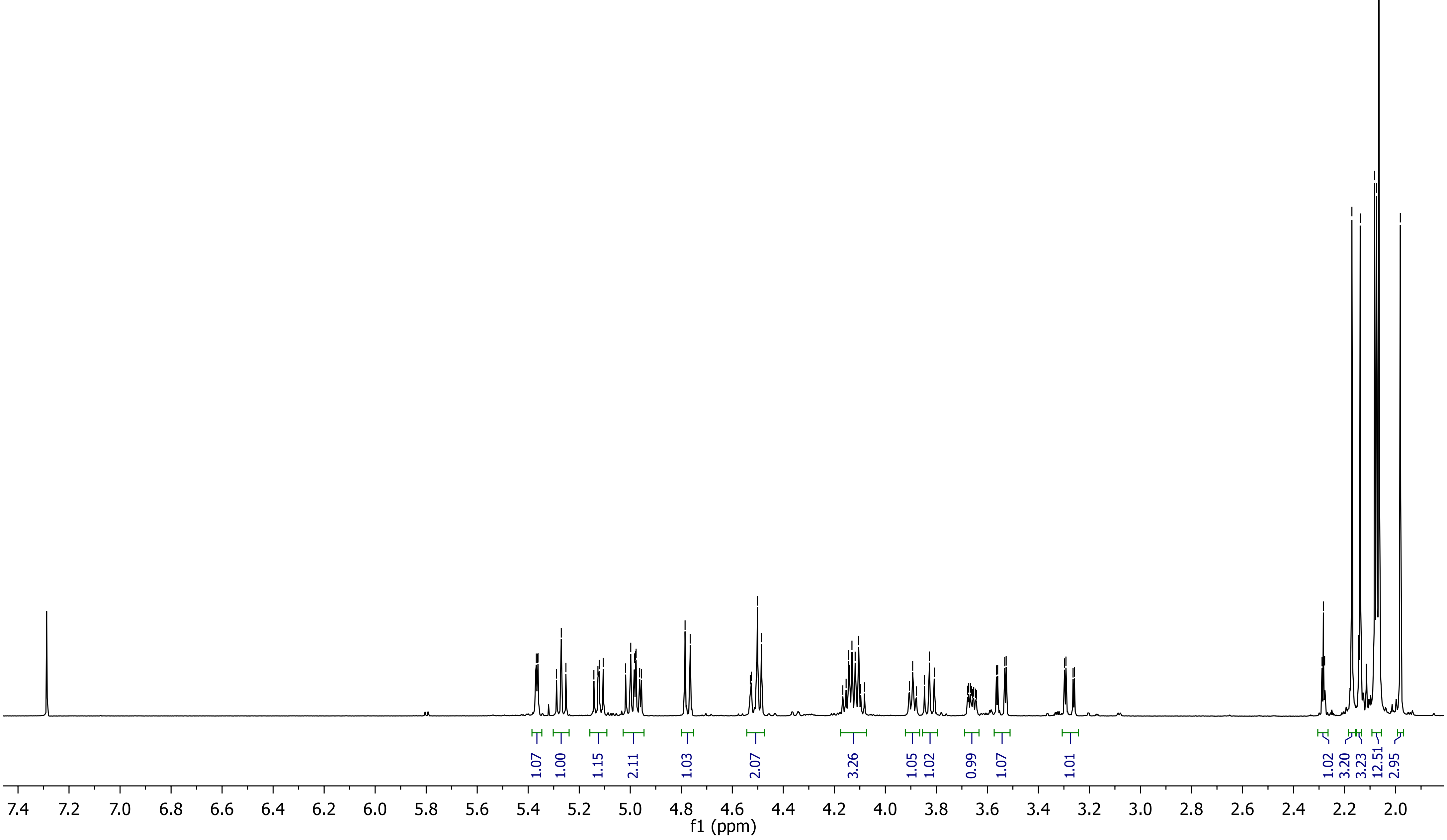
5.37
5.36
5.36
5.29
5.27
5.25
5.14
5.13
5.12
5.11
5.02
5.00
4.98
4.98
4.98
4.96
4.96
4.79
4.76
4.53
4.53
4.51
4.50
4.49
4.14
4.14
4.13
4.12
4.10
3.89
3.83
3.81
3.67
3.56
3.56
3.53
3.29
3.26
3.26
2.29
2.28
2.28
2.17
2.14
2.08
2.07
1.98
1.07
1.00
1.15
2.11
1.03
2.07
3.26
1.05
1.02
0.99
1.07
1.01
1.02
3.20
3.23
12.51
2.95
f1 (ppm)

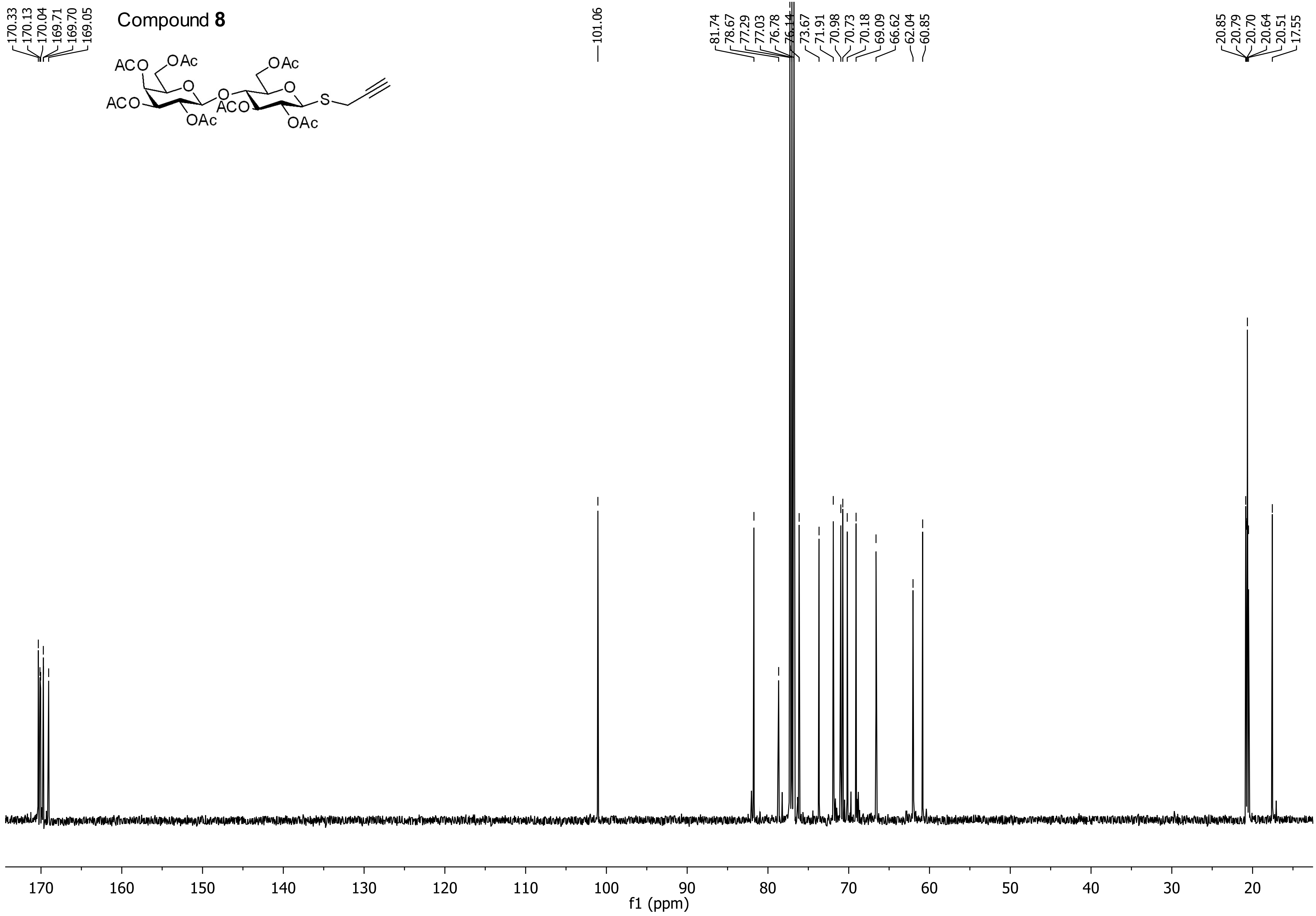
Compound 8
170.33
170.13
170.04
169.71
169.70
169.05
101.06
81.74
78.67
77.29
77.03
76.78
76.14
73.67
71.91
70.98
70.73
70.18
69.09
66.62
62.04
60.85
20.85
20.79
20.70
20.64
20.51
17.55
f1 (ppm)

Compound **10**

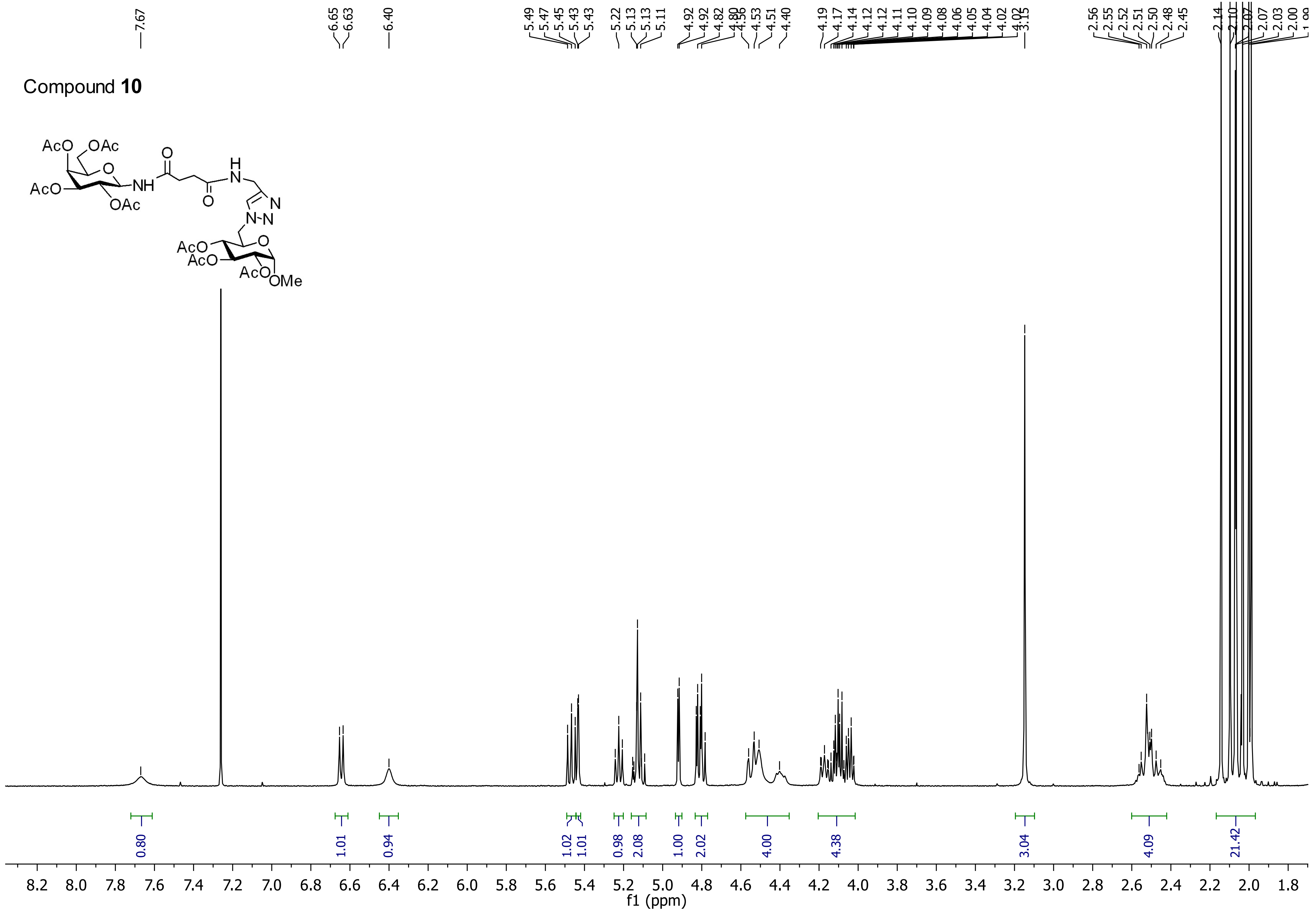

AcO
OAc
O
NH
O
H
N
O
N
N-N
AcO
AcO
AcO
AcO
O
OMe
7.67
6.65
6.63
6.40
5.49
5.47
5.45
5.43
5.43
5.22
5.13
5.13
5.11
4.92
4.92
4.82
4.80
4.56
4.53
4.51
4.40
4.19
4.17
4.14
4.12
4.12
4.11
4.10
4.09
4.08
4.06
4.05
4.04
4.02
4.02
3.15
2.56
2.55
2.52
2.51
2.50
2.48
2.45
2.14
2.10
2.07
2.07
2.03
2.00
0.80
1.01
0.94
1.02
1.01
0.98
2.08
1.00
2.02
4.00
4.38
3.04
4.09
21.42
f1 (ppm)

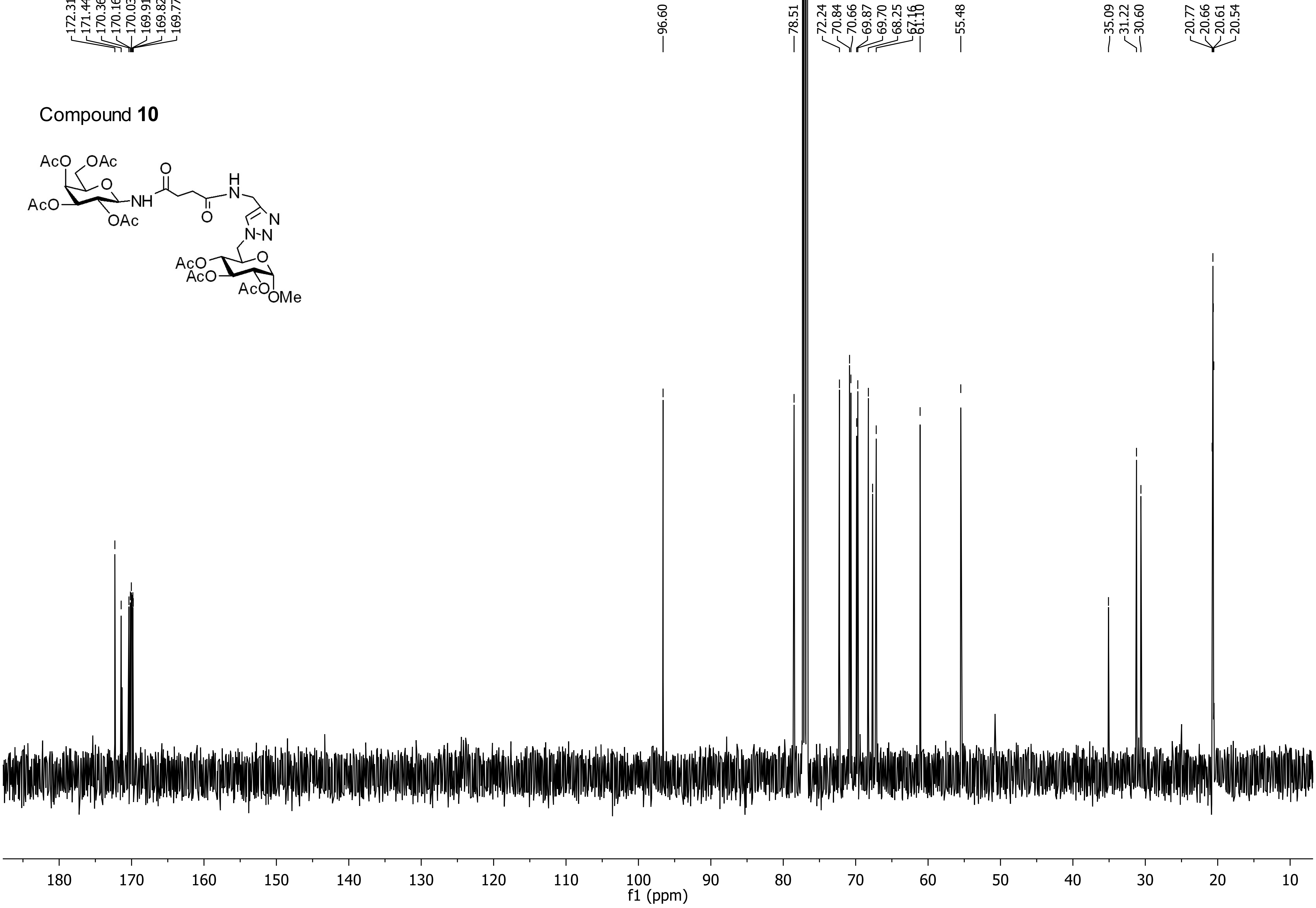
172.31
171.44
170.36
170.16
170.03
169.91
169.82
169.77
96.60
78.51
72.24
70.84
70.66
69.87
69.70
68.25
67.16
61.10
55.48
35.09
31.22
30.60
20.77
20.66
20.61
20.54
Compound **10**
AcO
OAc
O
NH
O
H
N
AcO
OAc
O
N
N-N
AcO
AcO
O
AcO
OMe
180 170 160 150 140 130 120 110 100 90 80 70 60 50 40 30 20 10
f1 (ppm)

Compound **11**

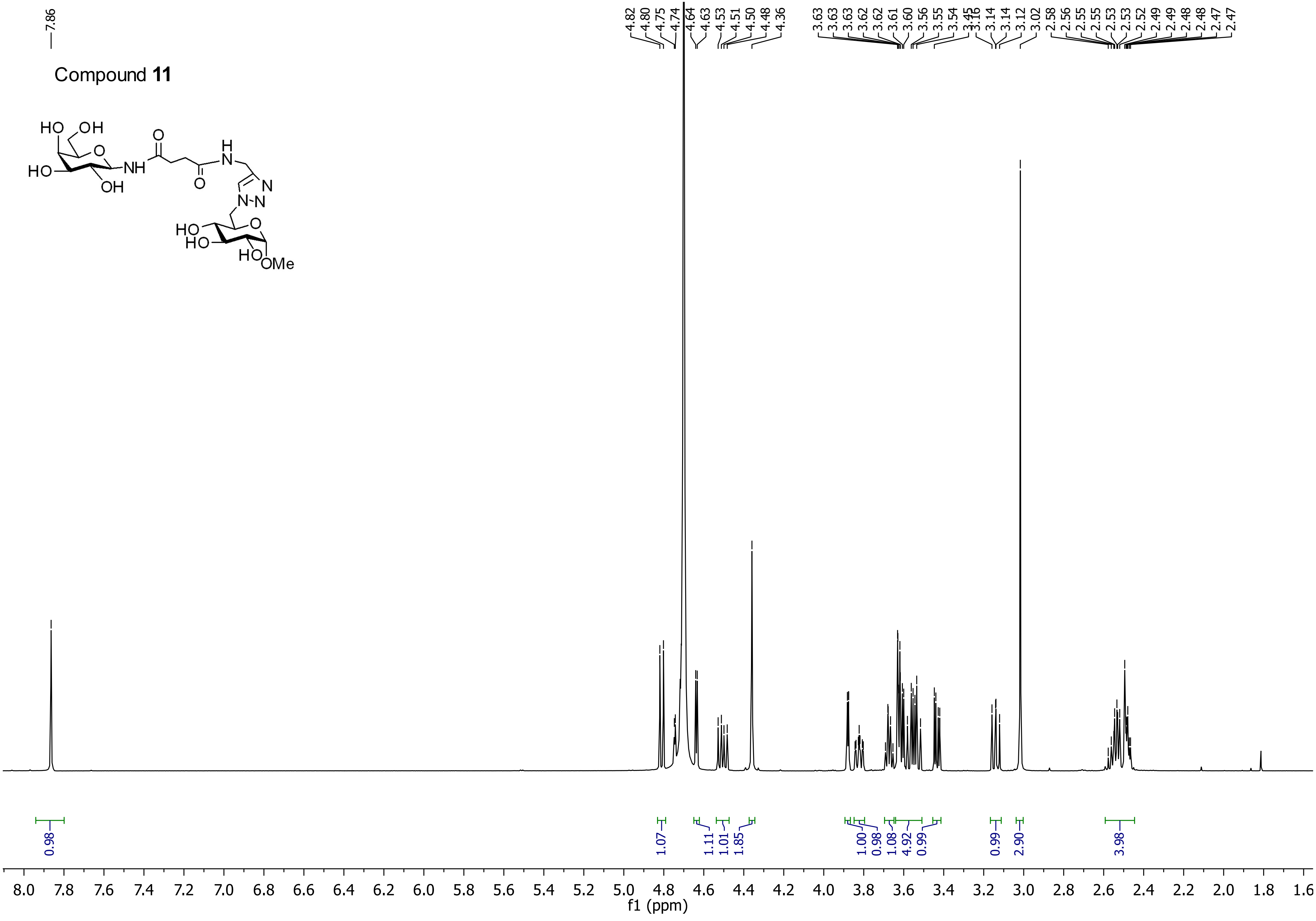

7.86
4.82
4.80
4.75
4.74
4.64
4.63
4.53
4.51
4.50
4.48
4.36
3.63
3.63
3.63
3.62
3.62
3.61
3.60
3.56
3.55
3.54
3.45
3.16
3.14
3.14
3.12
3.02
2.58
2.56
2.55
2.55
2.53
2.53
2.52
2.49
2.49
2.48
2.48
2.47
2.47
HO
OH
O
NH
HO
OH
O
H
N
O
N
N-N
HO
HO
HO
OMe
0.98
1.07
1.11
1.01
1.85
1.00
0.98
1.08
4.92
0.99
0.99
2.90
3.98
8.0 7.8 7.6 7.4 7.2 7.0 6.8 6.6 6.4 6.2 6.0 5.8 5.6 5.4 5.2 5.0 4.8 4.6 4.4 4.2 4.0 3.8 3.6 3.4 3.2 3.0 2.8 2.6 2.4 2.2 2.0 1.8 1.6
f1 (ppm)

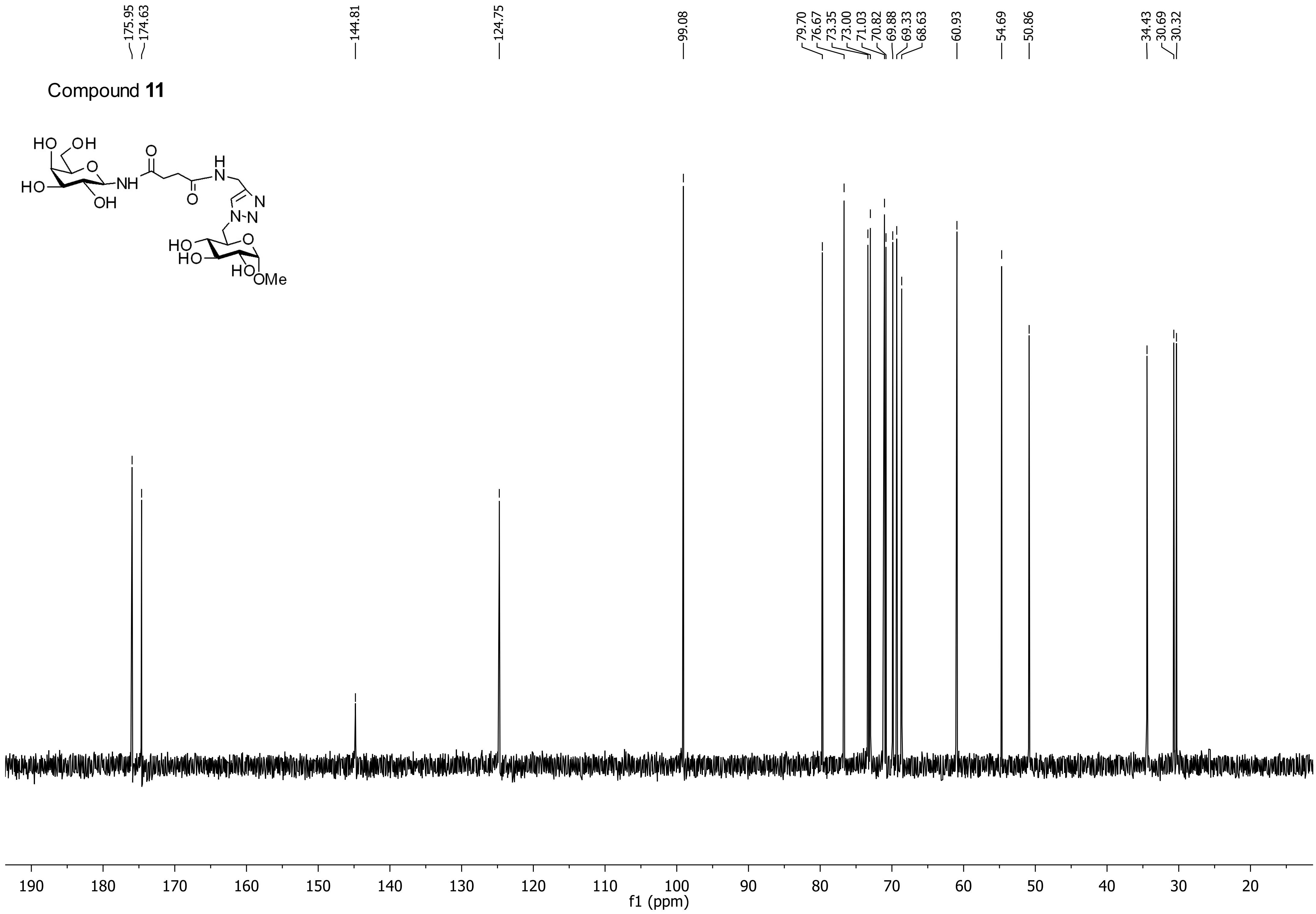

175.95
174.63
144.81
124.75
99.08
79.70
76.67
73.35
73.00
71.03
70.82
69.88
69.33
68.63
60.93
54.69
50.86
34.43
30.69
30.32
Compound 11
HO
OH
O
NH
HO
OH
O
H
N
O
N
N-N
HO
O
HO
HO
OMe
190
180
170
160
150
140
130
120
110
100
90
80
70
60
50
40
30
20
f1 (ppm)

Compound **12**

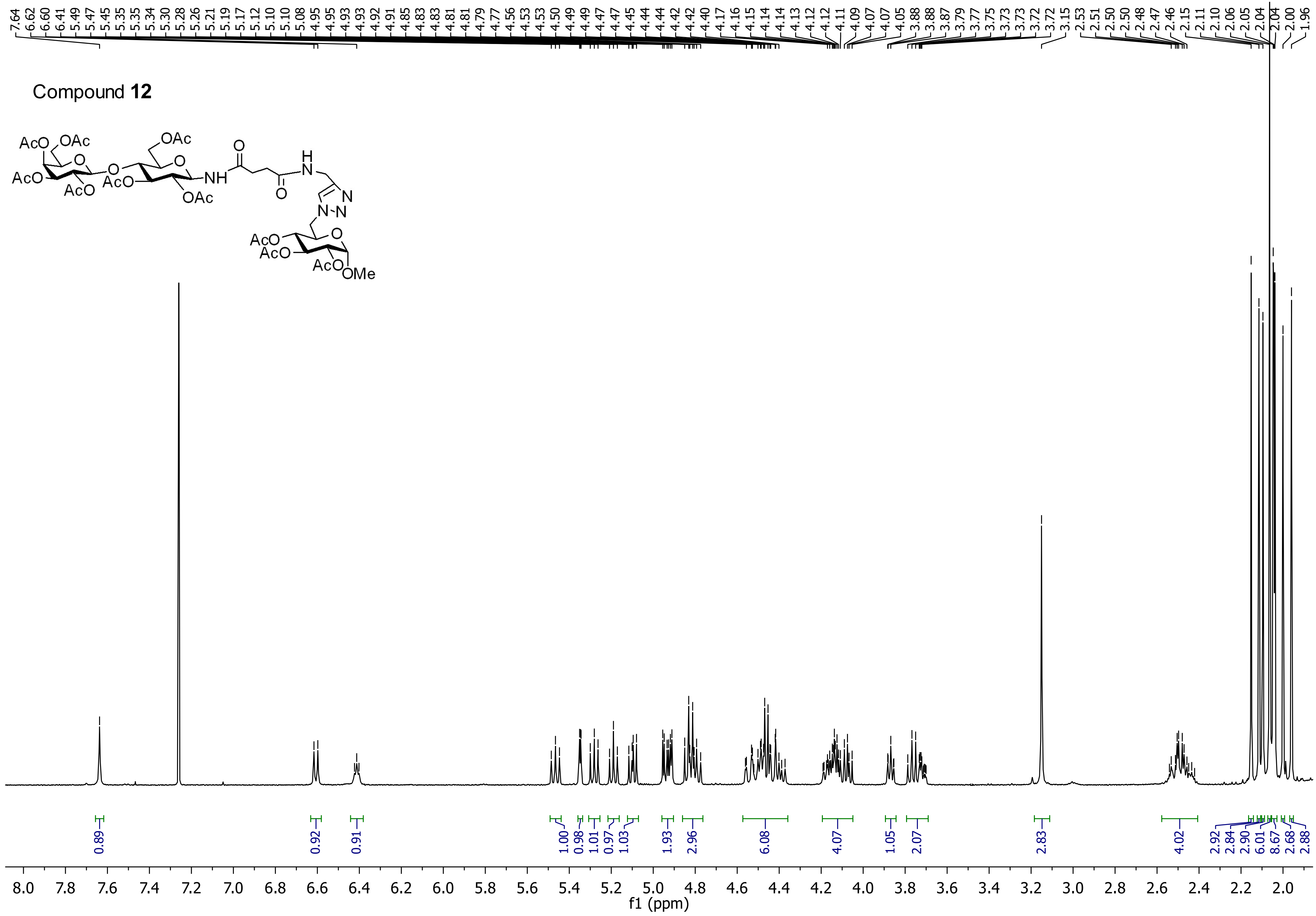

Compound **12**

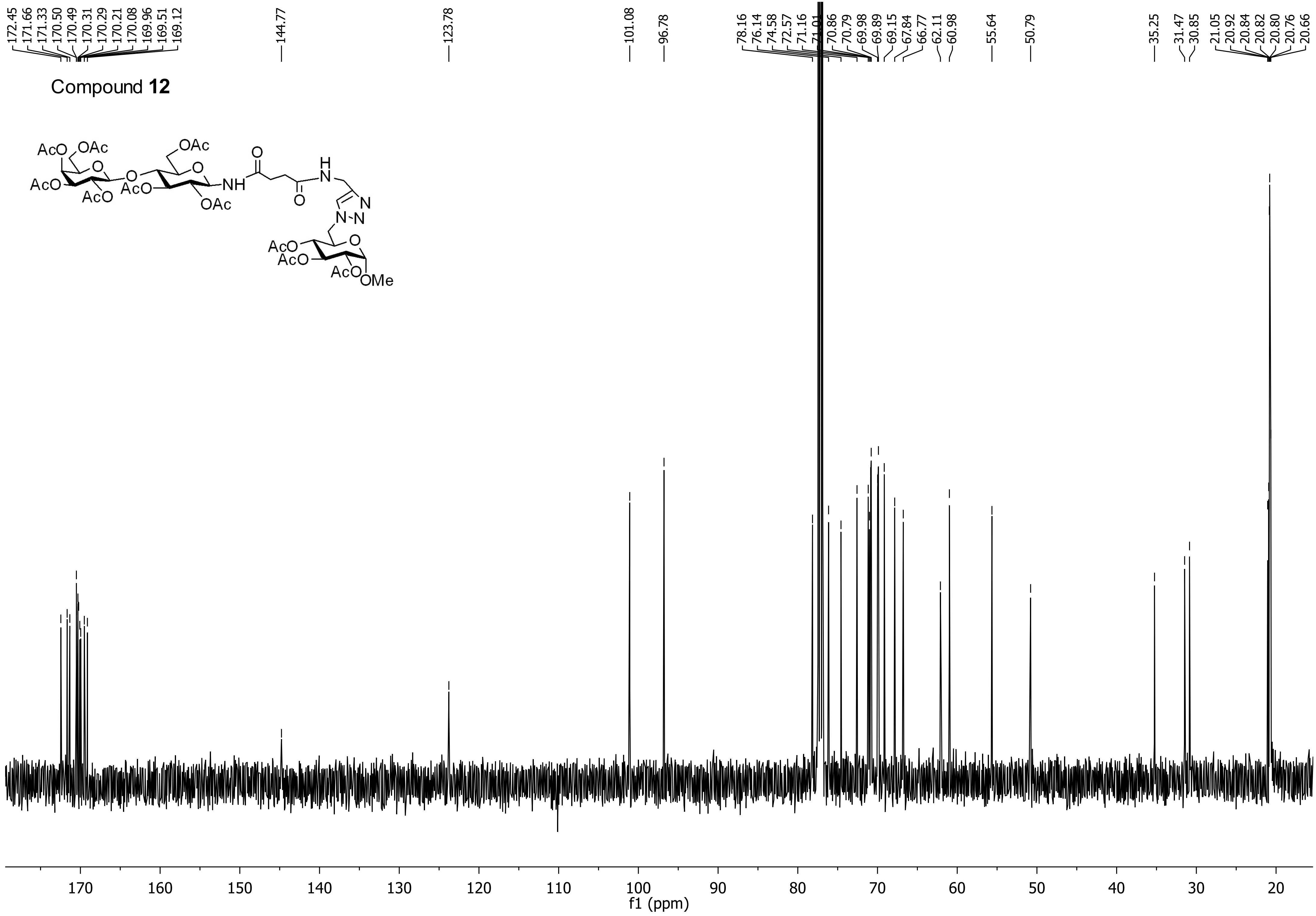
172.45
171.66
171.33
170.50
170.49
170.31
170.29
170.21
170.08
169.96
169.51
169.12
144.77
123.78
101.08
96.78
78.16
76.14
74.58
72.57
71.16
70.86
70.79
69.98
69.89
69.15
67.84
66.77
62.11
60.98
55.64
50.79
35.25
31.47
30.85
21.05
20.92
20.84
20.82
20.80
20.76
20.66
AcO
OAc
NH
OMe
170
160
150
140
130
120
110
100
90
80
70
60
50
40
30
20
f1 (ppm)

Compound **13**

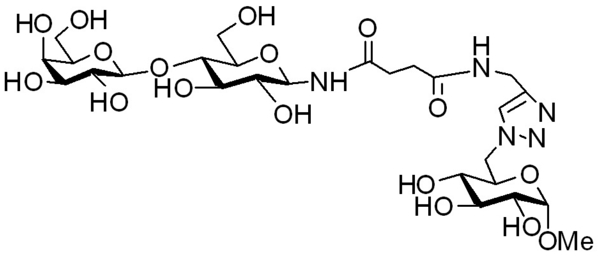

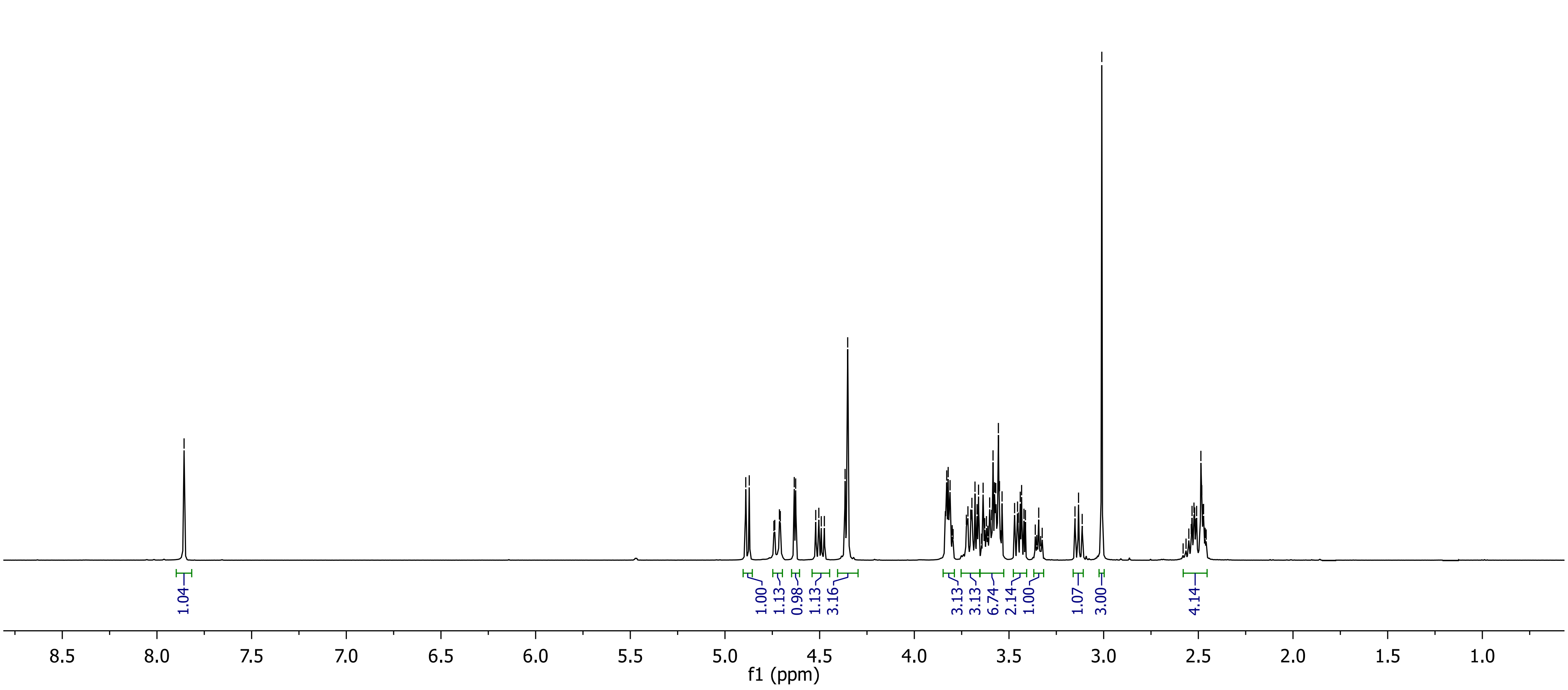

7.86
4.89 4.87 4.74 4.74 4.71 4.71 4.63 4.63 4.52 4.50 4.49 4.48 4.37 4.35
3.83 3.82 3.81 3.68 3.66 3.64 3.58 3.58 3.57 3.56 3.55 3.43 3.01 2.58 2.57 2.55 2.54 2.53 2.52 2.52 2.51 2.49 2.48 2.47 2.47 2.46 2.46
1.04
1.00 1.13 0.98 1.13 3.16
3.13 3.13 6.74 2.14 1.00 1.07 3.00 4.14
8.5 8.0 7.5 7.0 6.5 6.0 5.5 5.0 4.5 4.0 3.5 3.0 2.5 2.0 1.5 1.0
f1 (ppm)

Compound **13**

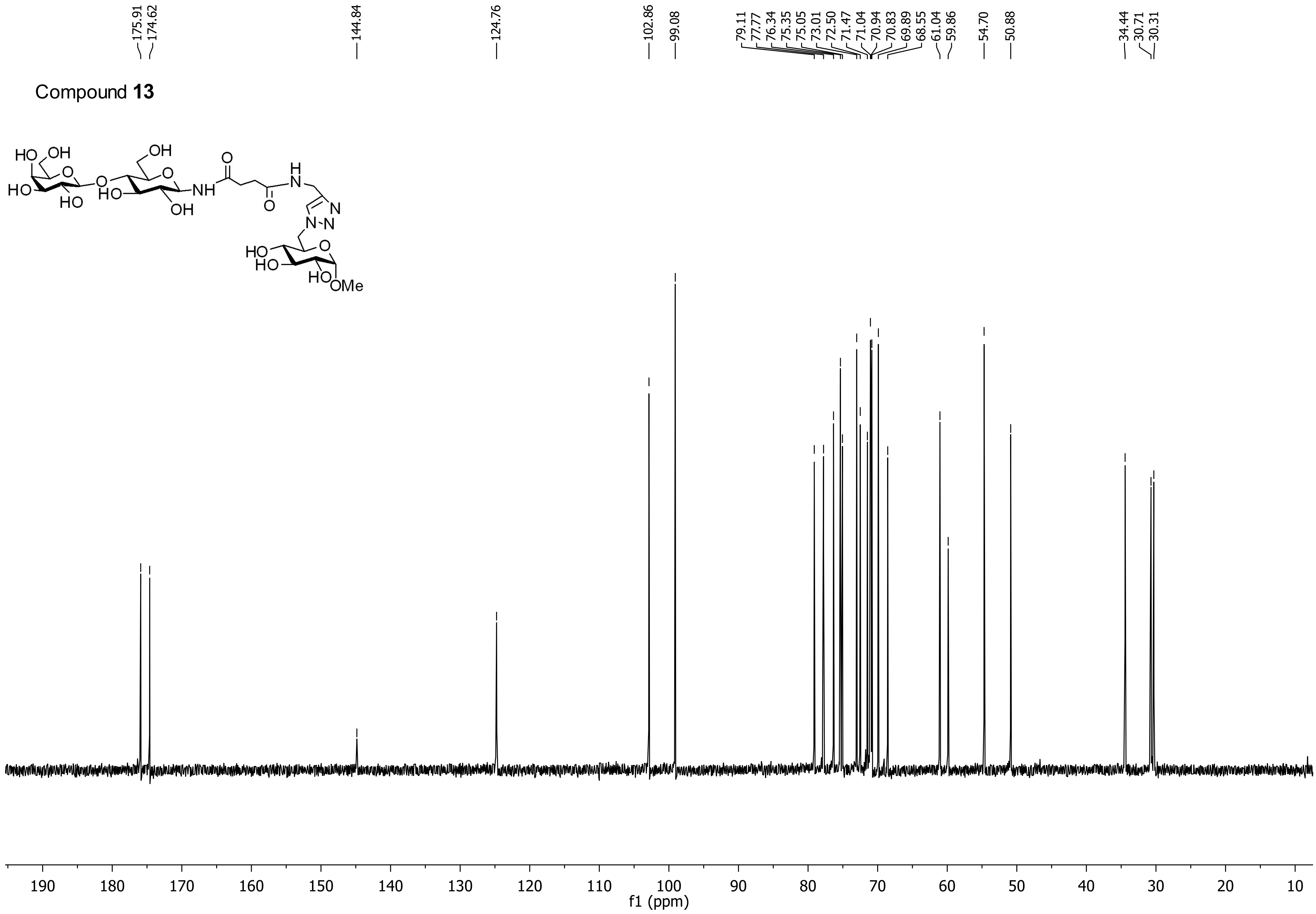
175.91
174.62
144.84
124.76
102.86
99.08
79.11
77.77
76.34
75.35
75.05
73.01
72.50
71.47
71.04
70.94
70.83
69.89
68.55
61.04
59.86
54.70
50.88
34.44
30.71
30.31
HO OH
OH
HO
HO
HO
O
O
O
NH
OH
O
O
H
N
N
N-N
HO
HO
HO
O
OMe
190 180 170 160 150 140 130 120 110 100 90 80 70 60 50 40 30 20 10
f1 (ppm)

Compound **15**

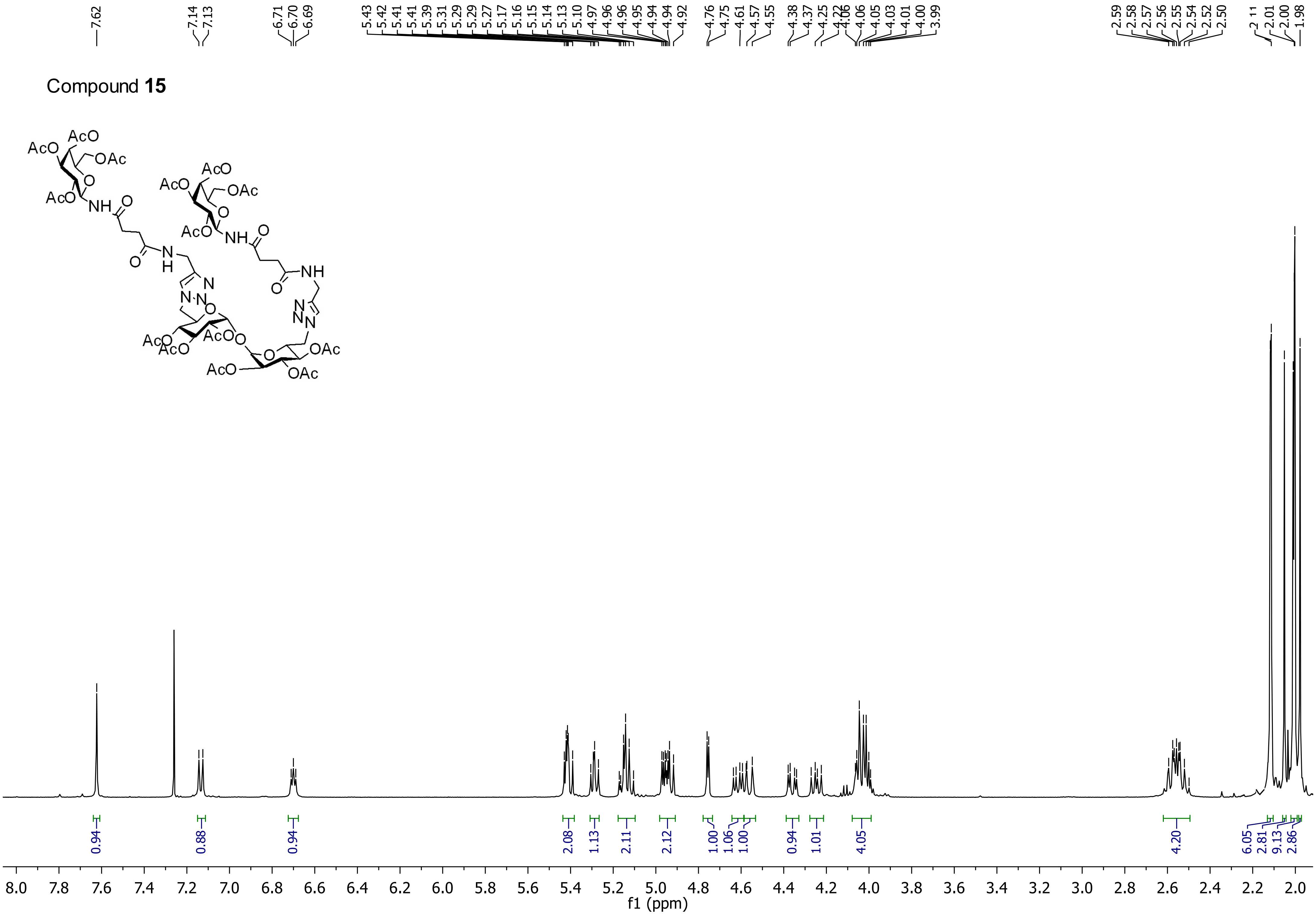
AcO
AcO
OAc
O
AcO
NH
O
N
H
N
N
N
O
AcO
AcO
AcO
O
OAc
OAc
AcO
NH
O
NH
N
N
N
OAc
OAc
AcO
f1 (ppm)

Compound **15**

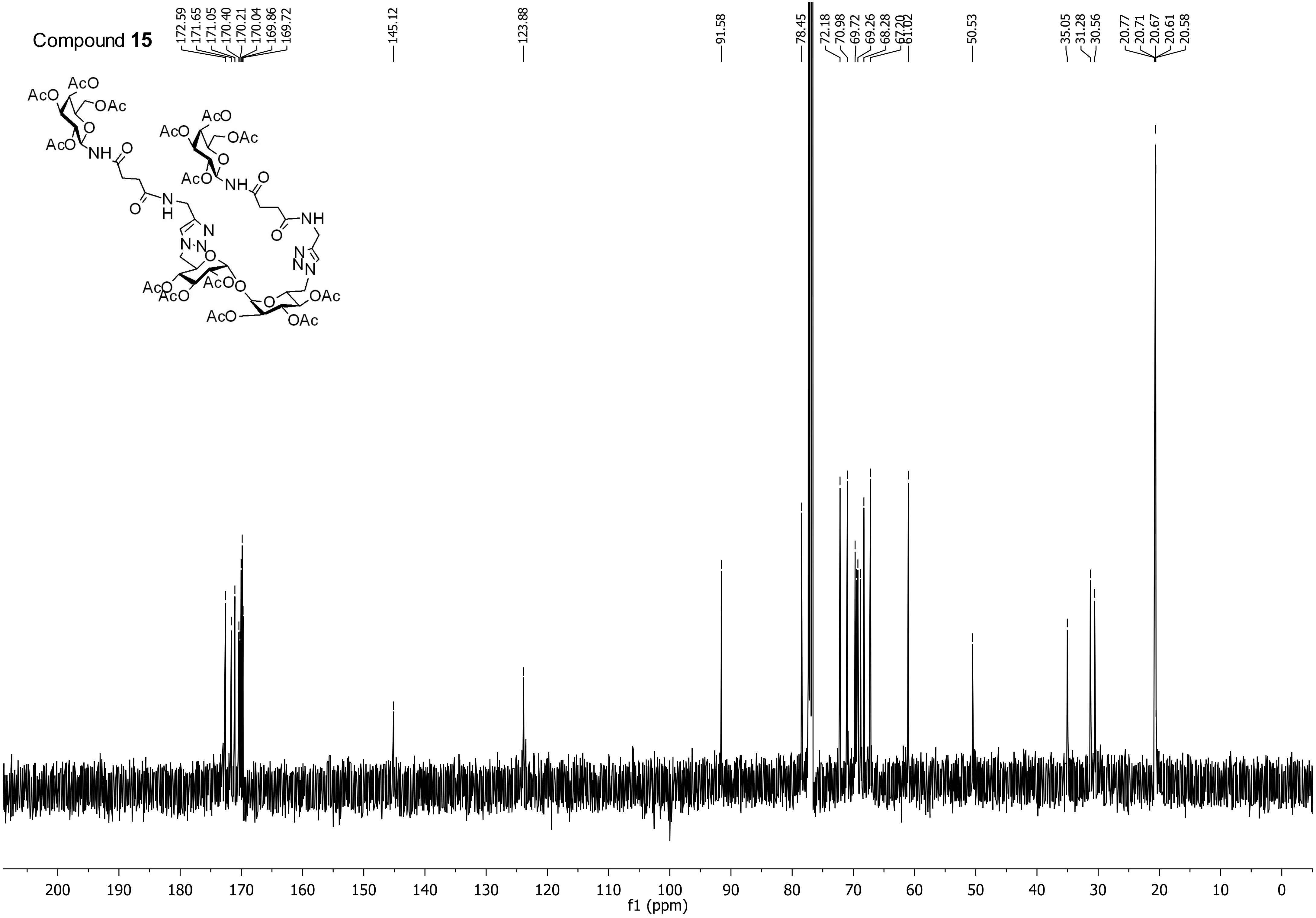
AcO
AcO
OAc
AcO
NH
O
N
H
N
N
N
O
AcO
AcO
AcO
O
NH
O
O
NH
N
N
N
O
AcO
OAc
OAc
AcO
172.59
171.65
171.05
170.40
170.21
170.04
169.86
169.72
145.12
123.88
91.58
78.45
72.18
70.98
69.72
69.26
68.28
67.20
61.02
50.53
35.05
31.28
30.56
20.77
20.71
20.67
20.61
20.58
200
190
180
170
160
150
140
130
120
110
100
90
80
70
60
50
40
30
20
10
0
f1 (ppm)

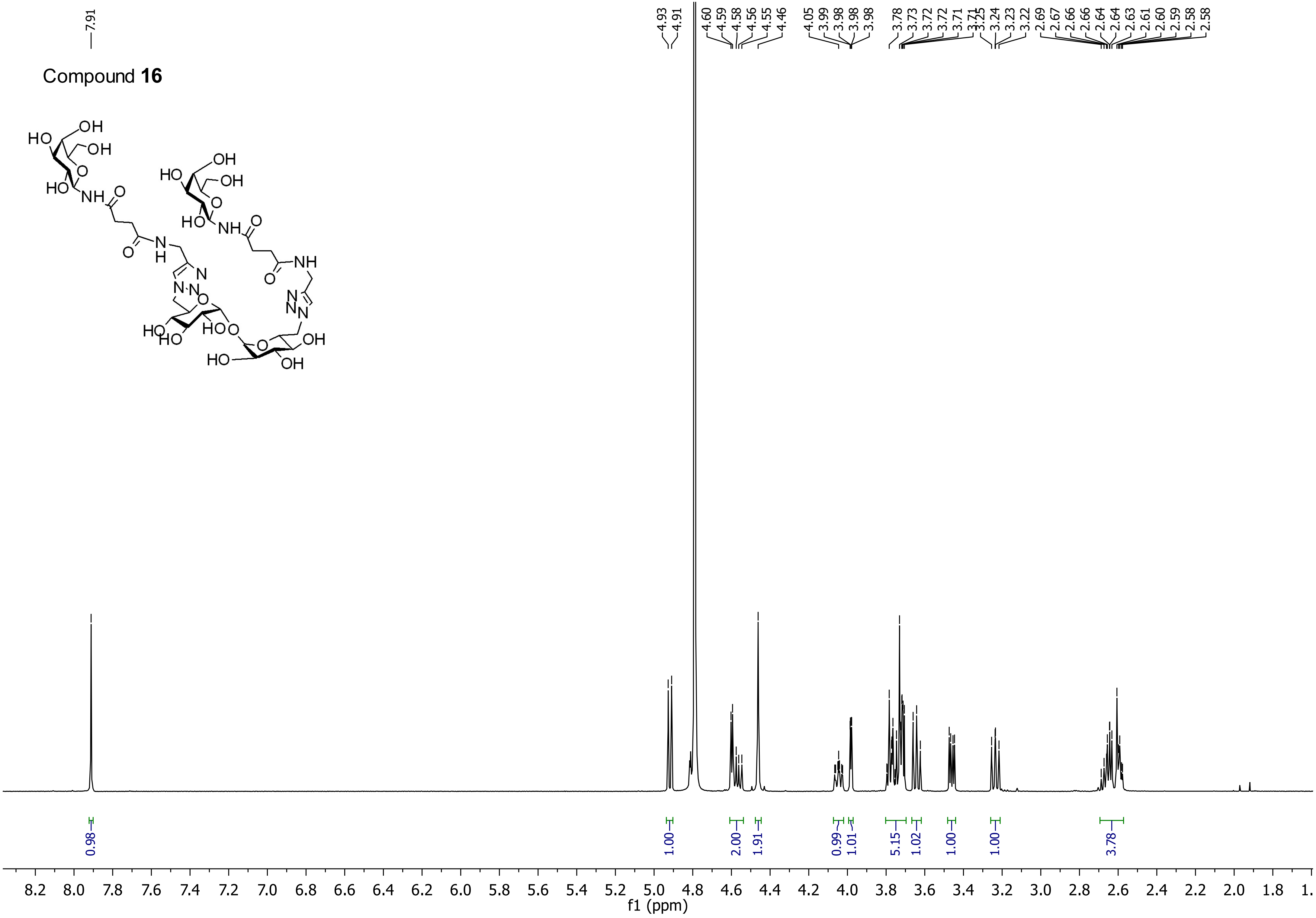

Compound 16
7.91
4.93
4.91
4.60
4.59
4.58
4.56
4.55
4.46
4.05
3.99
3.98
3.98
3.98
3.78
3.73
3.72
3.72
3.71
3.71
3.25
3.24
3.23
3.22
2.69
2.67
2.66
2.66
2.64
2.64
2.63
2.61
2.60
2.59
2.58
2.58
0.98
1.00
2.00
1.91
0.99
1.01
5.15
1.02
1.00
1.00
3.78
f1 (ppm)

Compound **16**

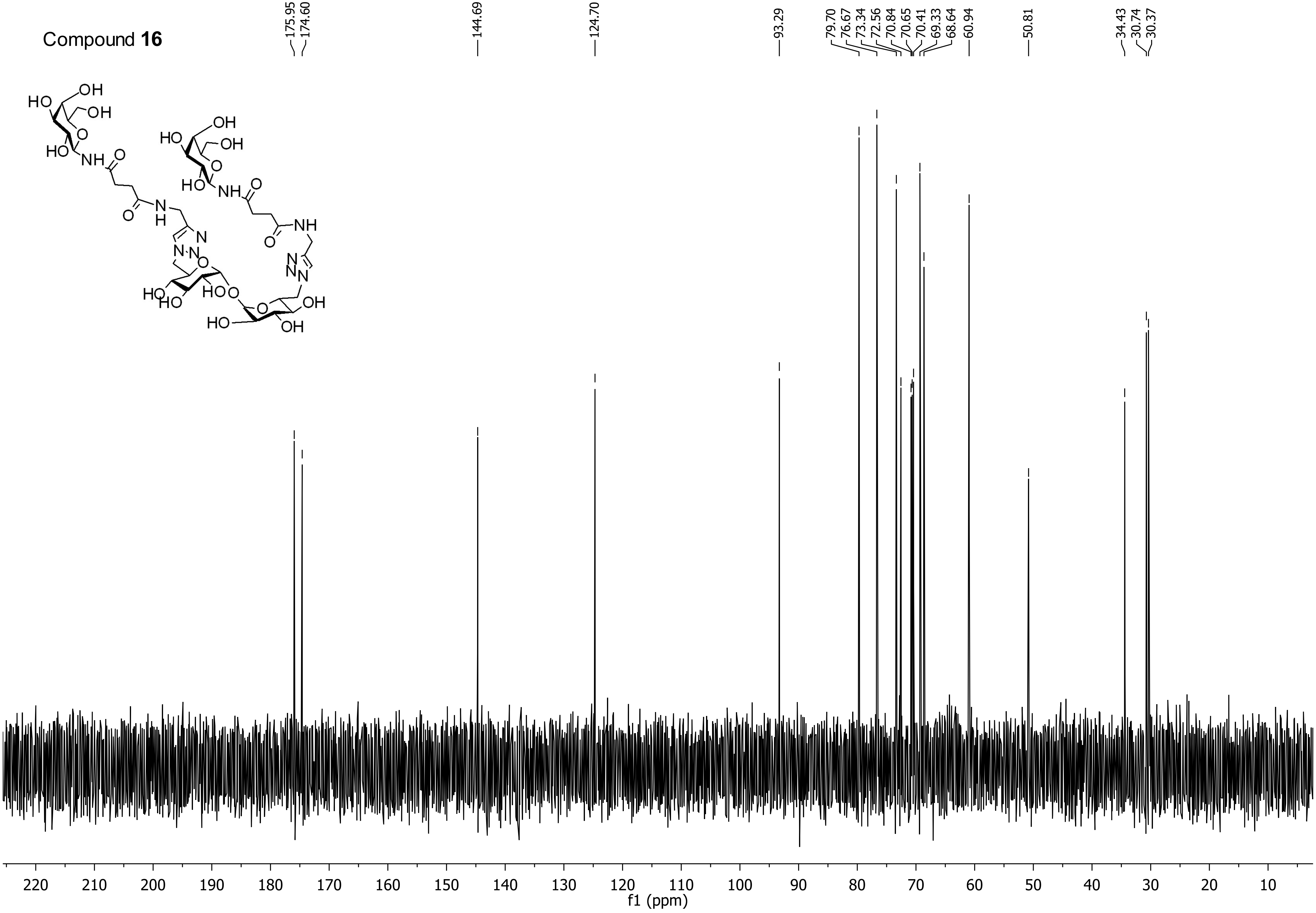

175.95
174.60
144.69
124.70
93.29
79.70
76.67
73.34
72.56
70.84
70.65
70.41
69.33
68.64
60.94
50.81
34.43
30.74
30.37
220 210 200 190 180 170 160 150 140 130 120 110 100 90 80 70 60 50 40 30 20 10
f1 (ppm)

Compound **17**

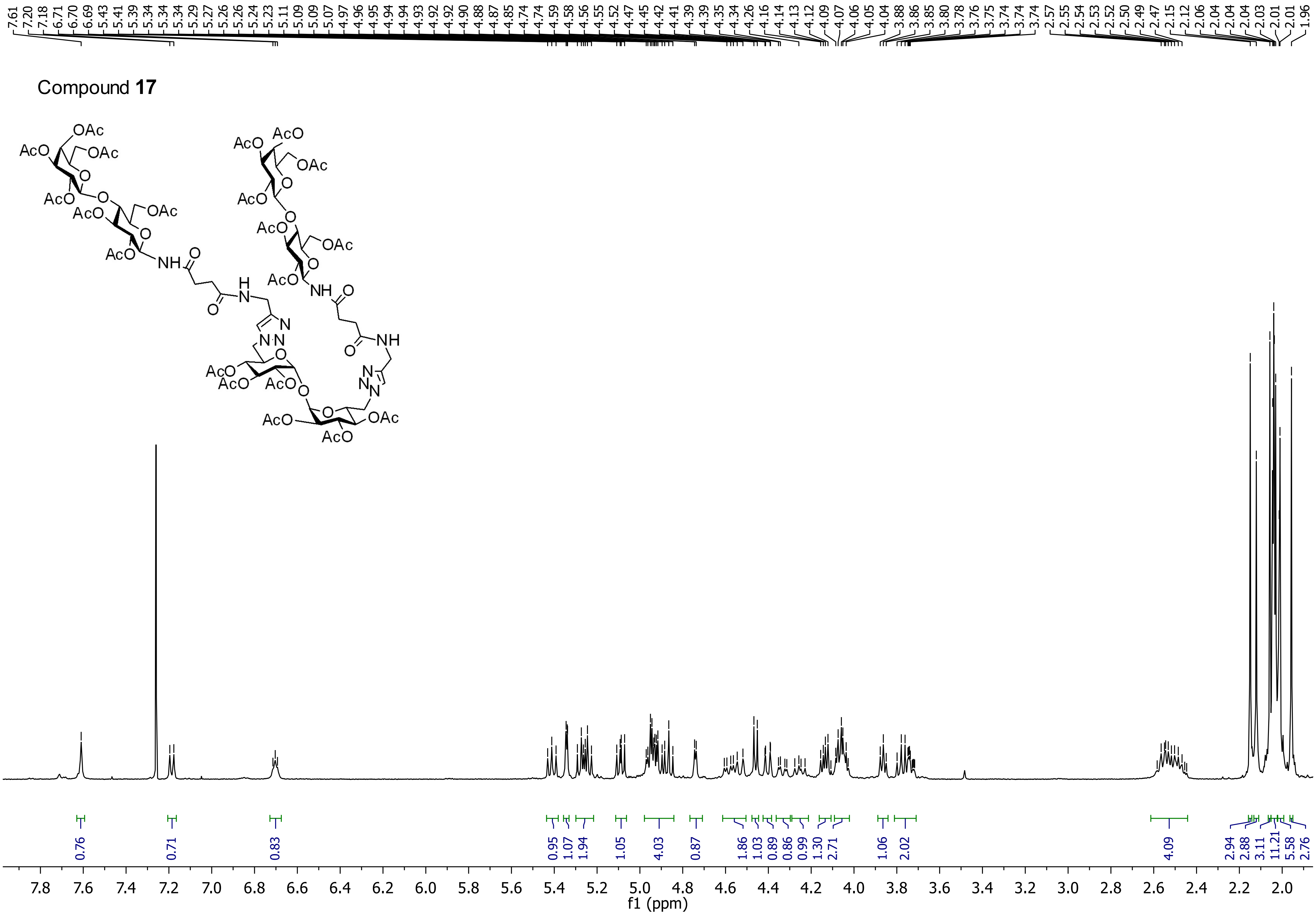

f1 (ppm)
7.8 7.6 7.4 7.2 7.0 6.8 6.6 6.4 6.2 6.0 5.8 5.6 5.4 5.2 5.0 4.8 4.6 4.4 4.2 4.0 3.8 3.6 3.4 3.2 3.0 2.8 2.6 2.4 2.2 2.0
0.76 0.71 0.83 0.95 1.07 1.94 1.05 4.03 0.87 1.86 1.03 0.89 0.86 0.99 1.30 2.71 1.06 2.02 4.09 2.94 2.88 3.11 11.21 5.58 2.76

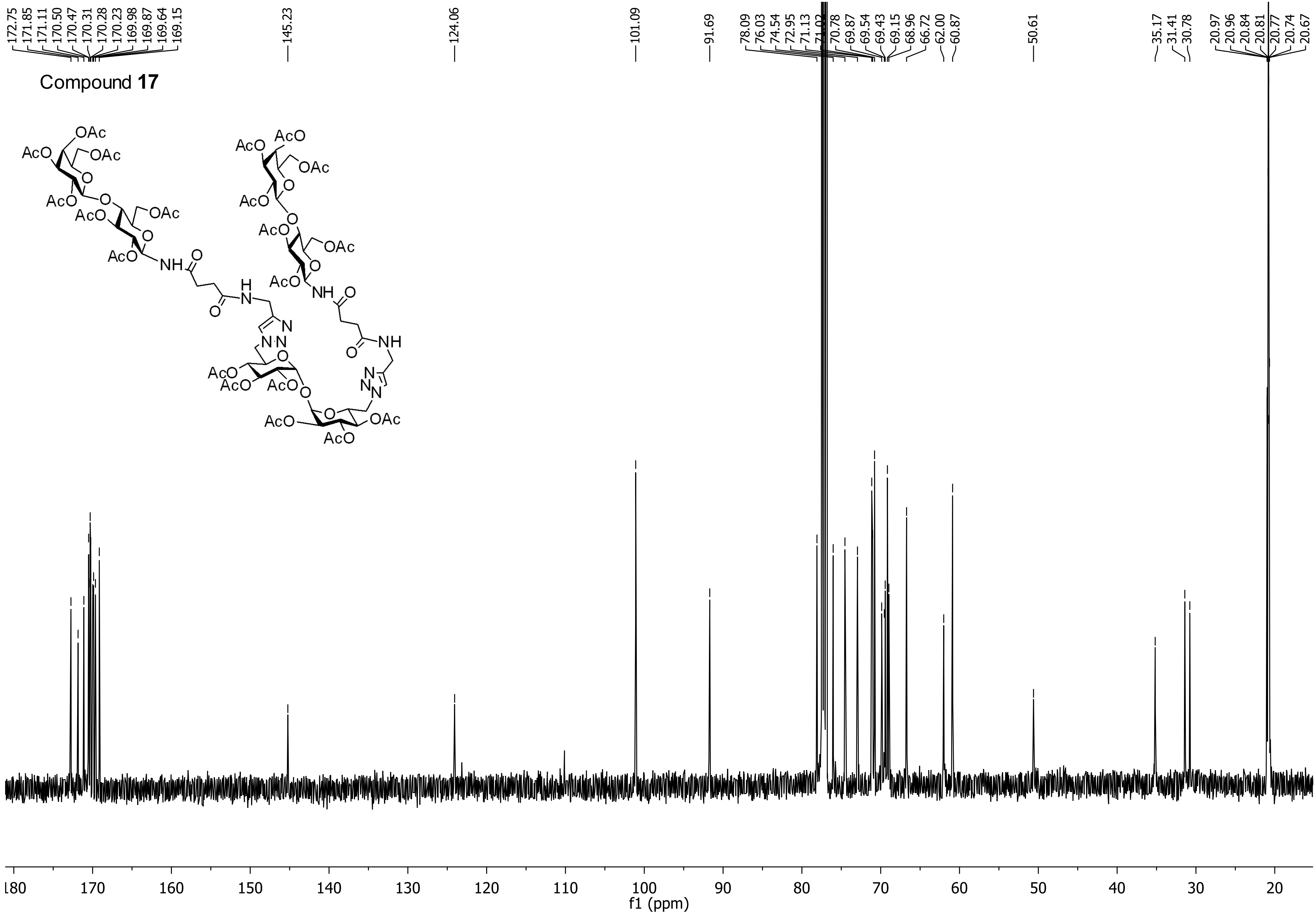

172.75
171.85
171.11
170.50
170.47
170.31
170.28
170.23
169.98
169.87
169.64
169.15
145.23
124.06
101.09
91.69
78.09
76.03
74.54
72.95
71.13
70.78
69.87
69.54
69.43
69.15
68.96
66.72
62.00
60.87
50.61
35.17
31.41
30.78
20.97
20.96
20.84
20.81
20.77
20.74
20.67
Compound 17
OAc
AcO
AcO
NH
O
H
N
AcO
180 170 160 150 140 130 120 110 100 90 80 70 60 50 40 30 20
f1 (ppm)

Compound **18**

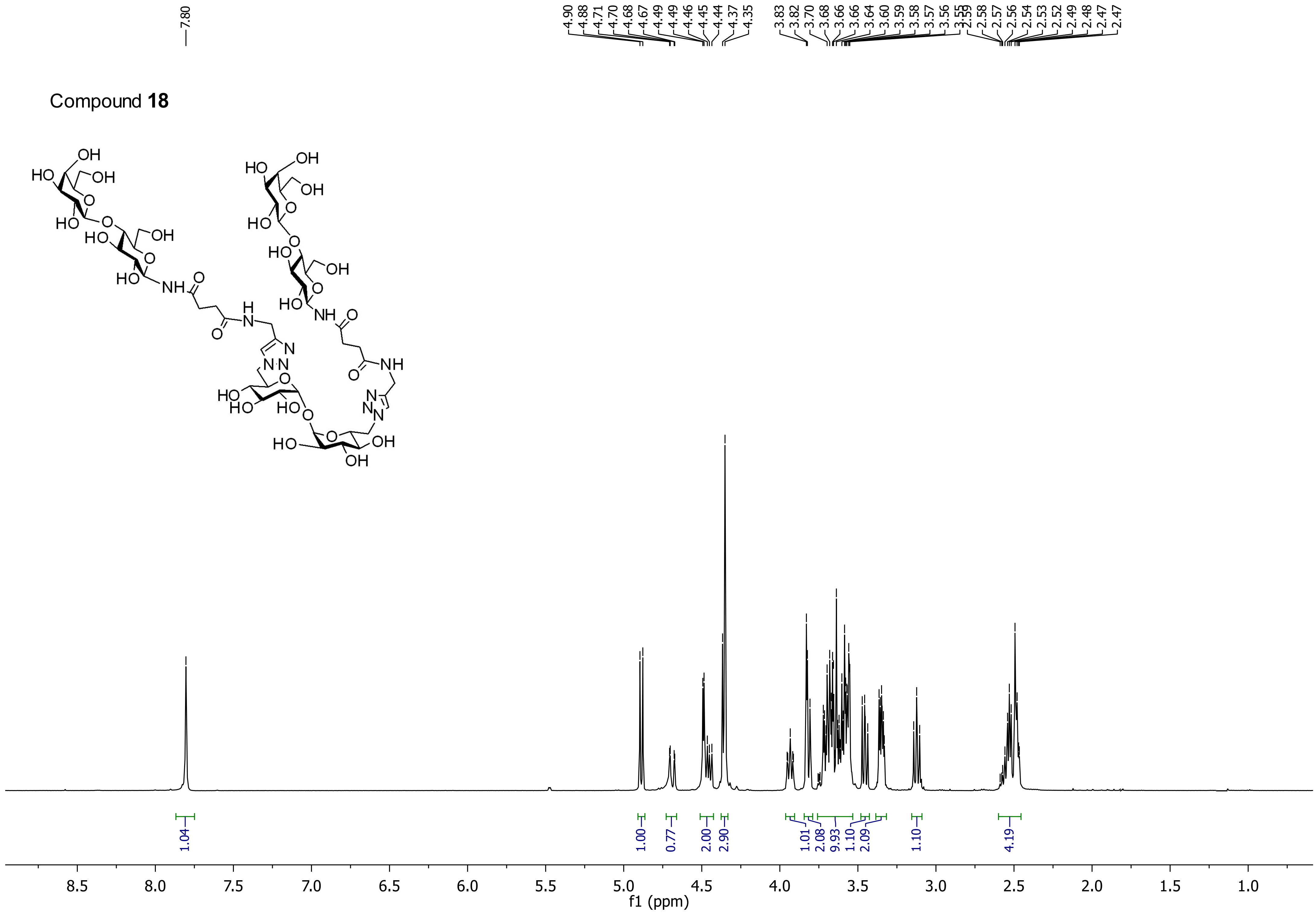
7.80
4.90 4.88 4.71 4.70 4.68 4.67 4.49 4.49 4.46 4.45 4.44 4.37 4.35
3.83 3.82 3.70 3.68 3.66 3.66 3.64 3.60 3.59 3.58 3.57 3.56 3.55 3.55
2.58 2.57 2.56 2.54 2.53 2.52 2.49 2.48 2.47 2.47
1.04
1.00
0.77
2.00
2.90
1.01
2.08
9.93
1.10
2.09
1.10
4.19
8.5 8.0 7.5 7.0 6.5 6.0 5.5 5.0 4.5 4.0 3.5 3.0 2.5 2.0 1.5 1.0
f1 (ppm)

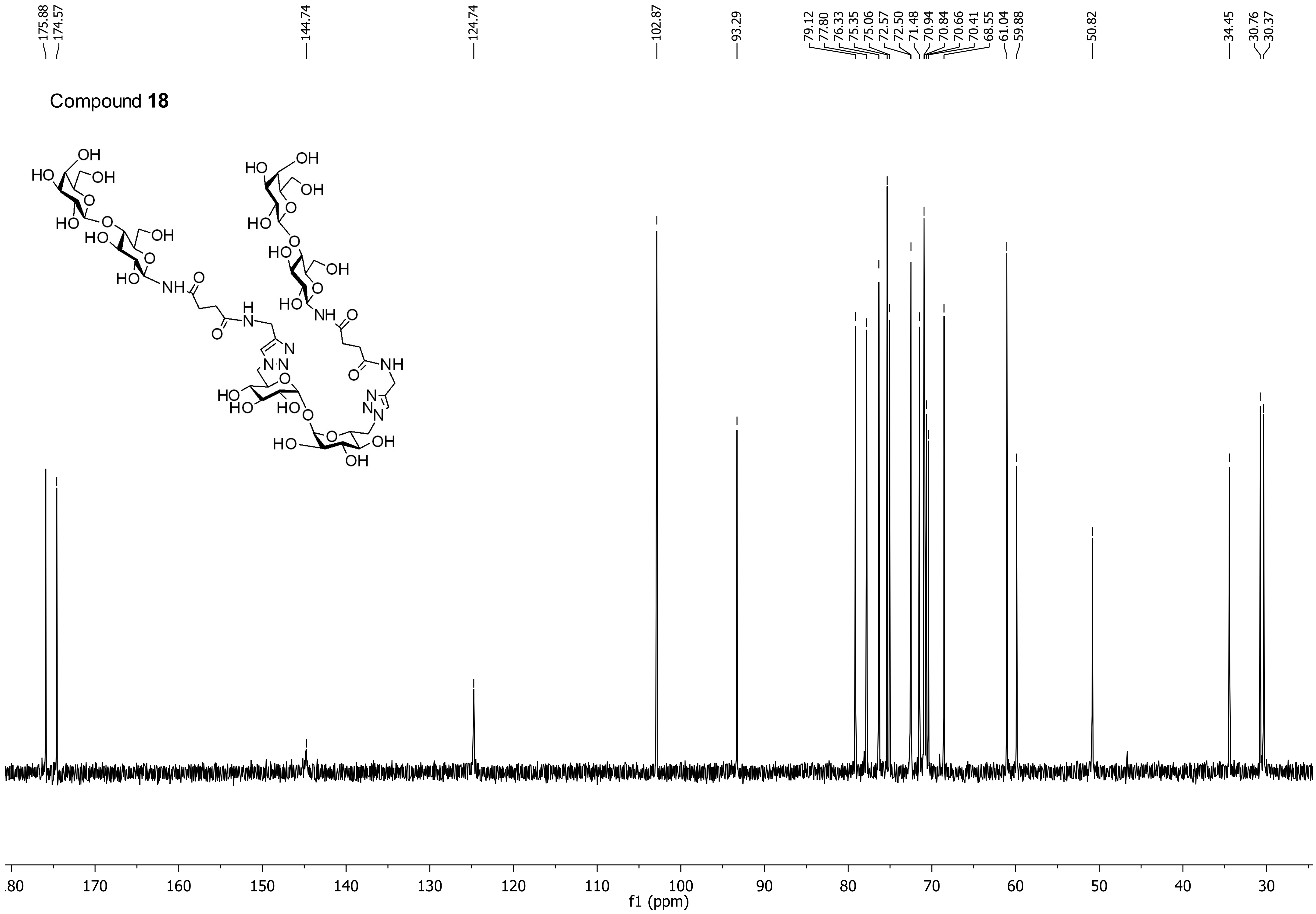
175.88
174.57
144.74
124.74
102.87
93.29
79.12
77.80
76.33
75.35
75.06
72.57
72.50
71.48
70.94
70.84
70.66
70.41
68.55
61.04
59.88
50.82
34.45
30.76
30.37
180 170 160 150 140 130 120 110 100 90 80 70 60 50 40 30
f1 (ppm)

Compound **18**

Compound **19**

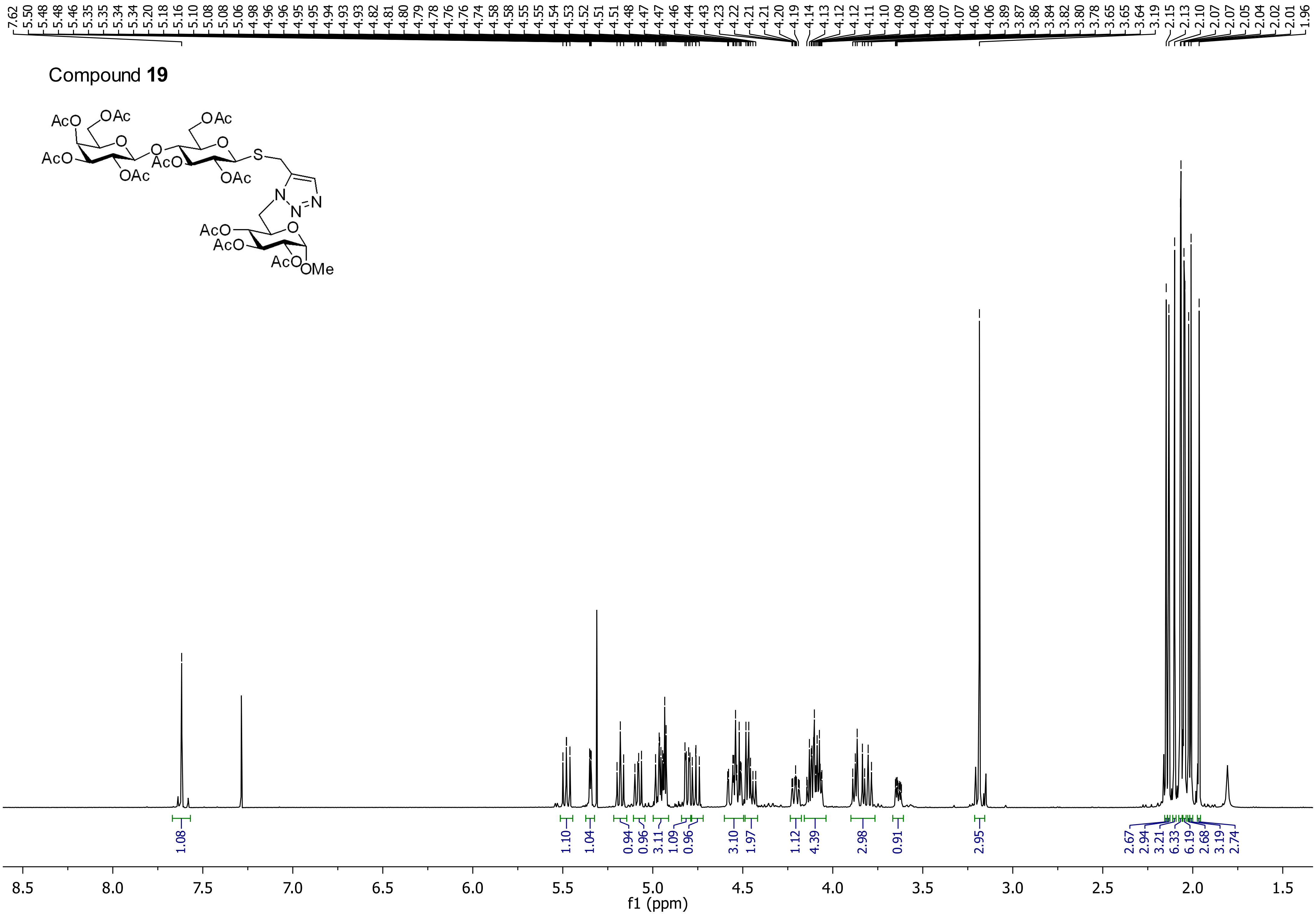
7.62
5.50
5.48
5.48
5.46
5.35
5.35
5.34
5.34
5.20
5.18
5.16
5.10
5.08
5.08
5.06
4.98
4.96
4.96
4.95
4.95
4.94
4.93
4.93
4.82
4.81
4.80
4.79
4.78
4.76
4.76
4.74
4.58
4.58
4.55
4.55
4.54
4.53
4.52
4.51
4.51
4.48
4.47
4.47
4.46
4.44
4.43
4.23
4.22
4.21
4.21
4.20
4.19
4.14
4.13
4.12
4.12
4.11
4.10
4.09
4.09
4.08
4.07
4.07
4.06
4.06
3.89
3.87
3.86
3.84
3.82
3.80
3.78
3.65
3.65
3.64
3.19
2.15
2.13
2.13
2.10
2.07
2.05
2.04
2.02
2.01
1.96
AcO
OAc
O
AcO
OAc
O
AcO
OAc
O
S
OAc
N
N
N
AcO
AcO
O
AcO
OMe
1.08
1.10
1.04
0.94
0.96
3.11
1.09
0.96
3.10
1.97
1.12
4.39
2.98
0.91
2.95
2.67
2.94
3.21
6.33
6.19
2.68
3.19
2.74
8.5
8.0
7.5
7.0
6.5
6.0
5.5
5.0
4.5
4.0
3.5
3.0
2.5
2.0
1.5
f1 (ppm)

Compound **19**

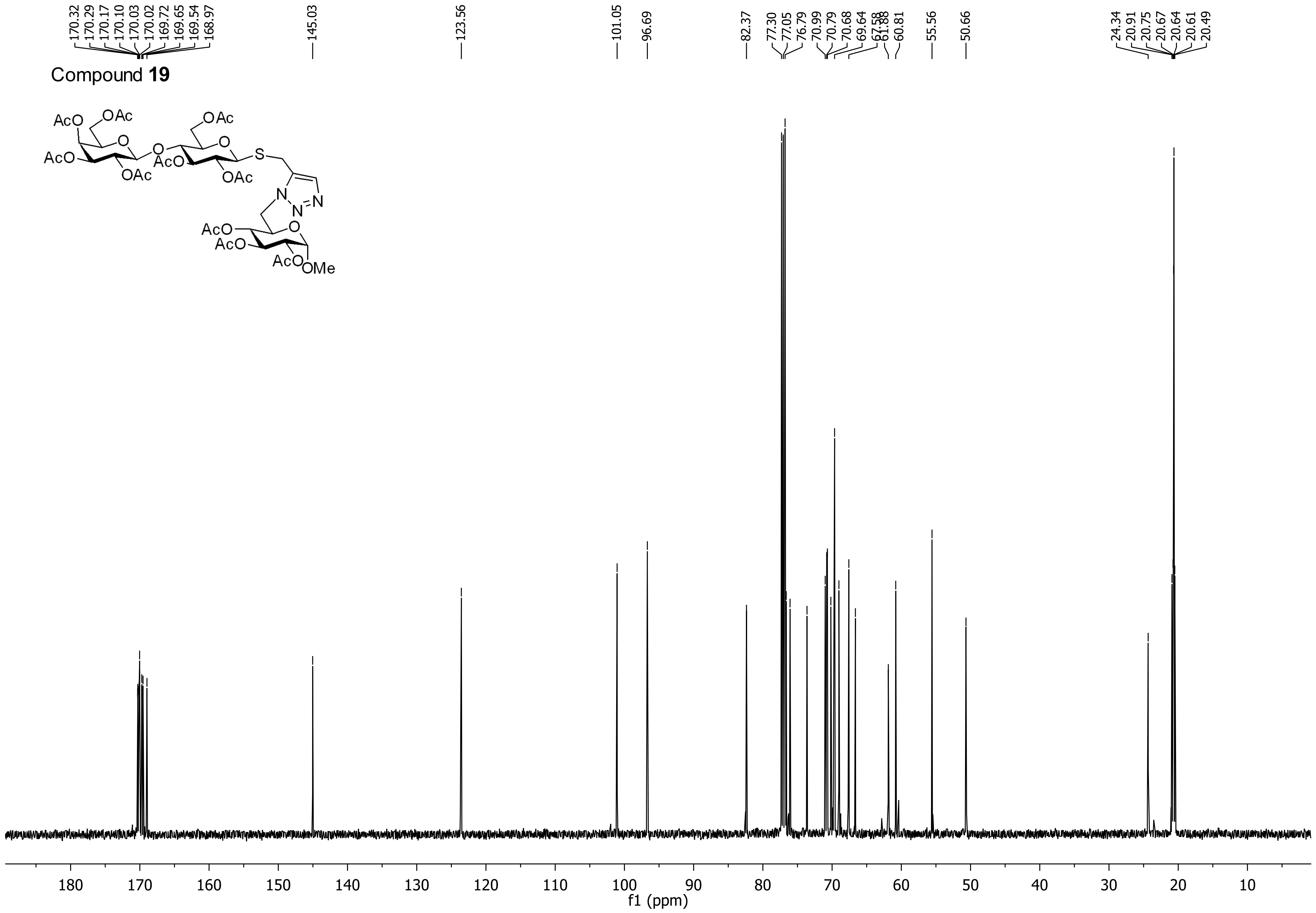
AcO
OAc
AcO
OAc
AcO
OAc
OAc
S
N
N
N
AcO
AcO
AcO
OMe
170.32
170.29
170.17
170.10
170.03
170.02
169.72
169.65
169.54
168.97
145.03
123.56
101.05
96.69
82.37
77.30
77.05
76.79
70.99
70.79
70.68
69.64
67.58
61.88
60.81
55.56
50.66
24.34
20.91
20.75
20.67
20.64
20.61
20.49
180
170
160
150
140
130
120
110
100
90
80
70
60
50
40
30
20
10
f1 (ppm)

Compound **20**

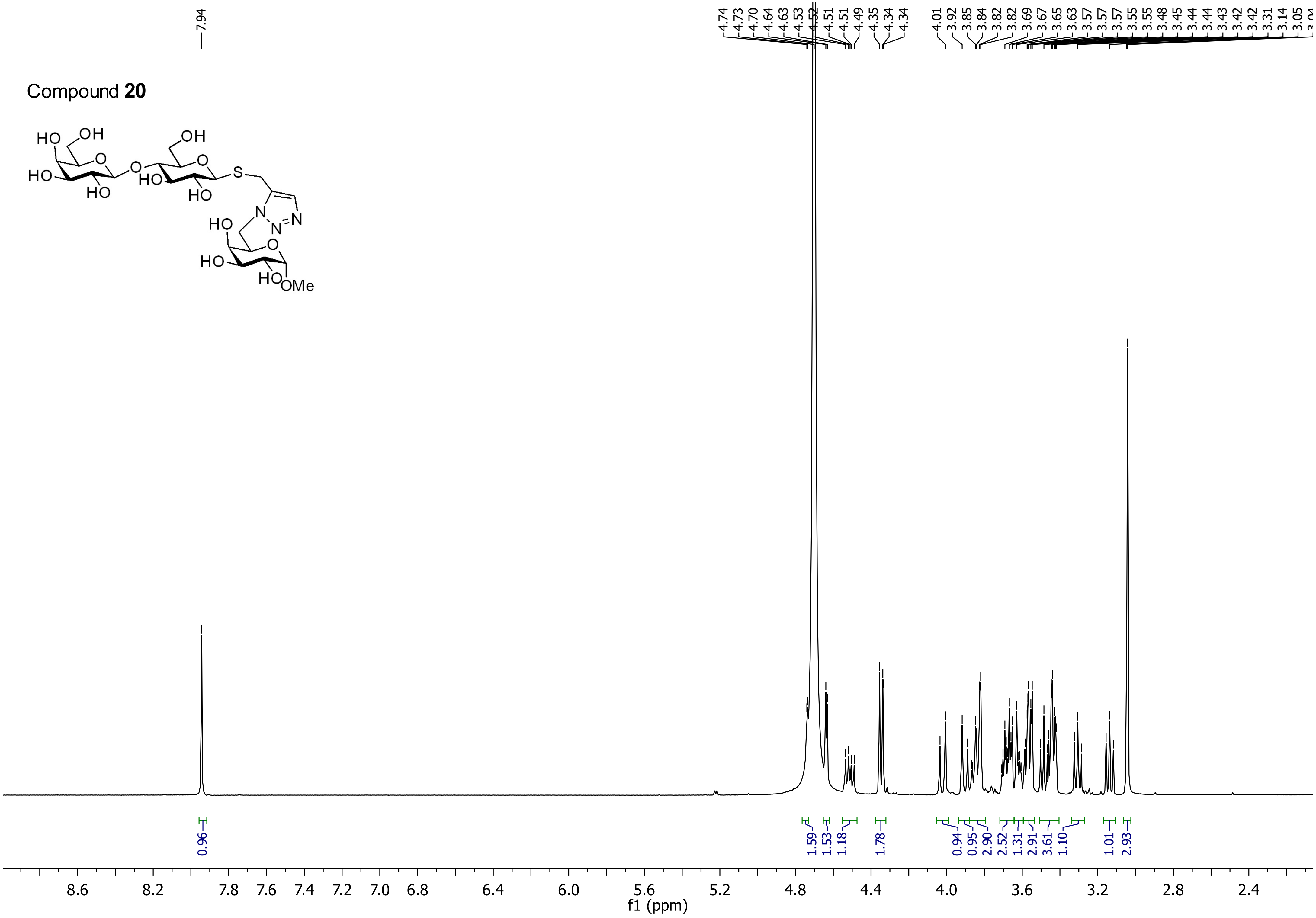

Compound **20**

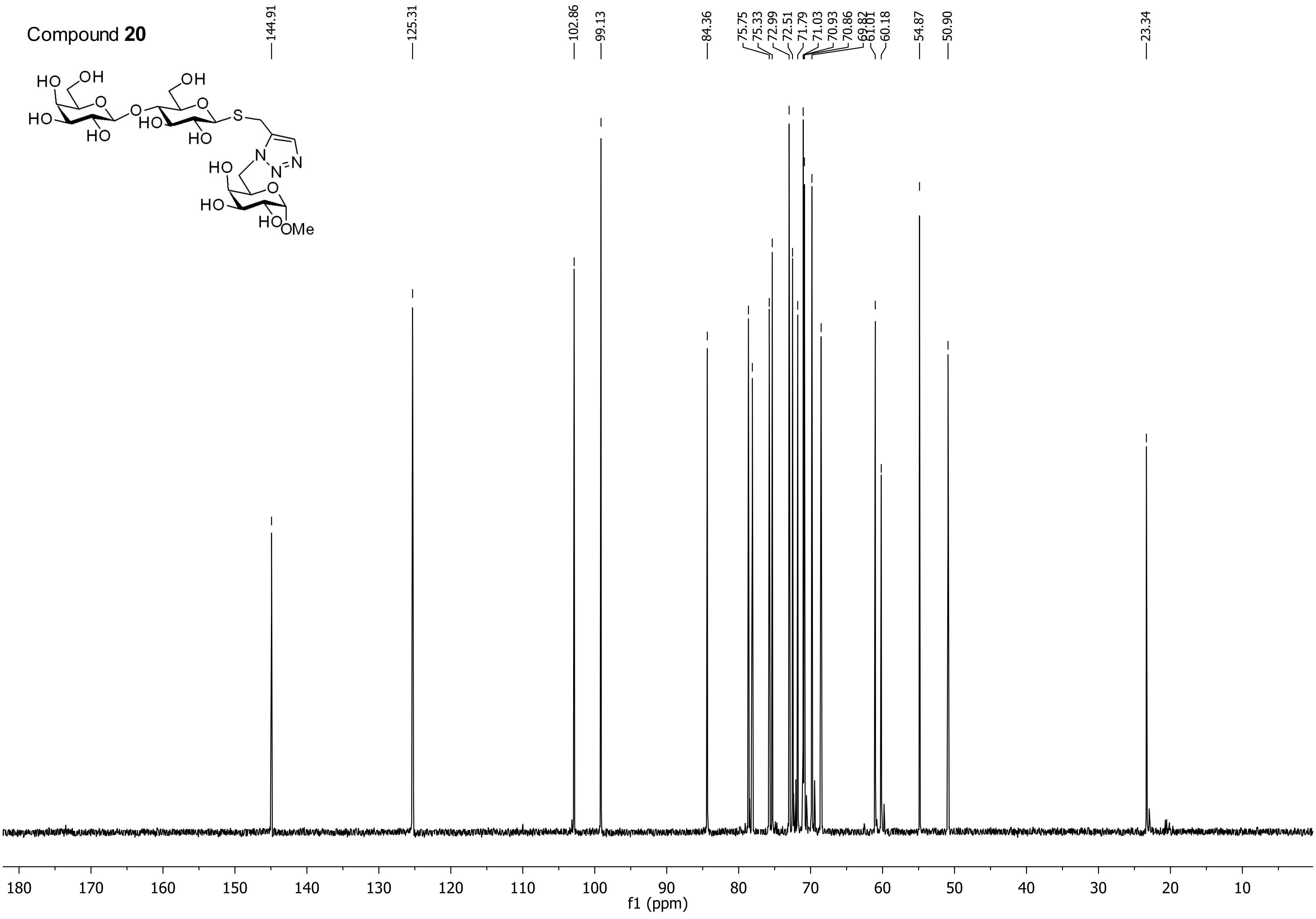

144.91
125.31
102.86
99.13
84.36
75.75
75.33
72.99
72.51
71.79
71.03
70.93
70.86
69.82
61.01
60.18
54.87
50.90
23.34
HO
OH
O
HO
HO
O
OH
O
HO
HO
S
N
N
N
HO
O
HO
HO
OMe
180
170
160
150
140
130
120
110
100
90
80
70
60
50
40
30
20
10
f1 (ppm)

Compound **21**

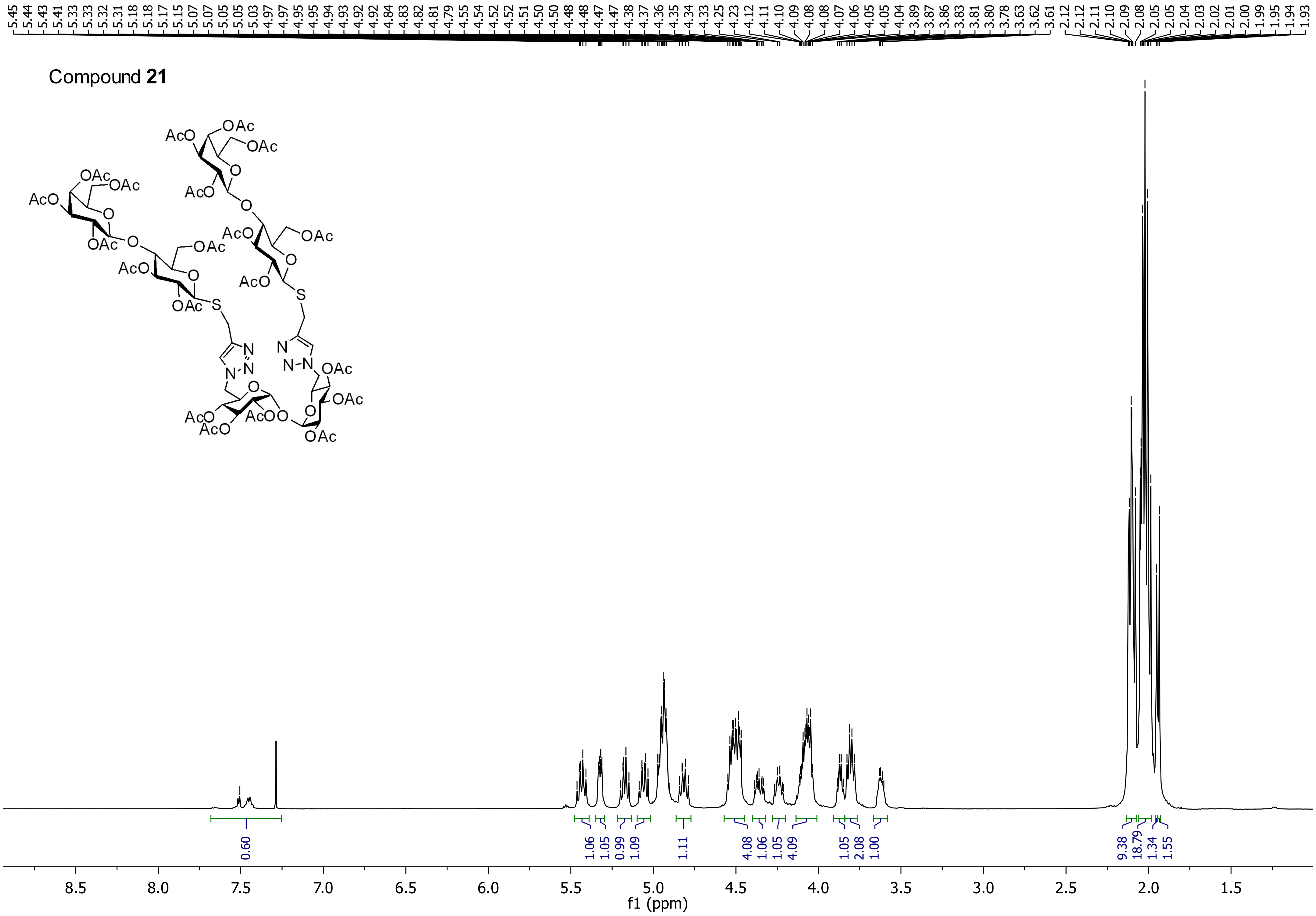

5.45 5.44 5.43 5.41 5.33 5.33 5.32 5.31 5.18 5.18 5.17 5.15 5.07 5.07 5.05 5.03 4.97 4.97 4.95 4.95 4.94 4.93 4.92 4.92 4.84 4.83 4.82 4.81 4.79 4.55 4.54 4.52 4.52 4.51 4.50 4.48 4.48 4.47 4.47 4.38 4.37 4.36 4.35 4.34 4.33 4.25 4.23 4.12 4.11 4.10 4.09 4.08 4.08 4.07 4.06 4.05 4.05 4.04 3.89 3.87 3.86 3.83 3.81 3.80 3.78 3.63 3.62 3.61 2.12 2.12 2.11 2.10 2.09 2.08 2.05 2.05 2.04 2.03 2.02 2.01 2.00 1.99 1.95 1.94 1.93
OAc
AcO
O
S
N
N-N
0.60
1.06
1.05
0.99
1.09
1.11
4.08
1.06
1.05
4.09
1.05
2.08
1.00
9.38
18.79
1.34
1.55
8.5
8.0
7.5
7.0
6.5
6.0
5.5
5.0
4.5
4.0
3.5
3.0
2.5
2.0
1.5
f1 (ppm)

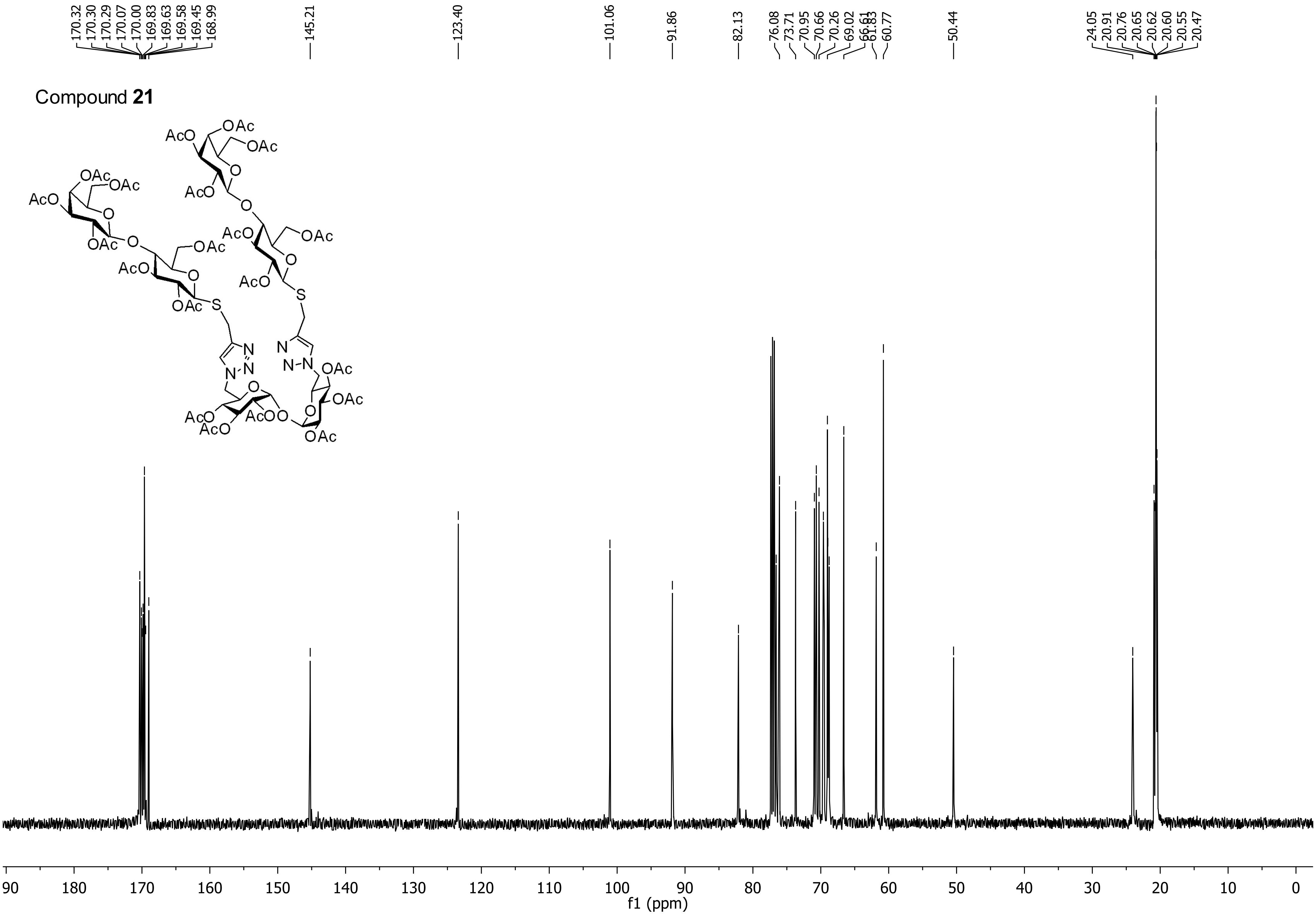
170.32
170.30
170.29
170.07
170.00
169.83
169.63
169.58
169.45
168.99
145.21
123.40
101.06
91.86
82.13
76.08
73.71
70.95
70.66
70.26
69.02
66.61
61.83
60.77
50.44
24.05
20.91
20.76
20.65
20.62
20.60
20.55
20.47
AcO
OAc
OAc
AcO
OAc
OAc
AcO
AcO
OAc
OAc
AcO
AcO
OAc
AcO
S
S
N
N
N
N
N
N
OAc
OAc
OAc
AcO
AcO
AcO
90
180
170
160
150
140
130
120
110
100
90
80
70
60
50
40
30
20
10
0
f1 (ppm)

Compound **21**

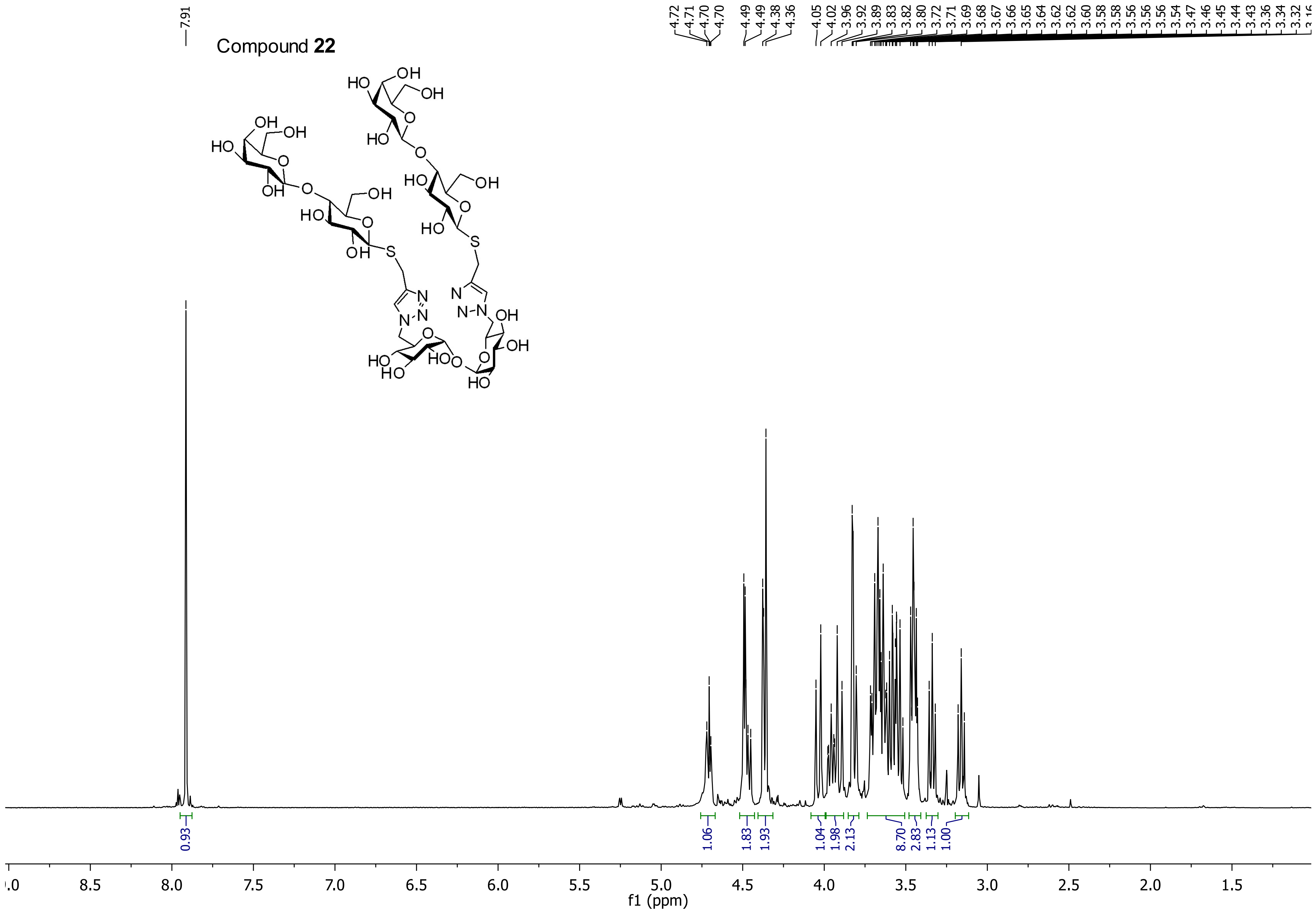

Compound 22
7.91
4.72
4.71
4.70
4.70
4.49
4.49
4.38
4.36
4.05
4.02
3.96
3.92
3.89
3.83
3.82
3.80
3.72
3.71
3.69
3.68
3.67
3.66
3.65
3.64
3.62
3.62
3.60
3.58
3.58
3.56
3.56
3.56
3.54
3.47
3.46
3.45
3.44
3.43
3.36
3.34
3.32
0.93
1.06
1.83
1.93
1.04
1.98
2.13
8.70
2.83
1.13
1.00
8.5
8.0
7.5
7.0
6.5
6.0
5.5
5.0
4.5
4.0
3.5
3.0
2.5
2.0
1.5
f1 (ppm)

Compound **22**

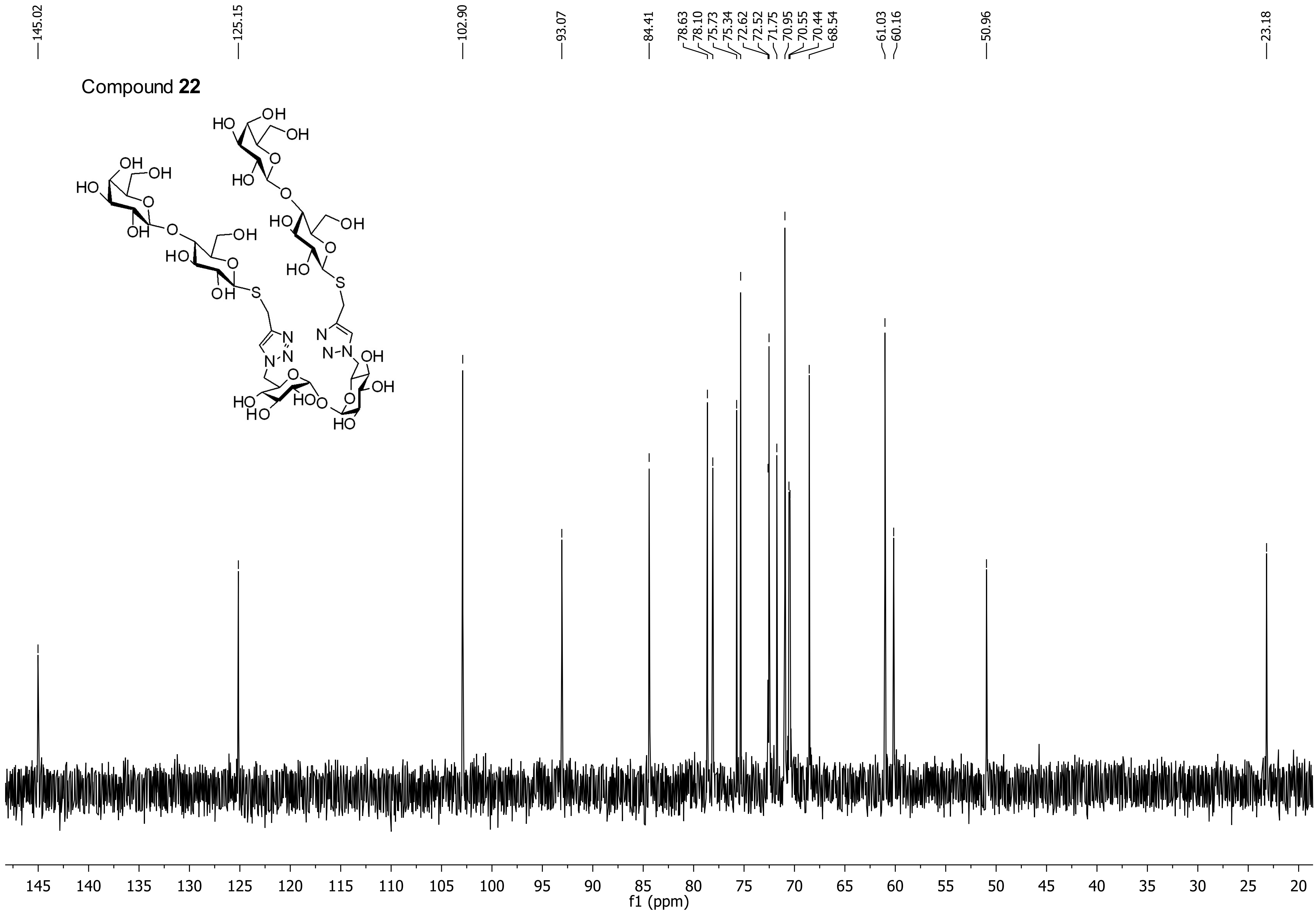

145.02
125.15
102.90
93.07
84.41
78.63
78.10
75.73
75.34
72.62
72.52
71.75
70.95
70.55
70.44
68.54
61.03
60.16
50.96
23.18
f1 (ppm)

Compound **25**

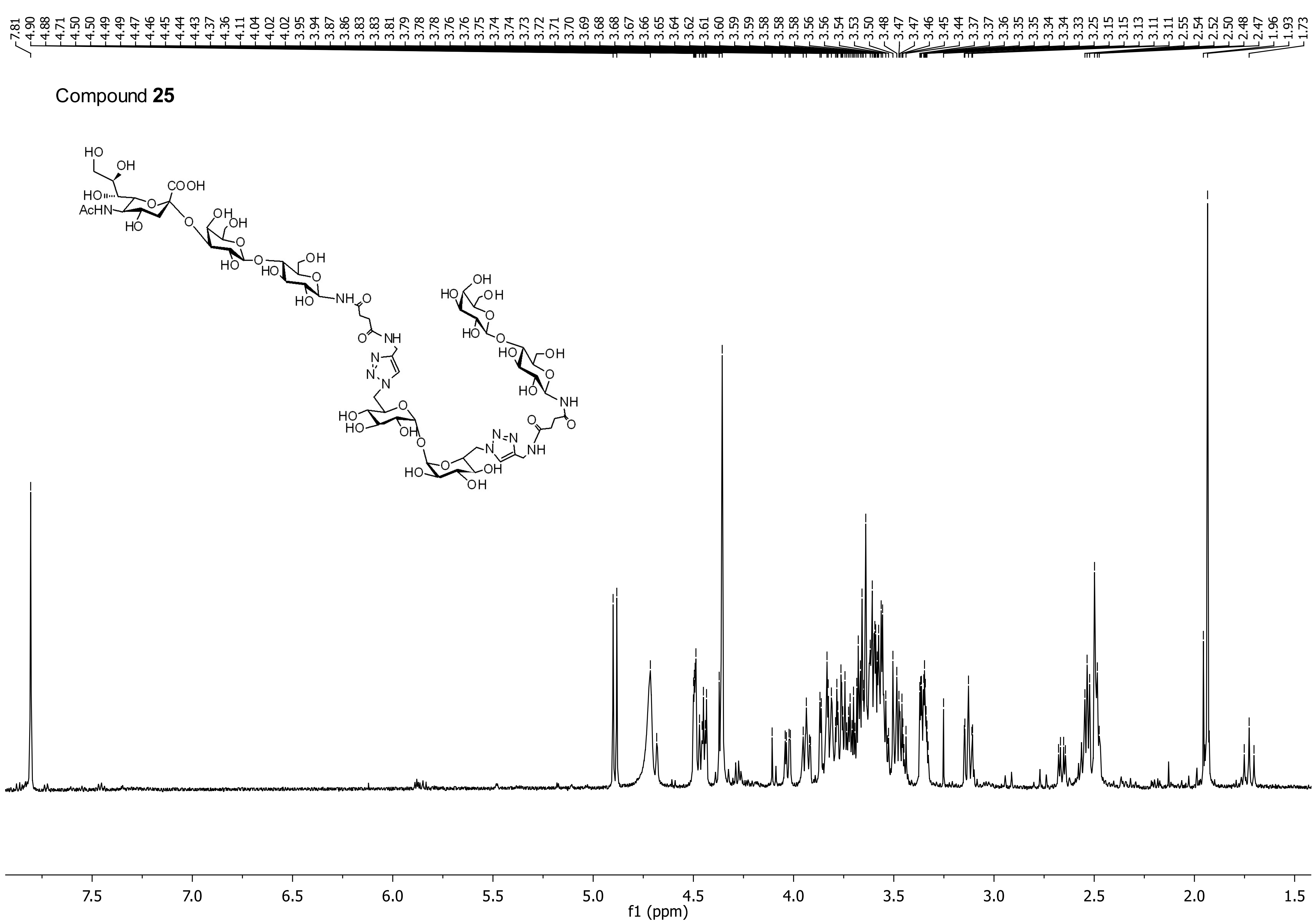
7.81
4.90
4.88
4.71
4.50
4.50
4.49
4.49
4.47
4.46
4.45
4.44
4.43
4.37
4.36
4.11
4.04
4.02
4.02
3.95
3.94
3.87
3.86
3.83
3.83
3.81
3.79
3.78
3.78
3.76
3.76
3.75
3.74
3.74
3.73
3.72
3.71
3.70
3.69
3.68
3.68
3.67
3.66
3.65
3.64
3.62
3.61
3.60
3.59
3.59
3.58
3.58
3.56
3.56
3.54
3.53
3.50
3.48
3.47
3.47
3.46
3.45
3.44
3.37
3.37
3.36
3.35
3.35
3.34
3.34
3.33
3.25
3.15
3.15
3.13
3.11
3.11
2.55
2.54
2.52
2.50
2.48
2.47
1.96
1.93
1.73
7.5
7.0
6.5
6.0
5.5
5.0
4.5
4.0
3.5
3.0
2.5
2.0
1.5
f1 (ppm)

Compound **25**

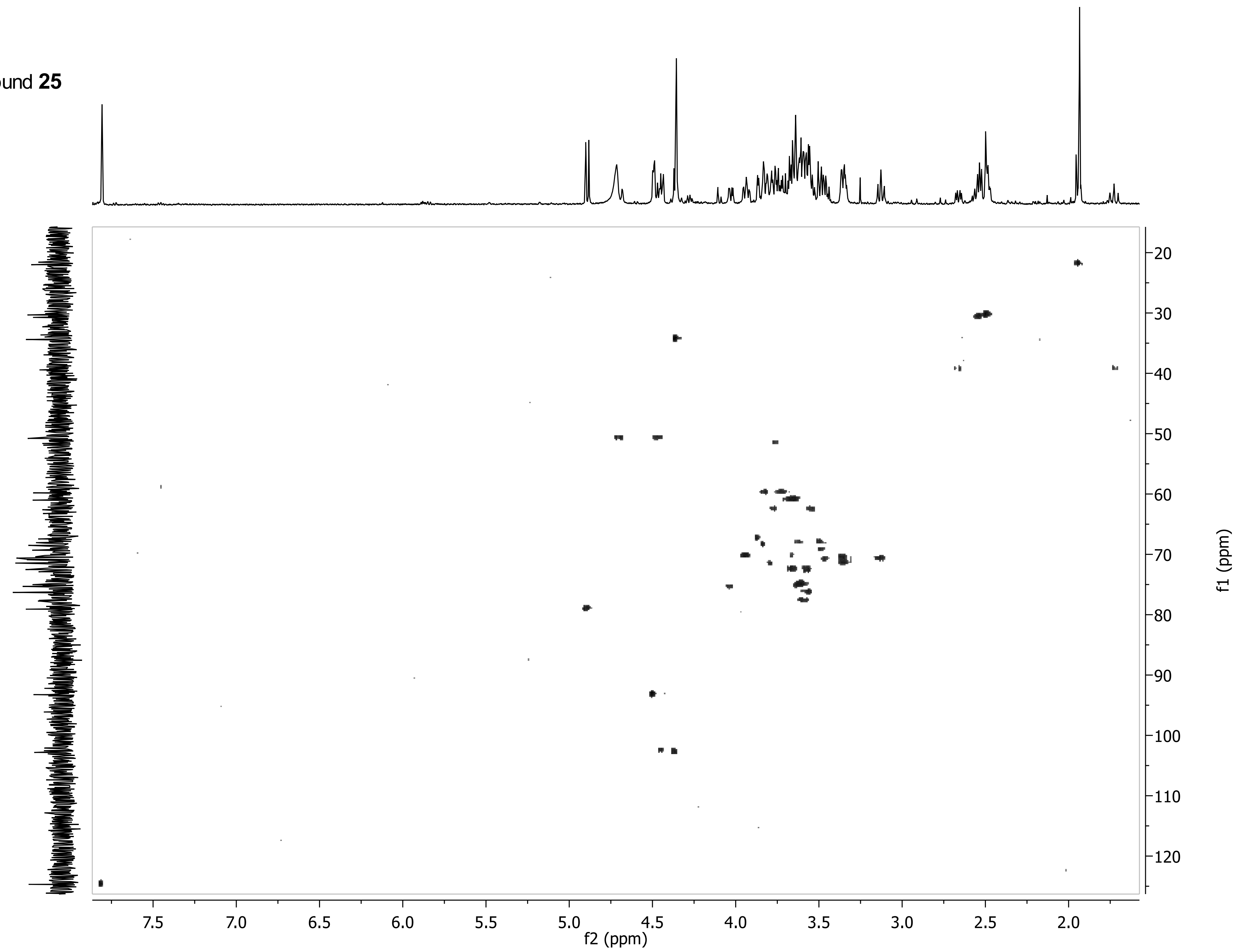

f2 (ppm)
f1 (ppm)
7.5
7.0
6.5
6.0
5.5
5.0
4.5
4.0
3.5
3.0
2.5
2.0
20
30
40
50
60
70
80
90
100
110
120

Compound **25**

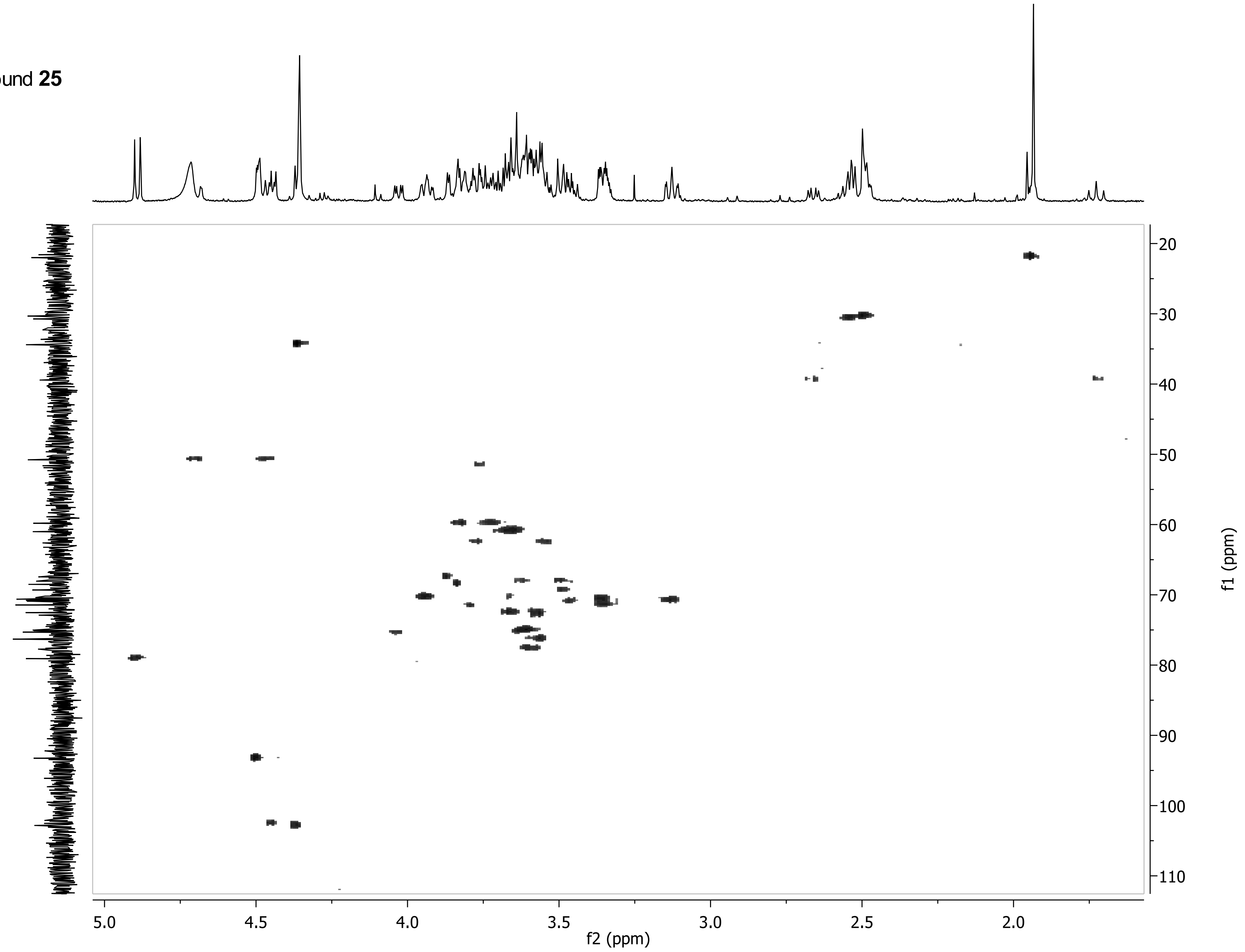

Compound **25**

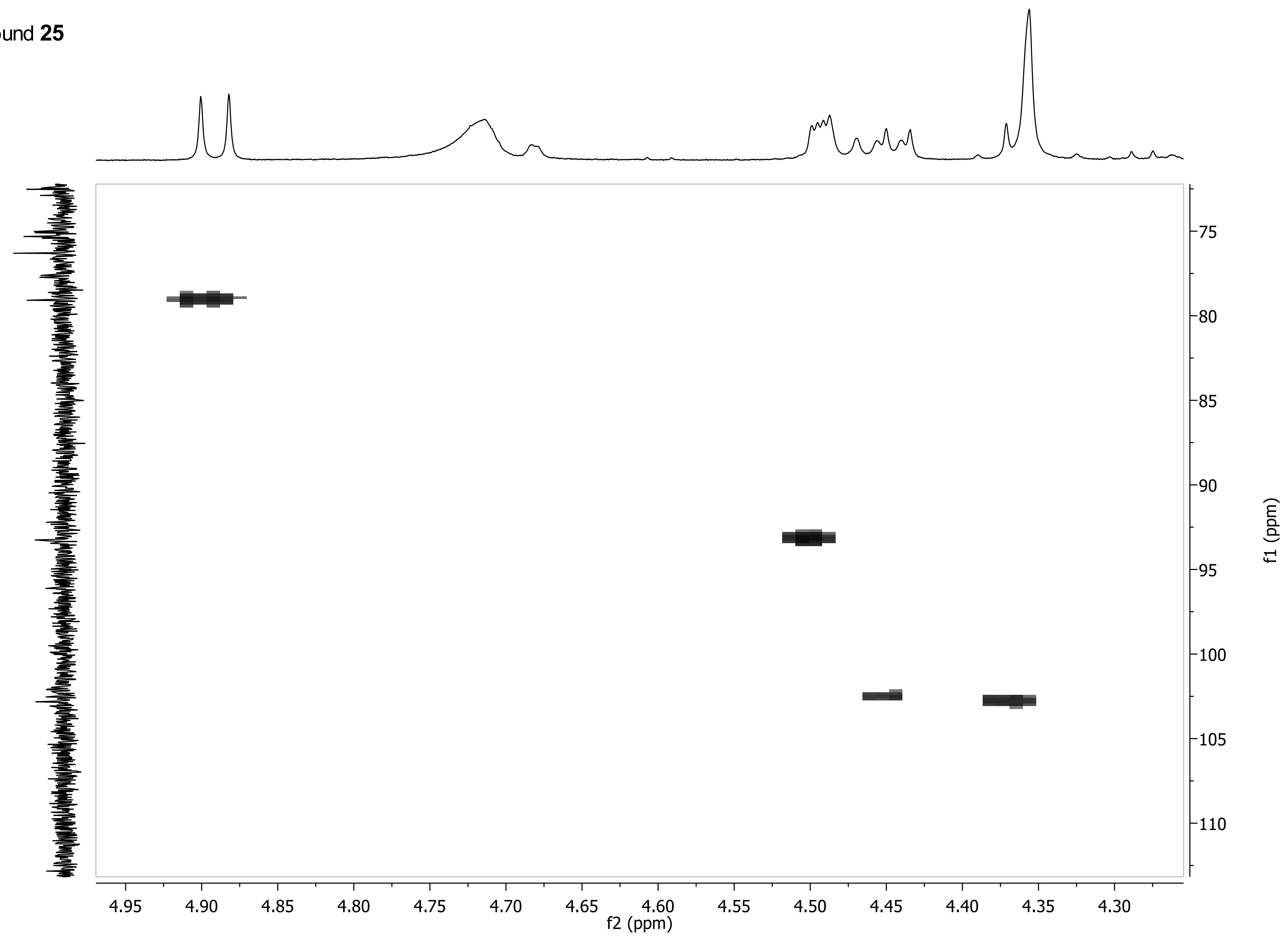

75
80
85
90
95
100
105
110
f1 (ppm)
4.95
4.90
4.85
4.80
4.75
4.70
4.65
4.60
4.55
4.50
4.45
4.40
4.35
4.30
f2 (ppm)
